# Supplementary material for: Basal Protrusions Mediate Spatiotemporal Patterns of Spinal Neuron Differentiation
Source: Dev Cell. 2019 Jun 17;49(6):907–919.e10. doi: 10.1016/j.devcel.2019.05.035 (PMC6584357; doi:10.1016/j.devcel.2019.05.035)
Supplement: Document S1. Figures S1–S8 and Data S1 [file mmc1.pdf]

Developmental Cell, Volume *49*

Supplemental Information

Basal Protrusions Mediate Spatiotemporal Patterns of Spinal Neuron Differentiation

Zena Hadjivasiliou, Rachel E. Moore, Rebecca McIntosh, Gabriel L. Galea, Jonathan D.W. Clarke, and Paula Alexandre

Figure S1

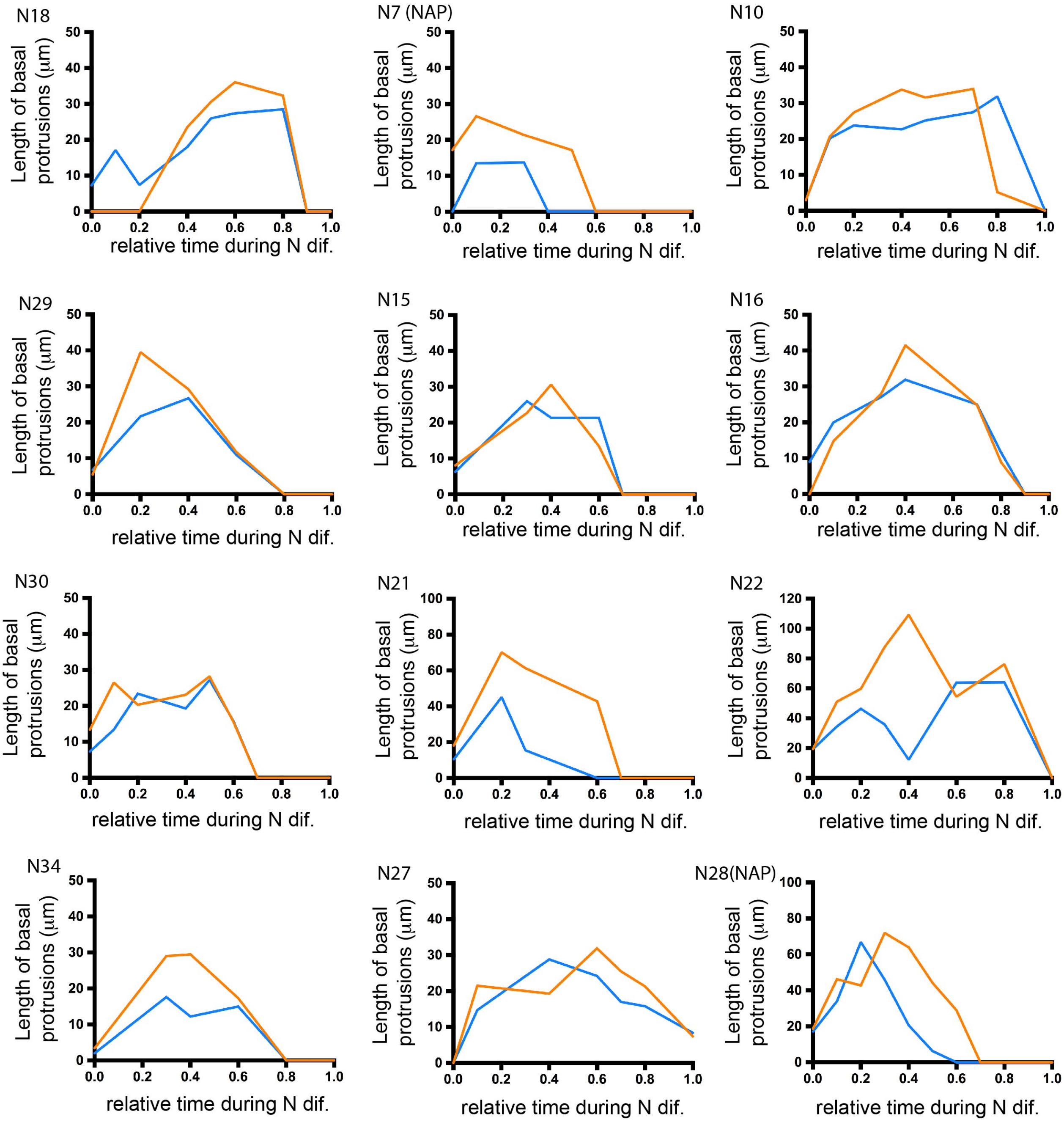

The dynamics of basal protrusions growth during neuronal differentiation (related to Figure 1).

Graphs showing the change in length over time of the left (orange) and right (blue) basal protrusions from individual non-apical progenitors (NAPs) and individual differentiating neurons. The time has been normalised from (0), the moment in which differentiating neurons begin elongation of basal protrusions, to (1), when neurons initiate axon formation.

Figure S2

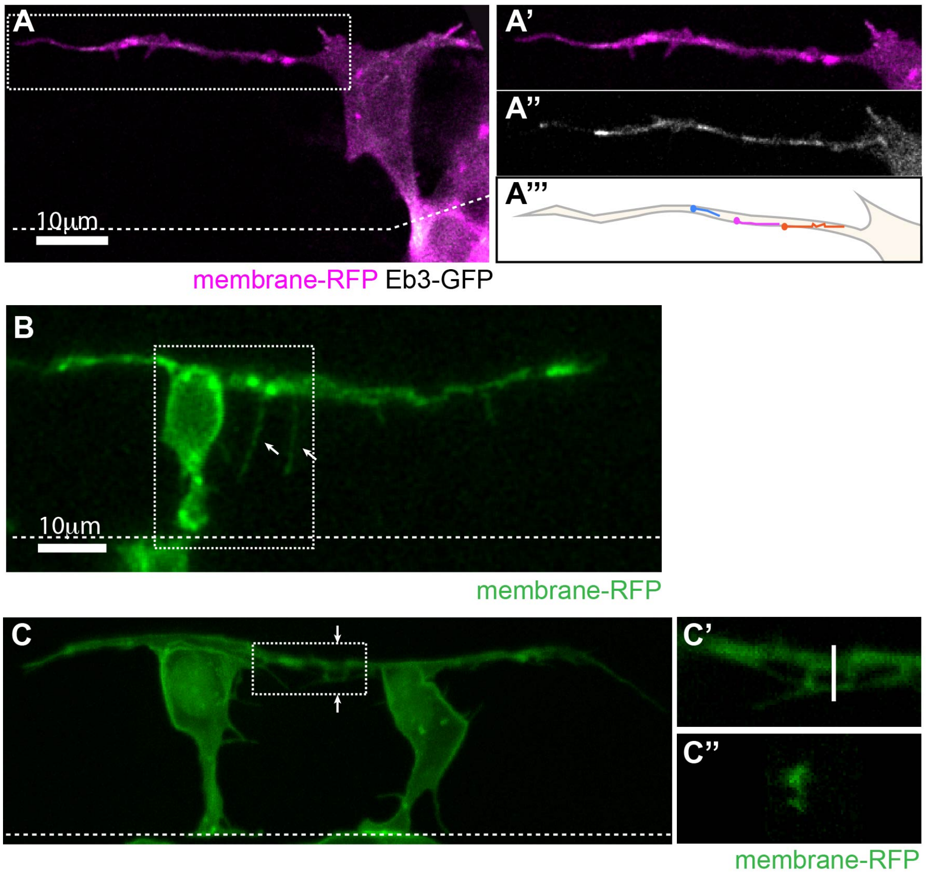

Basal protrusions contain dynamic microtubules, form filopodia and can overlap (related to Figure 1).

A) T-shaped differentiating neuron with a long basal protrusion. White box marks the area of higher magnification shown in A'-A'''). A') Membrane-RFP. A'') EB3-GFP (Movie 2). A''') Illustration of EB3-GFP comet trajectories during a 30 second time-lapse period.

B) T-shaped cell with filopodia (arrows) on basal protrusion (Movie 3).

C) Two neurons differentiating 18μm apart with overlapping basal arms. C') Higher magnification of white box in C). C'') Cross-section of the basal protrusions at the line shown in C').

All images are projected images from confocal z-stacks. Dashed line shows the apical surface.

Figure S3

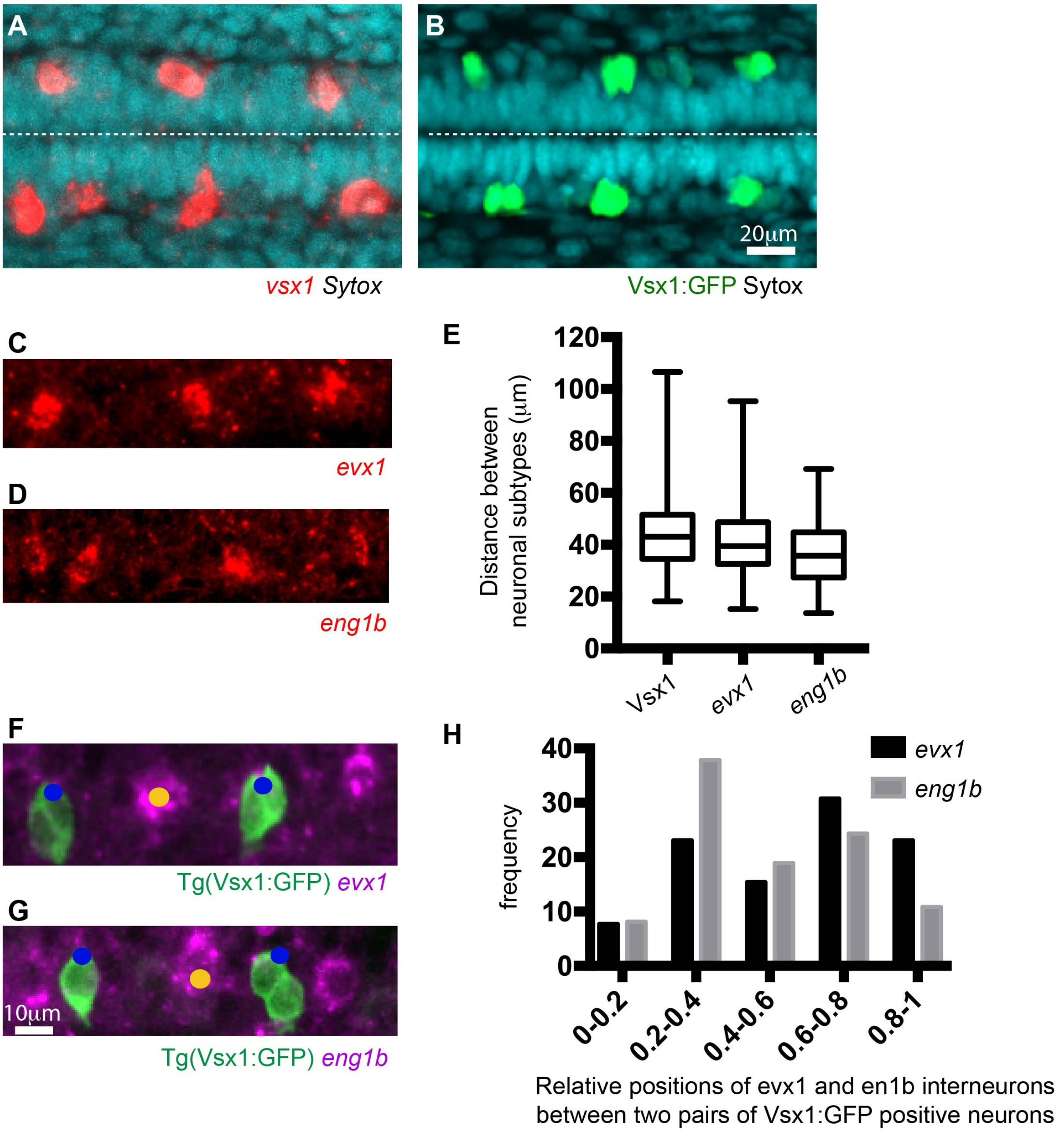

Neuronal spacing pattern in the zebrafish spinal cord (related to Figure 4).

A) and B) Dorsal views of Vsx1 expressing cells revealed by in situ hybridization (A) and Vsx1:GFP transgene expression (B) at 20 hfp. Dashed line shows the apical surface.

C) and D) Lateral views of evx1 (C) and eng1b (D) expressing spinal neurons revealed by in situ hybridization at 20hpf.

E) Box-and-whisker plot showing the distance between neurons of the same subtype in the most caudal spinal region at 20hpf (mean ± SD; Vsx1:GFP: 44.4 ± 14.1 μm,

n=18; evx1: 41 ± 15.4 μm, n=7; eng1b: 35.7 ± 13.3 μm, n=5 embryos). The line inside the box represents the median and whiskers represent minimum and maximum values. Data analysed using Kruskal-Wallis with Dunn's multiple comparison test (non-significant).

F and G) Dorsolateral views showing the relative positions of Vsx1:GFP and evx1 (F) or eng1b (G) expressing cells revealed by in situ hybridisation.

H) Frequency distribution chart shows the relative positions of evx1 and en1b expressing interneurons between two pairs of Vsx1:GFP expressing neurons. The distance between the two pairs of Vsx1:GFP neurons has been normalised from 0 (anterior) to 1 (posterior). All evx1 and eng1b expressing neurons that shared the same position with a Vsx1 neuron have been included in position (1).

Figure S4

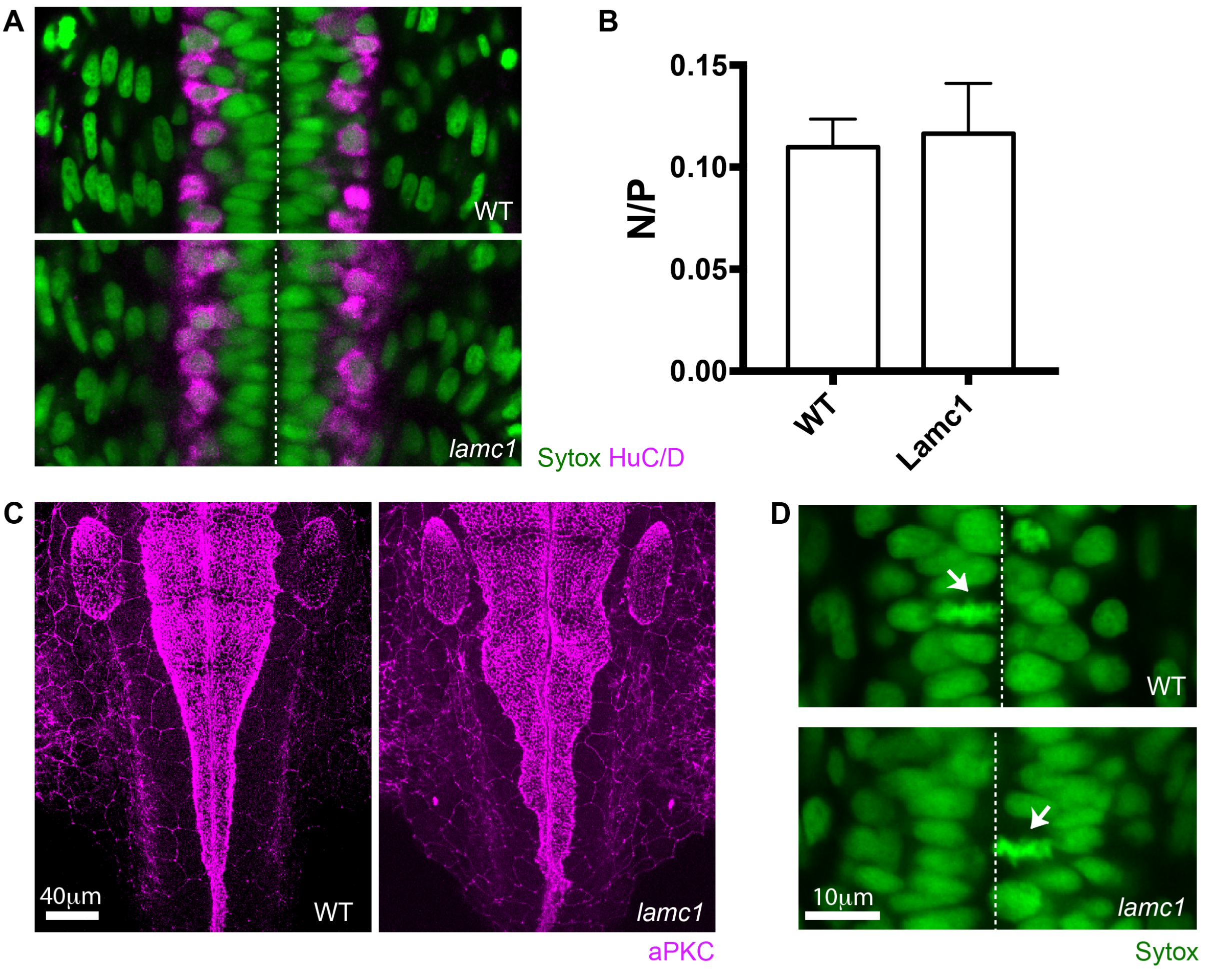

***Lamc1* mutant characterization (related to Figure 6).**

A) Dorsal view of spinal cord in wild type and *lamC1* mutant embryos. HuC/D immunoreactivity (magenta) reveals the position of spinal cord neurons at 24 hpf.

B) Graph showing the ratio of neurons to progenitors in wild type and *lamC1* mutant spinal cords at 24 hpf (mean ± SD, 0.11 ± 0.01 for wild type and 0.12 ± 0.02 for *lamc1* mutant, unpaired one-tailed t-test p=0.4428).

C) Dorsal view of wild type and *lamC1* mutant embryos at the hindbrain. aPKC immunoreactivity (magenta) shows the position of the apical surfaces at 28 hpf.

D) Dorsal view of spinal cord in wild type and *lamC1* mutant embryos showing apical mitoses (arrows).

Dashed line shows position of the apical surfaces in A) and D). Nuclei labelled by Sytox in A) and D).

Figure S5

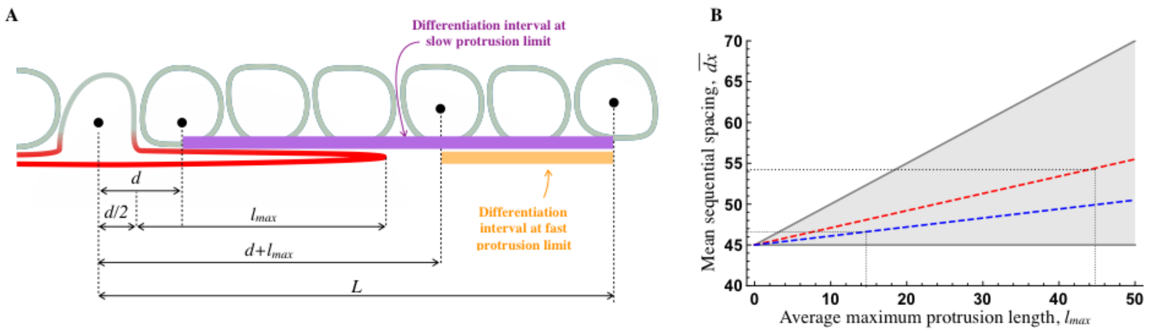

C

$\bar{dx} = \frac{L+d}{2} + 0.5\bar{l}_{max}$	Expected mean sequential spacing at fast protrusion limit.
$\bar{dx} = \frac{L+d}{2}$	Expected mean sequential spacing at slow protrusion limit.
$\bar{dx} = \frac{L+d}{2} + \phi\bar{l}_{max}, \quad 0 < \phi < 0.5$	Expected mean sequential spacing for protrusions observed experimentally.

The average distance between differentiation events depends linearly on the average maximum length reached by protrusions (related to Figure 7 and STAR methods section - Computational analysis of simulated and *in vivo* data).

A: The region where the next differentiation event is expected to occur following protrusion extension. Two limiting cases are illustrated: one where protrusion extension is slow and inefficient and so the subsequent differentiation event is expected to occur anywhere between distance *d* and *L* from our cell of interest. This leads to an expected mean for the sequential spacing, $\bar{dx}$, given by $\bar{dx} = \frac{L+d}{2}$. The second case illustrated represents instantaneous protrusion extension and immediate lateral inhibition. In this case the subsequent differentiation event is expected to occur anywhere between distance $d/2$ + l_{max} and L. This leads to an expected mean for the sequential spacing given by $\bar{dx} = \frac{L+d}{2} + 0.5\bar{l}_{max}$.

B: The expected relationship between the average distance between sequential events $\bar{dx}$ and the average maximum protrusion length l_{max} for the wild type (red)

and *lamc1* mutant (blue).

C: We expect the mean sequential spacing in a real tissue to relate to the protrusion maximum length by $\bar{dx} = \frac{L+d}{2} + \phi \bar{l}_{max}$ where $0 < \phi < 0.5$. The value of ϕ captures the speed and efficiency of protrusion extension and signaling. We computed ϕ in the wild type and *lamc1* mutant to be equal to $\phi_{WT} = 0.22$ and $\phi_{lamc1} = 0.024$. The decrease in the slope in the mutant is consistent with a reduced speed in protrusion extension, as observed experimentally. More details can be found under the Quantification and Statistical Analysis section in the STAR Methods.

Figure S6

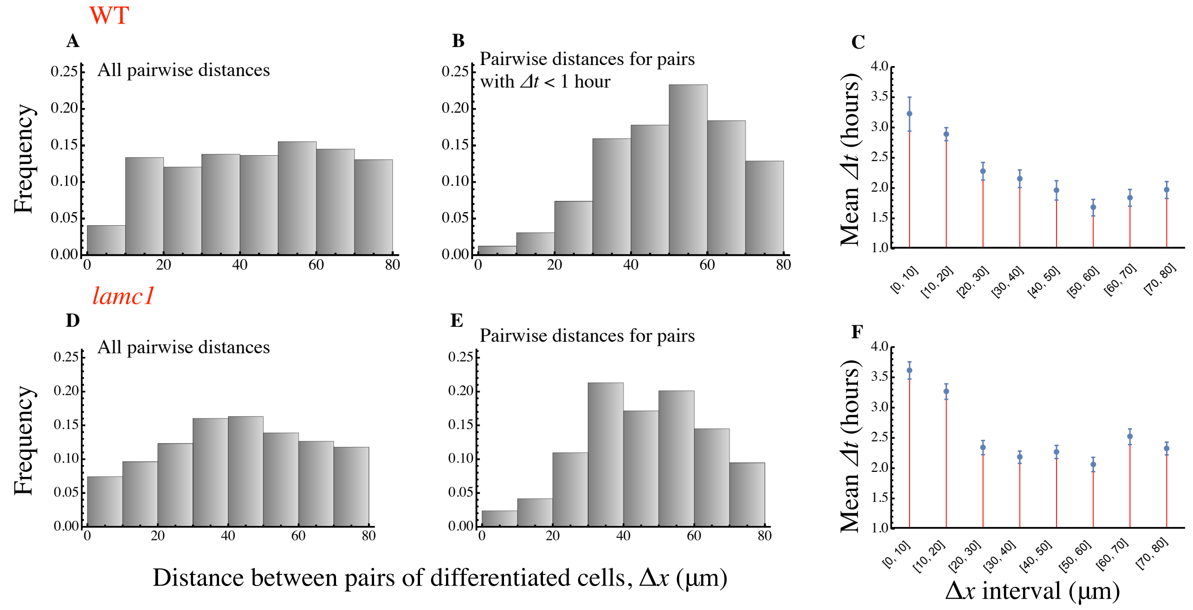

Neurons that are born closer in time tend to differentiate further apart in space (related to STAR methods section - Computational analysis of simulated and *in vivo* data).

Distribution of pairwise distances between all differentiating neurons (A for experimental wild type and D for *lamc1* mutant) and neurons that differentiated less than 1 hour apart from one another (B for experimental wild type and E for *lamc1* mutant).

C,F) show the mean pairwise time difference in the time of differentiation (Δt) for varying intervals of pairwise spatial differences Δx for the wild type (C) and *lamc1* mutant (F). All plots were produced using all measured pairs that satisfied x < 80μm.

Figure S7

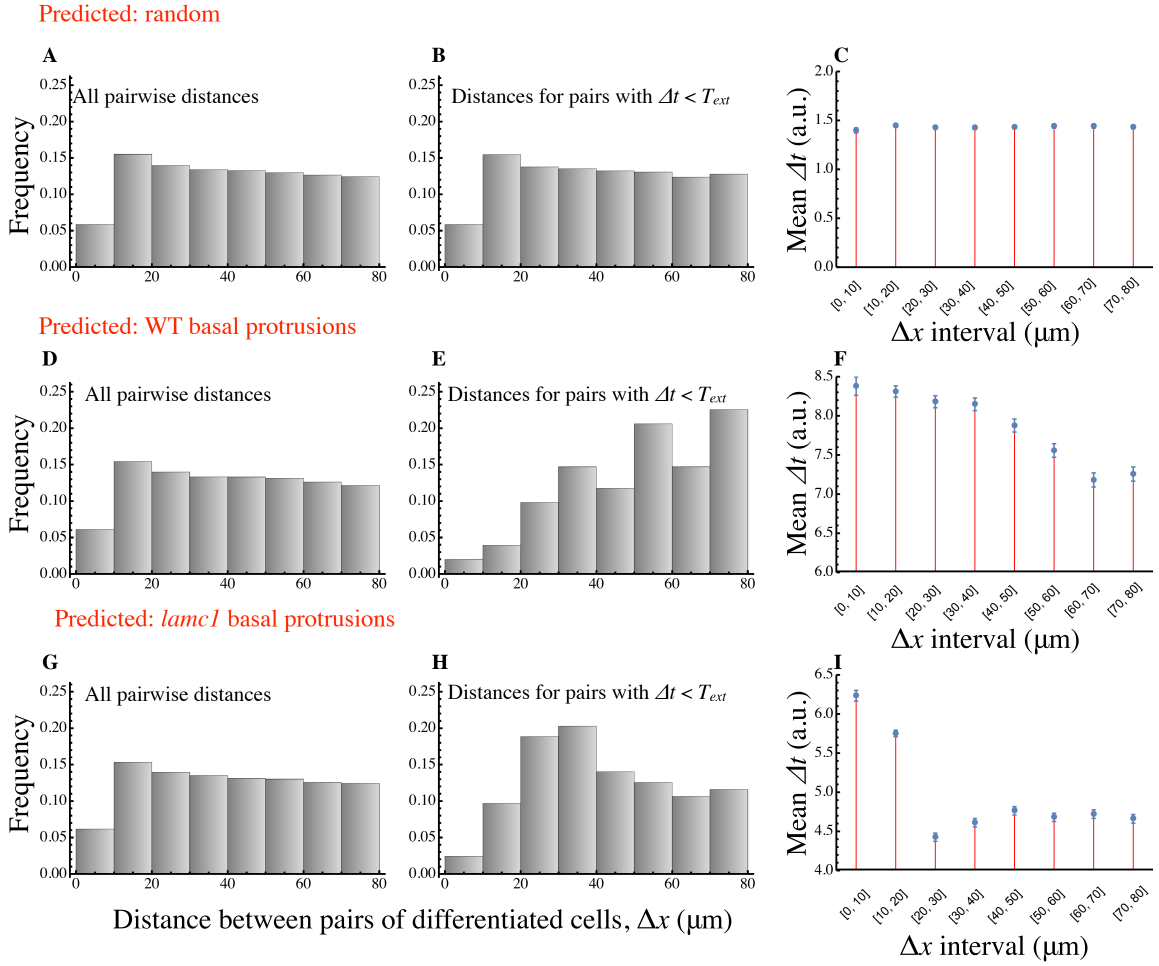

Theoretical model supports the role of basal protrusions in patterning spatiotemporal dynamics of neuron differentiation (related to STAR methods section - Computational analysis of simulated and *in vivo* data).

Distribution of pairwise distances between all differentiating neurons (A. for random differentiation, D. for wild type basal protrusions and G. for short basal protrusions) and neurons that differentiated less than Text time apart from one another in the simulations (B. for random differentiation, E. for wild type basal protrusions and H. for short basal protrusions).

C, F, I) show the mean pairwise time difference in the time of differentiation (Δt) for varying intervals of pairwise spatial differences Δx for a randomly differentiating tissue (C), a tissue where wild type basal protrusions mediate lateral inhibition (F) and a tissue where lamc1 like protrusions mediate lateral inhibition (I). Plots were produced using all simulated pairs that satisfied x < 80μm. Parameters used for the simulations are outlined in Table 1.

Figure S8

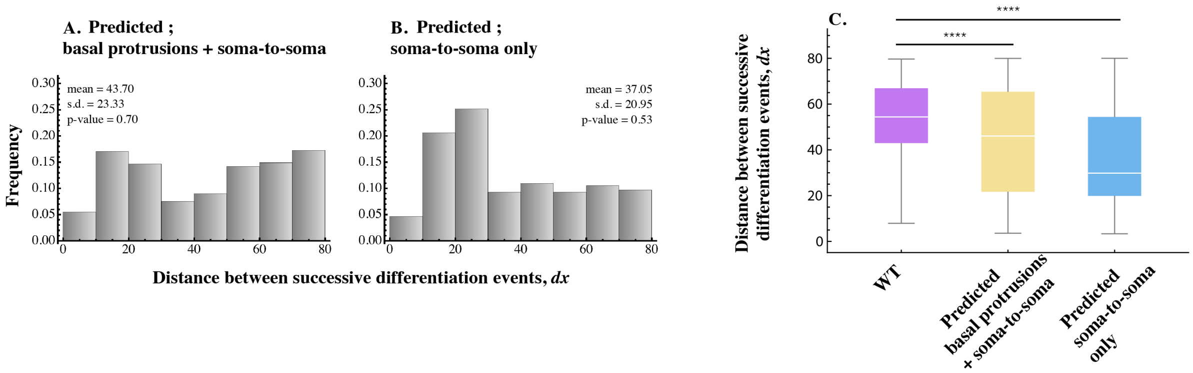

Soma-to-soma signaling does not explain experimentally observed distributions (related to STAR methods section - Computational analysis of simulated and *in vivo* data).

Predicted distributions of the distance between sequential events when wild type basal protrusions and soma-to-soma contacts mediate lateral inhibition (A) and when only soma-to-soma contacts mediate lateral inhibition (B). C) shows the box plots of the wild type data alongside the data shown in A) and B). The simulated distributions considering soma-to-soma signaling do not match experimental observations.

Kolmogorov-Smirnov test comparing wild-type and predicted distribution when protrusion and soma-to-soma signaling was assumed gave a p-value of 1.7 10^{-8}.

Kolmogorov-Smirnov test comparing wild-type and predicted distribution when only soma-to-soma signaling was assumed gave a p-value < 10^{-10}.

Supplemental data 1

Spatiotemporal pattern diagrams illustrating Vsx1:GFP neuronal differentiation events in time and space in the developing zebrafish spinal cord of wild type and *lamc1* mutant embryos.

WT emb BML102 a1 a

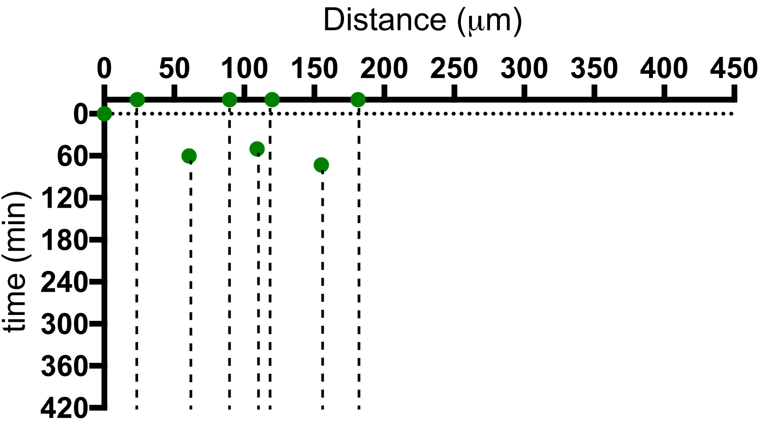

WT emb BML102 a1 b

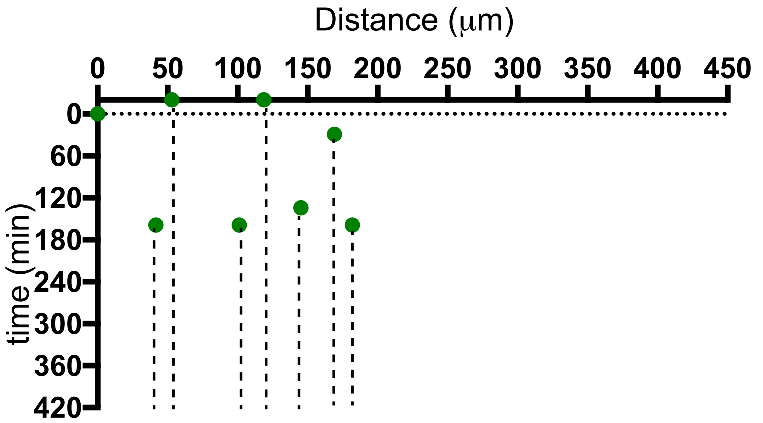

WT emb BML102 a2 b

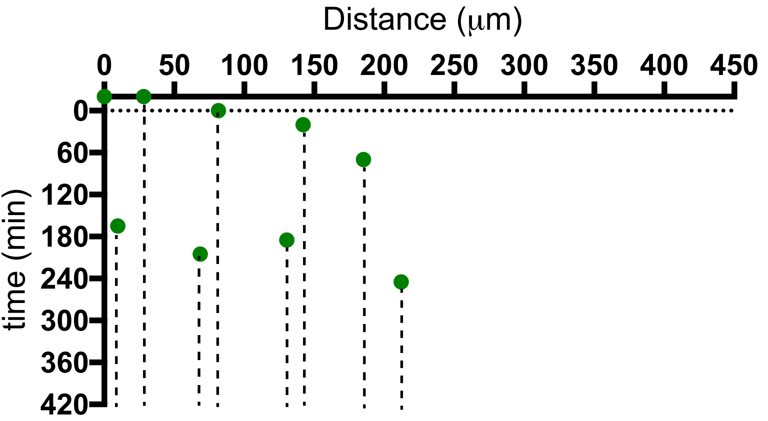

WT emb BML102 a2 a

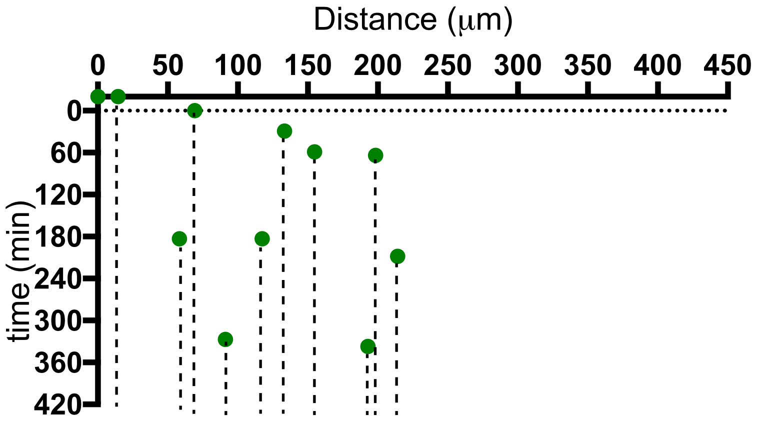

WT emb BML102 b2 a

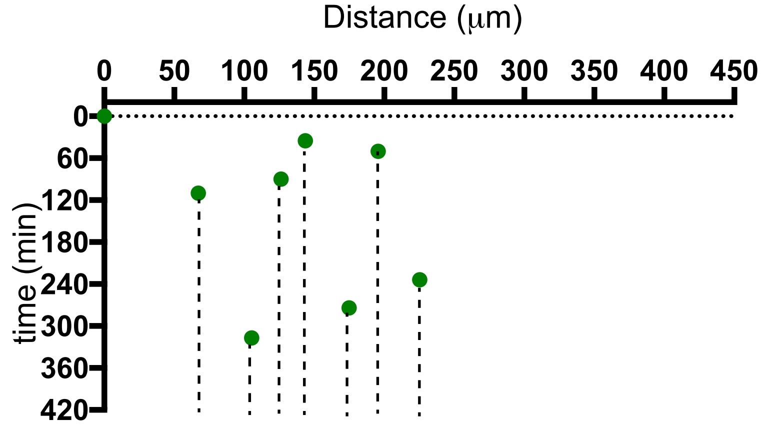

WT emb BML102 b2 b

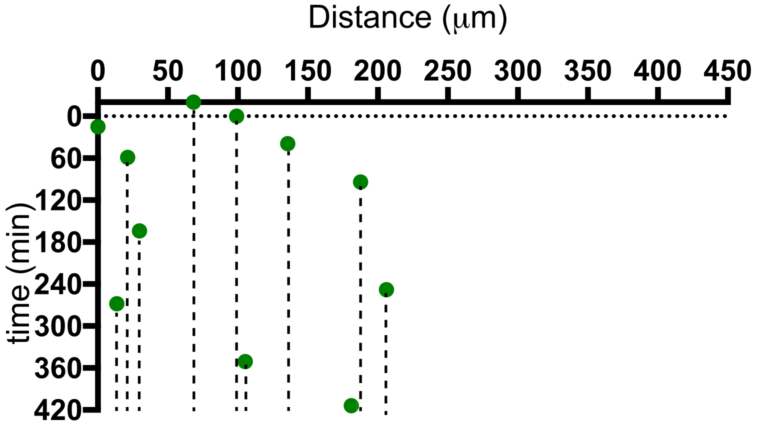

WT emb BML102 b1 a

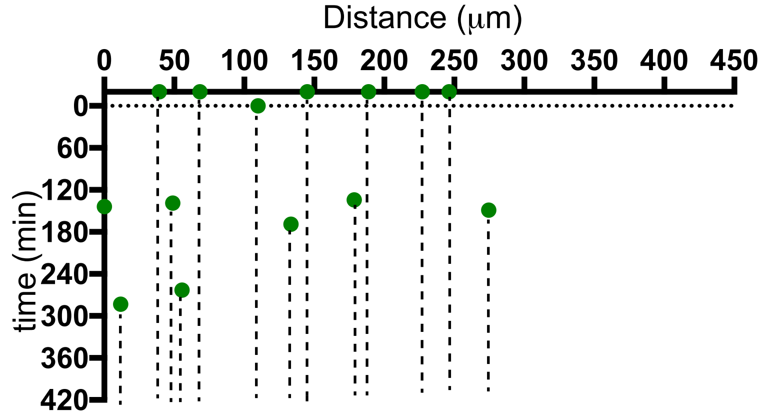

WT emb BML102 b1 b

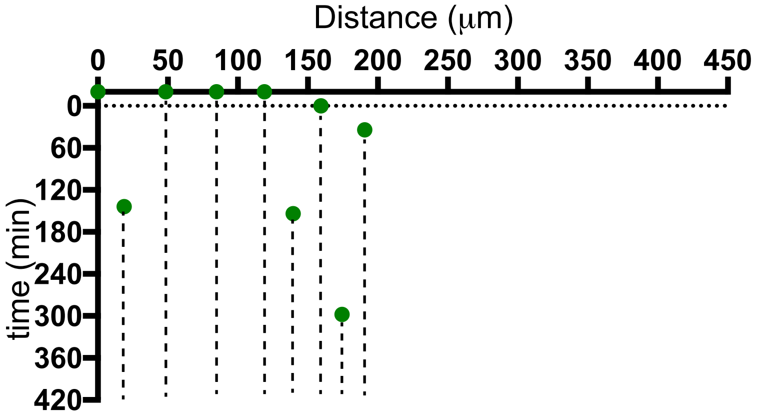

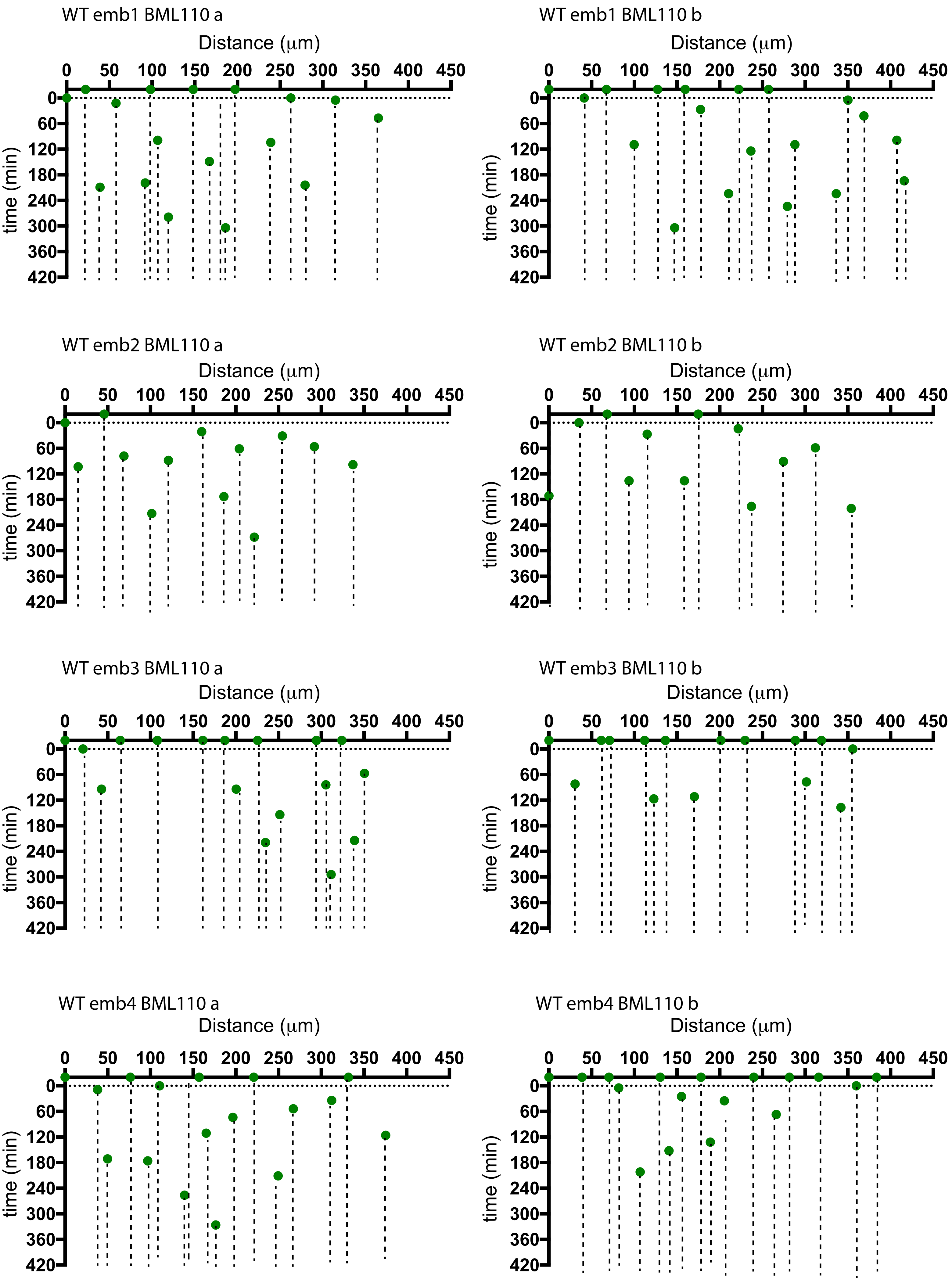
WT emb1 BML110 a
WT emb1 BML110 b
WT emb2 BML110 a
WT emb2 BML110 b
WT emb3 BML110 a
WT emb3 BML110 b
WT emb4 BML110 a
WT emb4 BML110 b
Distance (μm)
0 50 100 150 200 250 300 350 400 450
time (min)
0 60 120 180 240 300 360 420

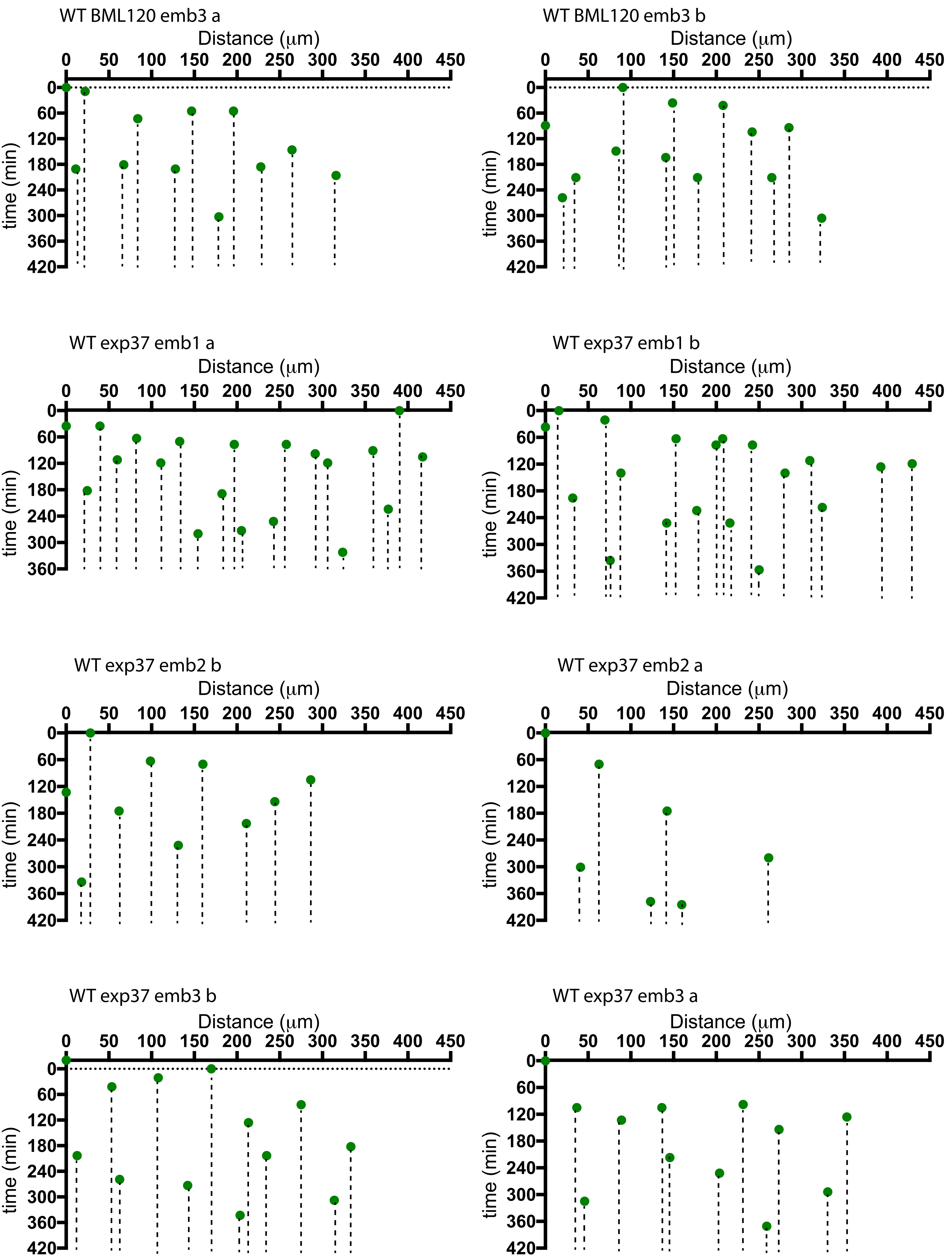
WT BML120 emb3 a
Distance (μm)
0 50 100 150 200 250 300 350 400 450
time (min)
0 60 120 180 240 300 360 420
WT BML120 emb3 b
Distance (μm)
0 50 100 150 200 250 300 350 400 450
time (min)
0 60 120 180 240 300 360 420
WT exp37 emb1 a
Distance (μm)
0 50 100 150 200 250 300 350 400 450
time (min)
0 60 120 180 240 300 360
WT exp37 emb1 b
Distance (μm)
0 50 100 150 200 250 300 350 400 450
time (min)
0 60 120 180 240 300 360 420
WT exp37 emb2 b
Distance (μm)
0 50 100 150 200 250 300 350 400 450
time (min)
0 60 120 180 240 300 360 420
WT exp37 emb2 a
Distance (μm)
0 50 100 150 200 250 300 350 400 450
time (min)
0 60 120 180 240 300 360 420
WT exp37 emb3 b
Distance (μm)
0 50 100 150 200 250 300 350 400 450
time (min)
0 60 120 180 240 300 360 420
WT exp37 emb3 a
Distance (μm)
0 50 100 150 200 250 300 350 400 450
time (min)
0 60 120 180 240 300 360 420

WT BML120 emb1 b

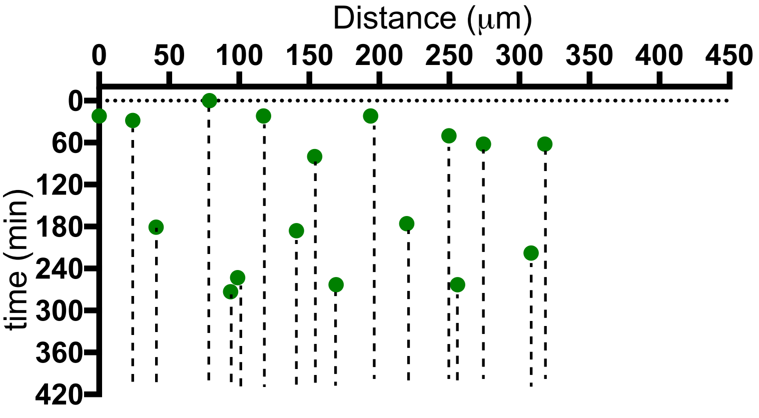

WT BML120 emb1 a

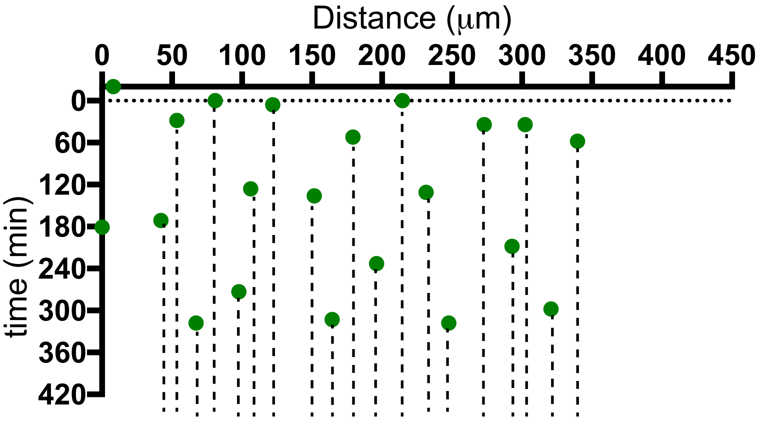

WT BML120 emb2 b

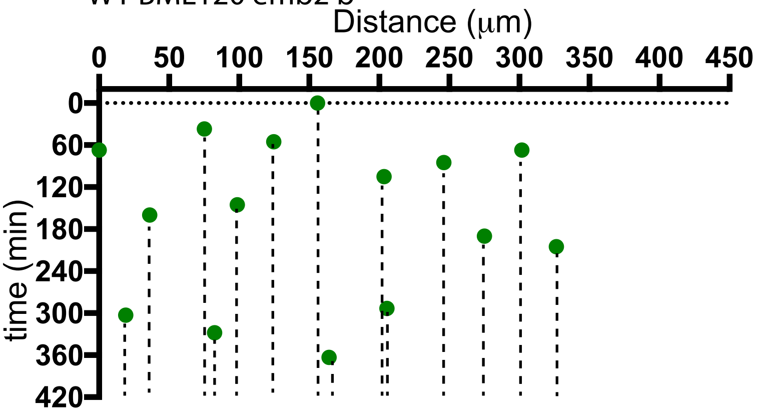

WT BML120 emb2 a

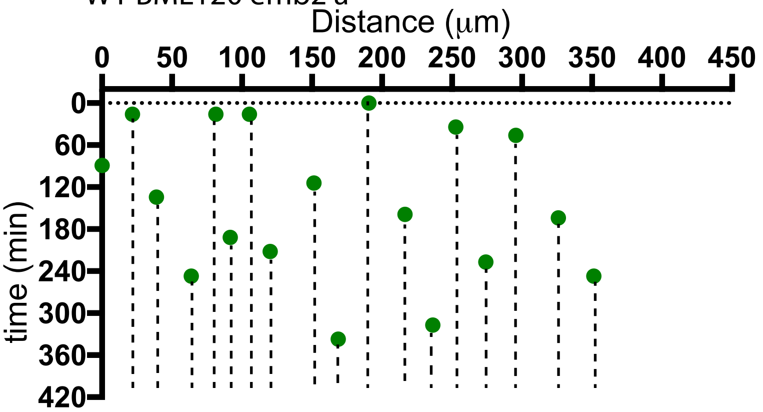

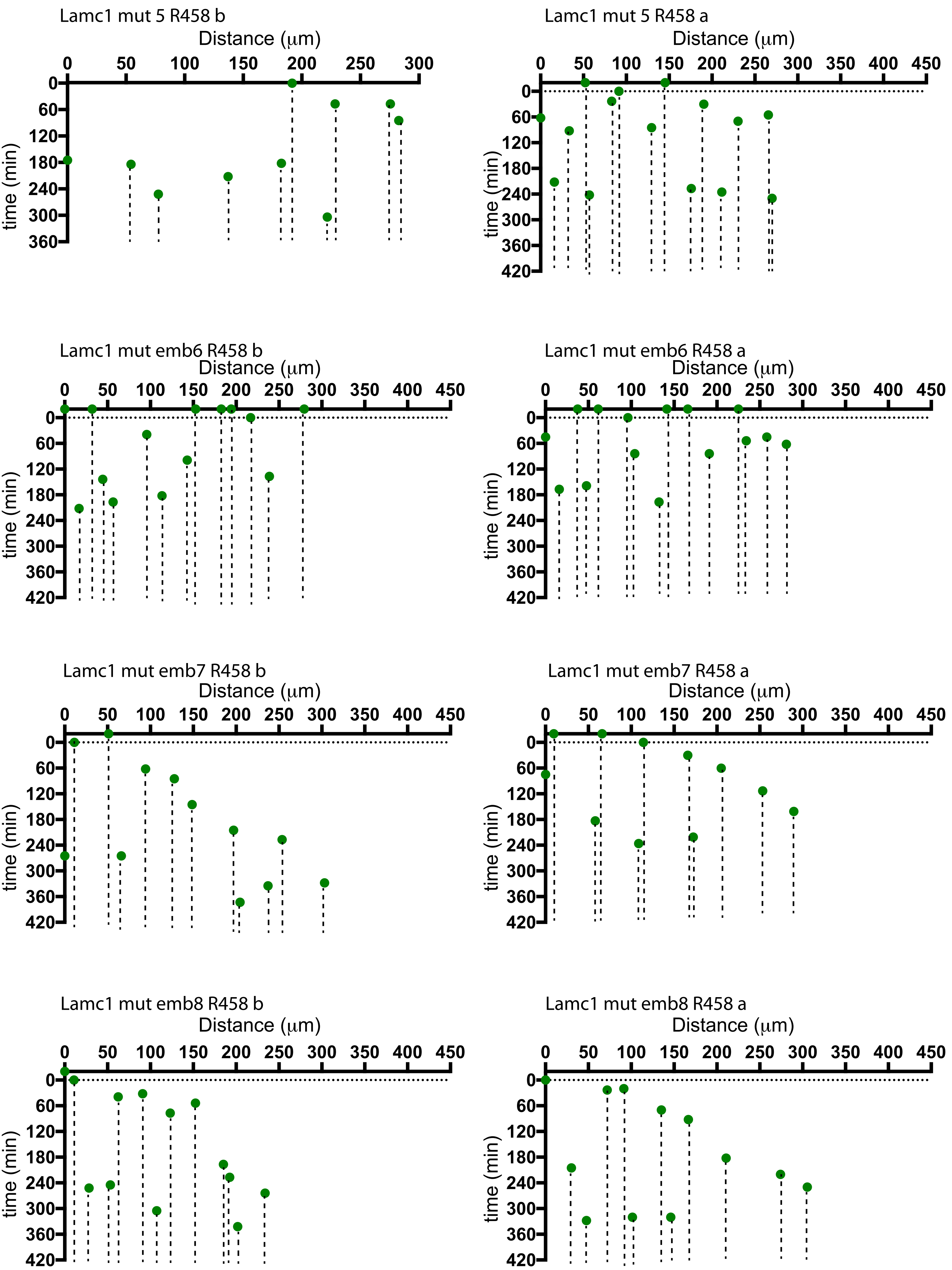
Lamc1 mut 5 R458 b
Distance (μm)
time (min)
Lamc1 mut 5 R458 a
Distance (μm)
time (min)
Lamc1 mut emb6 R458 b
Distance (μm)
time (min)
Lamc1 mut emb6 R458 a
Distance (μm)
time (min)
Lamc1 mut emb7 R458 b
Distance (μm)
time (min)
Lamc1 mut emb7 R458 a
Distance (μm)
time (min)
Lamc1 mut emb8 R458 b
Distance (μm)
time (min)
Lamc1 mut emb8 R458 a
Distance (μm)
time (min)

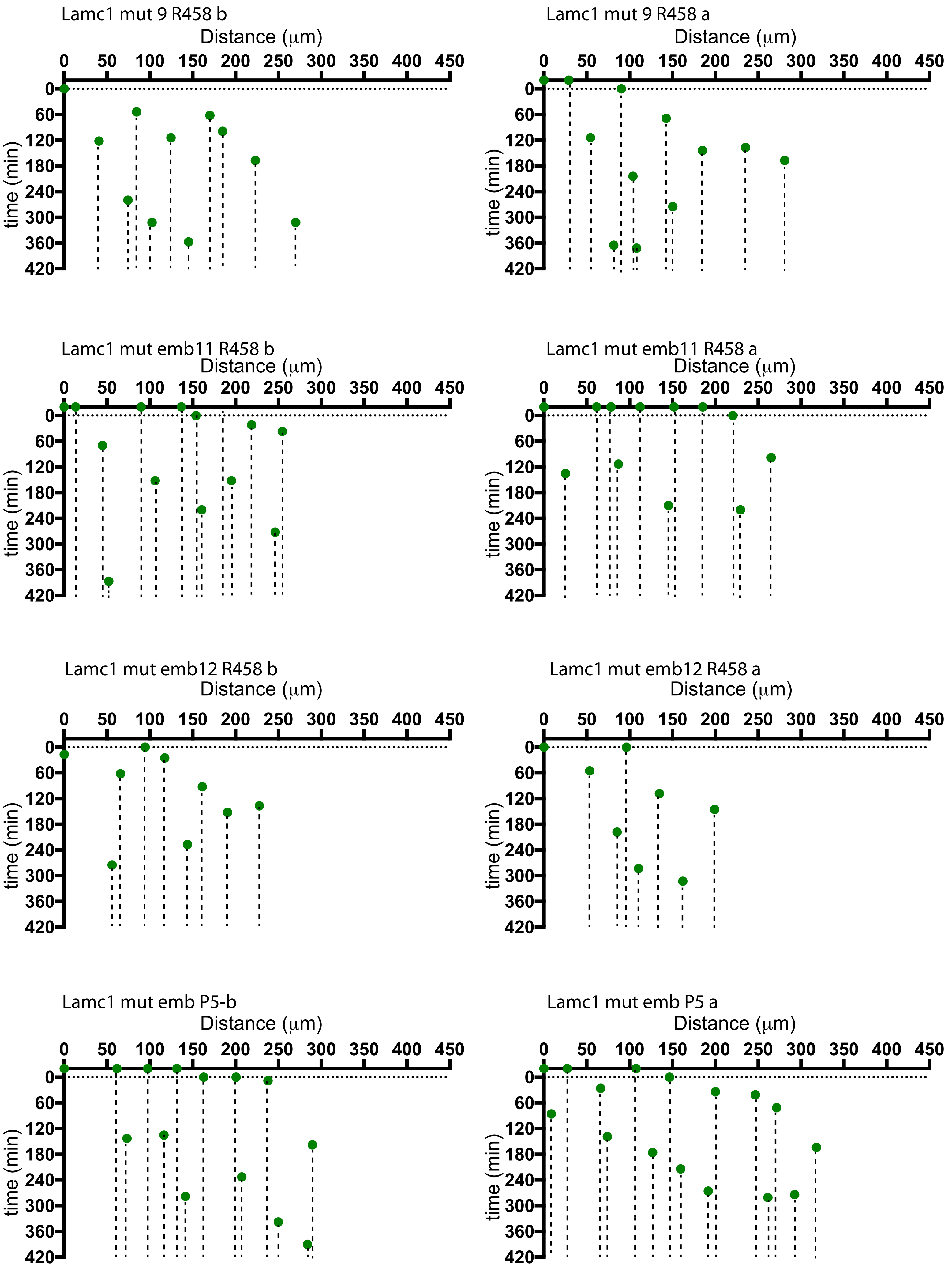
Lamc1 mut 9 R458 b
Distance (μm)
0 50 100 150 200 250 300 350 400 450
time (min)
0 60 120 180 240 300 360 420
Lamc1 mut 9 R458 a
Distance (μm)
time (min)
Lamc1 mut emb11 R458 b
Distance (μm)
time (min)
Lamc1 mut emb11 R458 a
Distance (μm)
time (min)
Lamc1 mut emb12 R458 b
Distance (μm)
time (min)
Lamc1 mut emb12 R458 a
Distance (μm)
time (min)
Lamc1 mut emb P5-b
Distance (μm)
time (min)
Lamc1 mut emb P5 a
Distance (μm)
time (min)

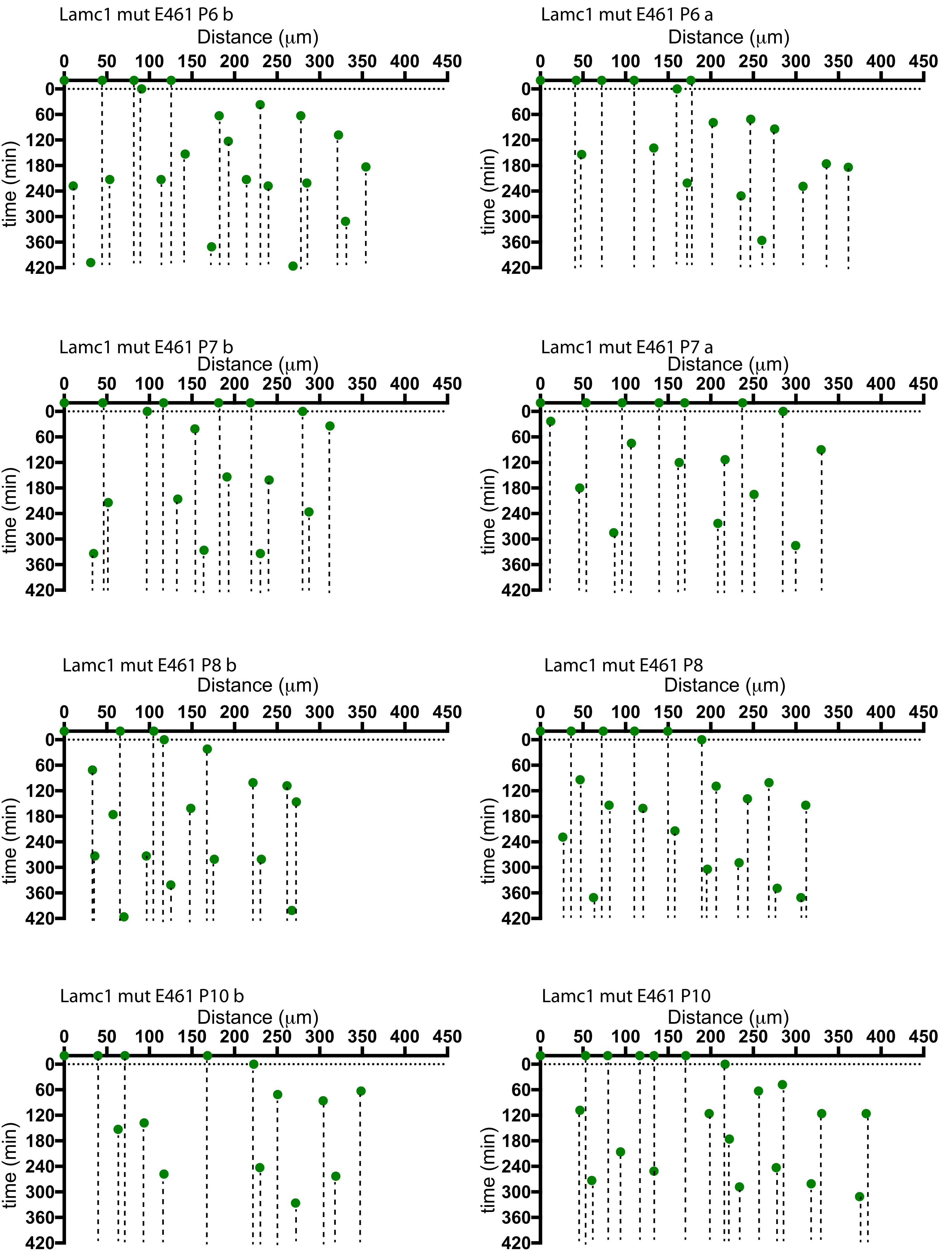
Lamc1 mut E461 P6 b
Lamc1 mut E461 P6 a
Lamc1 mut E461 P7 b
Lamc1 mut E461 P7 a
Lamc1 mut E461 P8 b
Lamc1 mut E461 P8
Lamc1 mut E461 P10 b
Lamc1 mut E461 P10
Distance (μm)
0 50 100 150 200 250 300 350 400 450
time (min)
0 60 120 180 240 300 360 420

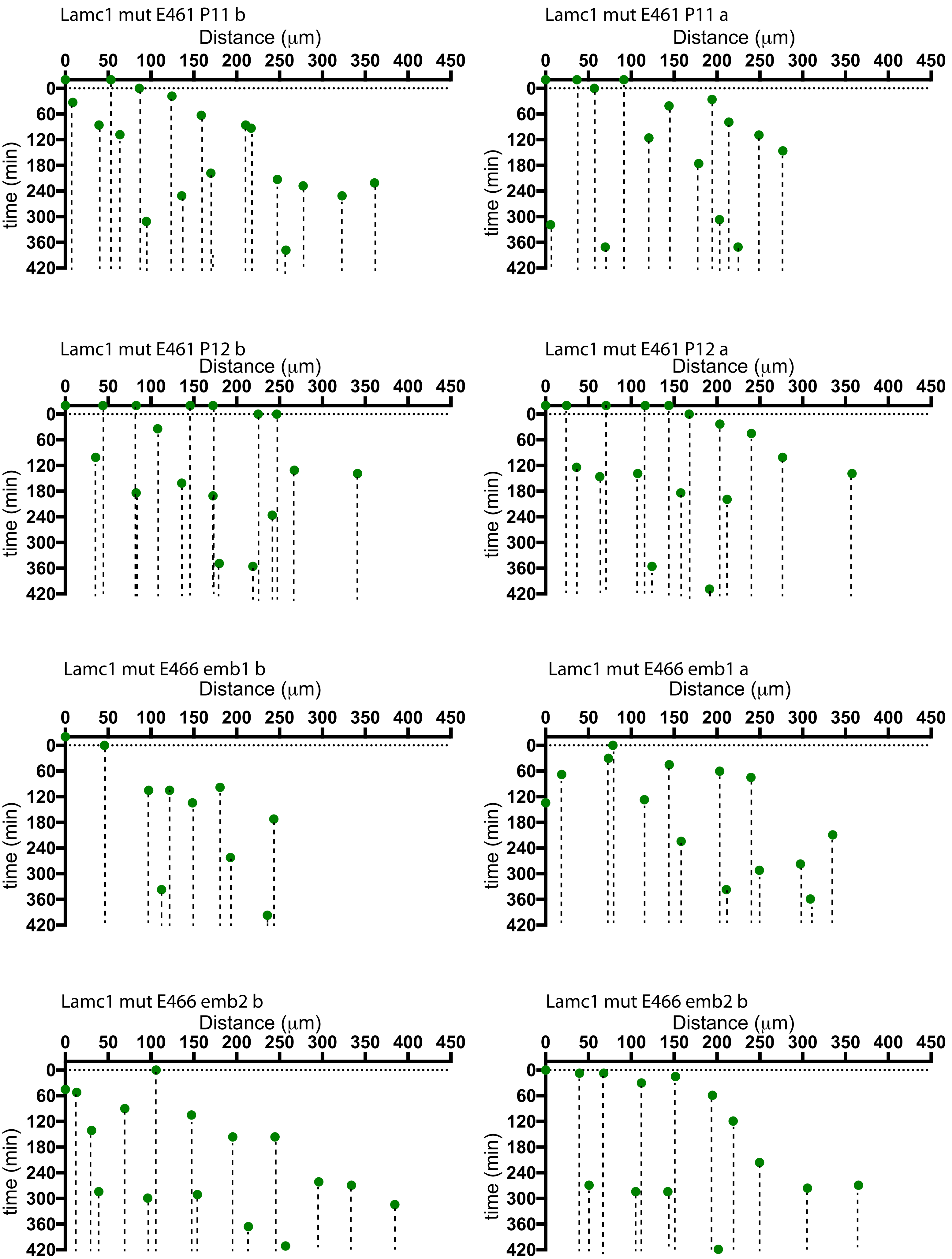
Lamc1 mut E461 P11 b
Lamc1 mut E461 P11 a
Lamc1 mut E461 P12 b
Lamc1 mut E461 P12 a
Lamc1 mut E466 emb1 b
Lamc1 mut E466 emb1 a
Lamc1 mut E466 emb2 b
Lamc1 mut E466 emb2 b
Distance (µm)
0 50 100 150 200 250 300 350 400 450
time (min)
0 60 120 180 240 300 360 420

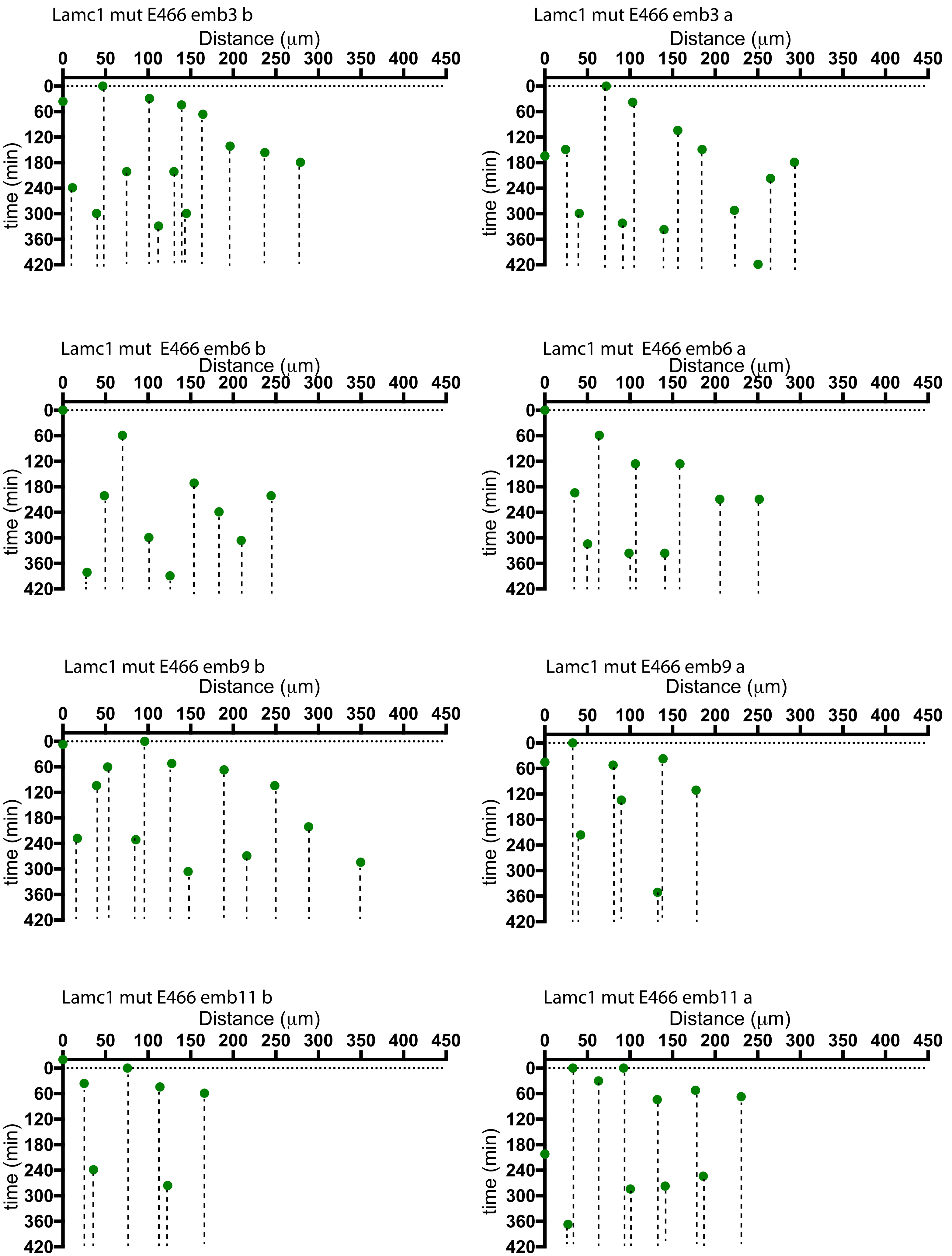
Lamc1 mut E466 emb3 b
Lamc1 mut E466 emb3 a
Lamc1 mut E466 emb6 b
Lamc1 mut E466 emb6 a
Lamc1 mut E466 emb9 b
Lamc1 mut E466 emb9 a
Lamc1 mut E466 emb11 b
Lamc1 mut E466 emb11 a
Distance (μm)
0 50 100 150 200 250 300 350 400 450
time (min)
0 60 120 180 240 300 360 420

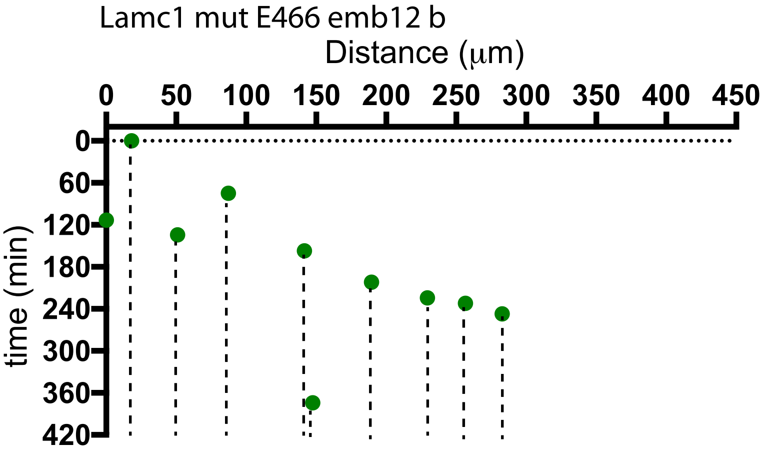
Lamc1 mut E466 emb12 b
Distance (μm)
0
50
100
150
200
250
300
350
400
450
time (min)
0
60
120
180
240
300
360
420

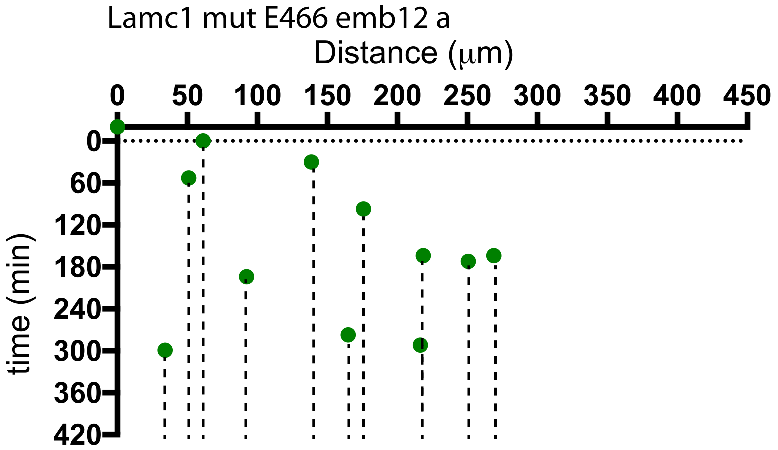
Lamc1 mut E466 emb12 a
Distance (μm)
0
50
100
150
200
250
300
350
400
450
time (min)
0
60
120
180
240
300
360
420
