# Supplementary material for: Basal Protrusions Mediate Spatiotemporal Patterns of Spinal Neuron Differentiation
Source: Dev Cell. 2019 Jun 17;49(6):907–919.e10. doi: 10.1016/j.devcel.2019.05.035 (PMC6584357; doi:10.1016/j.devcel.2019.05.035)
Supplement: Document S2. Article plus Supplemental Information [file mmc8.pdf]

Developmental Cell

Article

Basal Protrusions Mediate Spatiotemporal Patterns of Spinal Neuron Differentiation

Graphical Abstract

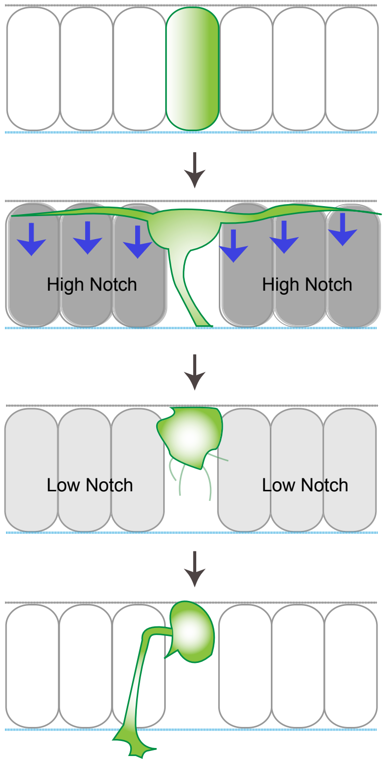

Highlights

- Embryonic spinal neurons elaborate longitudinal basal processes prior to axogenesis
- Basal processes reach several cell diameters and last a few hours before retraction
- Basal processes express high levels of Delta protein
- Experiments and theory show processes mediate transient long-range lateral inhibition

Authors

Zena Hadjivasiliou, Rachel E. Moore, Rebecca McIntosh, Gabriel L. Galea, Jonathan D.W. Clarke, Paula Alexandre

Correspondence

jon.clarke@kcl.ac.uk (J.D.W.C.), p.alexandre@ucl.ac.uk (P.A.)

In Brief

Lateral inhibition mediated by Delta-Notch signaling is thought to operate at short range between neighboring cells. Hadjivasiliou et al. show that transient protrusions up to 100 μm long from differentiating neurons extend the range of lateral inhibition and regulate spatiotemporal patterns of neuronal differentiation along the spinal cord.

Hadjivasiliou et al., 2019, Developmental Cell *49*, 907–919
June 17, 2019 © 2019 The Authors. Published by Elsevier Inc.
https://doi.org/10.1016/j.devcel.2019.05.035

Developmental Cell
Article

Basal Protrusions Mediate Spatiotemporal Patterns of Spinal Neuron Differentiation

Zena Hadjivasiliou,[1,2,5] Rachel E. Moore,[3,5] Rebecca McIntosh,[3,4] Gabriel L. Galea,[4] Jonathan D.W. Clarke,[3,6,*] and Paula Alexandre[4,6,7,*]
[1]Department of Biochemistry, Science II, University of Geneva, Geneva, Switzerland
[2]Centre for Mathematics and Physics in the Life Sciences and Experimental Biology, University College London, Gower Street, London WC1N 1EH, UK
[3]Centre for Developmental Neurobiology, Institute of Psychiatry, Psychology, and Neuroscience, King's College London, London SE1 1UL, UK
[4]Developmental Biology and Cancer, UCL GOS Institute of Child Health, London WC1N 1EH, UK
[5]These authors contributed equally
[6]Senior author
[7]Lead Contact
*Correspondence: jon.clarke@kcl.ac.uk (J.D.W.C.), p.alexandre@ucl.ac.uk (P.A.)
https://doi.org/10.1016/j.devcel.2019.05.035

SUMMARY

During early spinal cord development, neurons of particular subtypes differentiate with a sparse periodic pattern while later neurons differentiate in the intervening space to eventually produce continuous columns of similar neurons. The mechanisms that regulate this spatiotemporal pattern are unknown. *In vivo* imaging in zebrafish reveals that differentiating spinal neurons transiently extend two long protrusions along the basal surface of the spinal cord before axon initiation. These protrusions express Delta protein, consistent with the hypothesis they influence Notch signaling at a distance of several cell diameters. Experimental reduction of Laminin expression leads to smaller protrusions and shorter distances between differentiating neurons. The experimental data and a theoretical model support the proposal that neuronal differentiation pattern is regulated by transient basal protrusions that deliver temporally controlled lateral inhibition mediated at a distance. This work uncovers a stereotyped protrusive activity of newborn neurons that organize long-distance spatiotemporal patterning of differentiation.

INTRODUCTION

During the early stages of vertebrate neurogenesis, neurons of particular subtypes initially differentiate along the spinal cord with a sparse periodic pattern but eventually produce more continuous columns of similar neurons (Figure 1A; Dale et al., 1987; Roberts et al., 1987; Higashijima et al., 2004a, 2004b; Kimura et al., 2006; Batista et al., 2008; England et al., 2011). The mechanisms that regulate this pattern of differentiation are unknown. Delta-Notch-mediated lateral inhibition is a regulator of vertebrate neurogenesis (Chitnis et al., 1995; Henrique et al., 1997; Appel et al., 2001; Okigawa et al., 2014), but this conventionally operates in a juxtacrine fashion between Delta-expressing cells and their immediate neighbors and cannot explain the spatial and temporal pattern of neuronal differentiation along the embryo spinal cord.

Recent evidence, however, suggests the distance over which contact mediated signaling of various types can operate can be extended by cellular protrusions capable of spanning several cell diameters (reviewed in Buszczak et al., 2016; Pröls et al., 2016). For example, signaling through long cellular protrusions plays a role during limb patterning in the chick embryo (Sanders et al., 2013), in the development of zebrafish pigmentation stripes (Eom et al., 2015), and in neural plate patterning in the zebrafish (Stanganello et al., 2015). In fact, dynamic cellular protrusions from the basal surface of sensory organ precursor (SOP) cells have been proposed to mediate long-distance lateral inhibition to regulate the sparse distribution of mechanosensory bristles in the fly notum and wing disk (De Joussineau et al., 2003; Cohen et al., 2010; Hadjivasiliou et al., 2016; Hunter et al., 2016, 2019). Whether similar protrusive activity mediates long-distance spacing patterns in the vertebrate central nervous system (CNS) is not known, but long and short cellular protrusions expressing the Notch ligand Delta-like 1 have been described on intermediate progenitors in the embryonic mammalian cortex (Nelson et al., 2013). Furthermore, dynamic protrusive activity on the surface of recently born spinal neurons can be observed in slice cultures of chick embryo spinal cord (Das and Storey, 2014).

To determine whether cellular protrusions could also play a role in the patterning of spinal neuronal differentiation, we addressed these issues in the zebrafish embryo spinal cord. Live *in vivo* imaging revealed all spinal neurons transiently extend two long cellular protrusions along the basal surface of the spinal cord prior to axon initiation and apical detachment. We show these long basal protrusions express Delta protein at high level and Notch reporter activation is upregulated in cells in their vicinity. Furthermore, experimental reduction of the basal protrusion length results in reduced spacing between differentiating neurons. Our *in vivo* data are supported by a theoretical model, whose output is consistent with the hypothesis that neuronal differentiation is regulated by lateral inhibition mediated at a

This is an open access article under the CC BY license (http://creativecommons.org/licenses/by/4.0/).

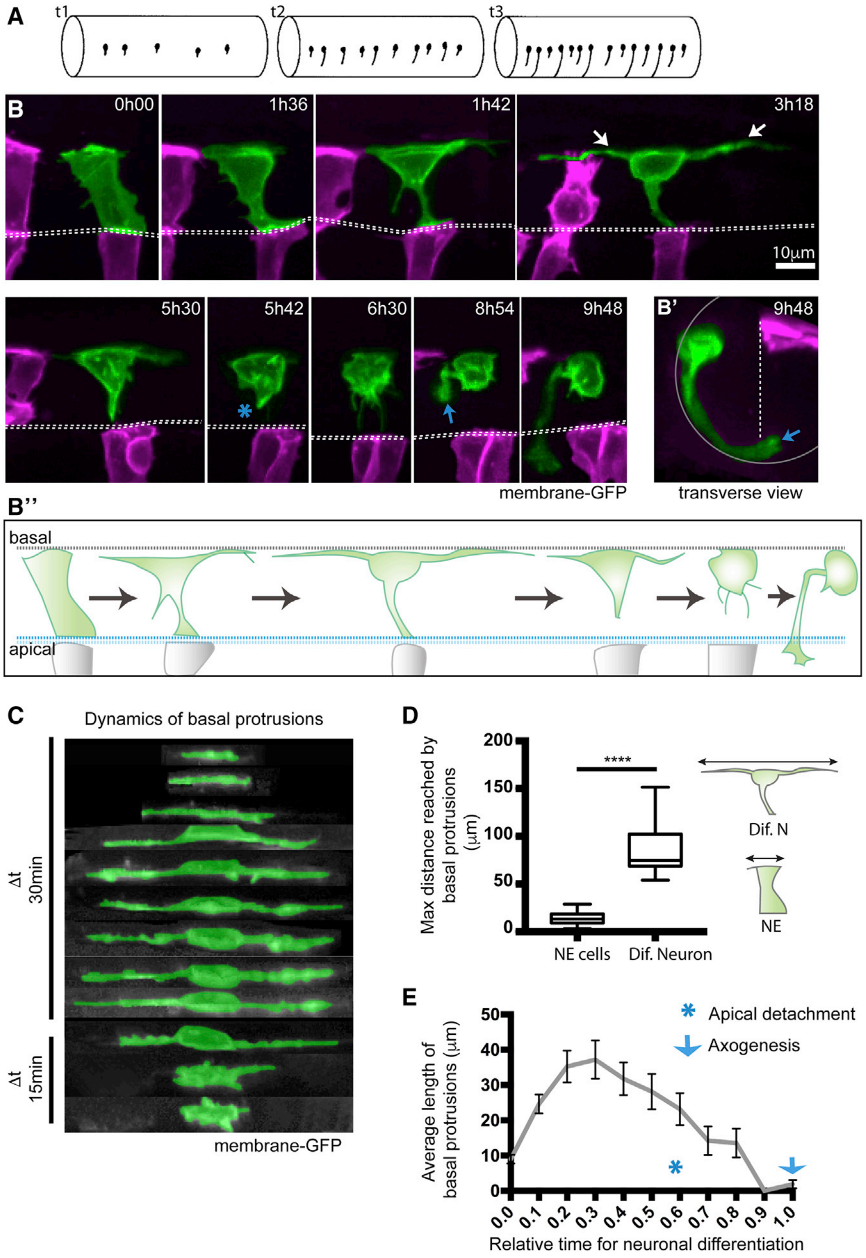

Figure 1. Differentiating Spinal Neurons Transiently Elongate Two Long Basal Protrusions along the A/P Axis before Detaching from the Apical Surface

(A) Diagram to show spinal neurons differentiate with an initial long-distance spacing pattern (t_1). Later differentiating neurons of the same type subsequently fill in the gaps between the earlier differentiated cells (t_2 and t_3) to generate a near continuous column of neurons. Lateral view of spinal cord, dorsal to top.

(B) Image sequence from confocal time lapse from dorsal view illustrates the early steps in neuronal differentiation that precede axogenesis in the spinal cord. A differentiating neuron (green) transiently adopts a T shape through the maintenance of an apical attachment and the elongation of two long cellular protrusions at the basal surface of the neuroepithelium (arrowed in time point 3h18). Following the retraction of basal protrusions, the apical process detaches (blue asterisk in timepoint 5h42). The axon is formed (blue arrow in 8h54) and grows ventrally and across the midline (see Video S1). Images are maximum projections from confocal z stacks.

(B′) Transverse reconstruction of B at 9h48. Cells visualized with membrane-GFP, with non-neuronal cells artificially colored in magenta. Dashed line shows position of the apical surfaces.

(B″) Diagram summarizes the steps involved in neuronal differentiation: transient formation of basal protrusions followed by their retraction, apical detachment and axonal growth. Apical and basal surfaces of the neuroepithelium are outlined by a blue (bottom) and gray (top) dashed line, respectively.

(C) Kymographic representation of extension and retraction of basal protrusions of a differentiating neuron (green) viewed laterally.

(D) Box-and-whisker plot showing maximal basal extension of differentiating neurons (mean ± SD, 86.8 ± 25.3 μm, n = 21 cells) and non-differentiating neuroepithelial cells (mean ± SD, 14.3 ± 6.2 μm, n = 74 cells). The line inside the box represents the median and whiskers represent minimum and maximum values. Data analyzed using unpaired one-tail Mann-Whitney test (p-value < 0.0001).

(E) Average length of individual basal protrusions during neuronal differentiation (n = 13 cells). The time has been normalized from (0), the moment in which differentiating neurons begin elongation of basal protrusions, to (1), when neurons initiate axon formation. Error bars indicate SEM.

distance by transient basal protrusions. Our work thus reveals a stereotyped protrusive activity of differentiating neurons that organizes long-distance spatiotemporal patterning of neuronal differentiation in the embryo spinal cord.

RESULTS

Differentiating Spinal Neurons Transiently Elongate Two Long Basal Protrusions along the A/P Axis before Detaching from the Apical Surface

To study the early phases of neuronal differentiation *in vivo*, we labeled small numbers of cells in the zebrafish embryo spinal cord by mosaic expression of membrane-GFP and captured their behavior with confocal time-lapse microscopy from 18 to 42 h post fertilization (hpf). Analysis of more than 100 cells that differentiate into neurons reveals a stereotyped, transient T-shaped transition from a cell that is attached to the apical surface of the neuroepithelium to a basally positioned neuron with the beginnings of a single axon extension. This transition involves the elongation of two longitudinally directed cellular processes that protrude along the basal surface of the neural tube, one protruding anteriorly and the other posteriorly (Figure 1B, time point 1h42 and 3h18; Figures 1B″ and 1C; Video S1). These basal protrusions can be asymmetric in length (17 out of

28 cells) and each protrusion can reach up to 109 μm (mean ± SD, 42.6 ± 20.2 μm, n = 24 cells) with a combined length of up to 151.5 μm (mean ± SD, 86.8 ± 25.3 μm, n = 21 cells) (Figures 1D and S1). The basal protrusions are typically present on differentiating neurons for several hours (mean ± SD, 6.8 ± 2.2 h, n = 13 cells) and grow on average 6× longer than the basal extensions formed by the non-differentiating neural progenitors (mean ± SD, 14.3 ± 6.2 μm, n = 74 cells) (Figure 1D). After reaching their maximum length, basal protrusions begin to retract back to the cell body, and this is followed by the detachment and retraction of the apical process (19 out of 24 cells) (Figure 1B, from time point 3h18 to 5h42; Figure 1E). In a few cases (5 out of 24 cells) the apical detachment preceded the retraction of basal protrusions. Although apical and basal process retraction occurs at roughly the same time they do not appear to be strictly synchronized, suggesting they may be independent of one another. After these three processes have retracted, cells adopt a near spherical shape and the cell body becomes highly enriched in filopodial activity that diminishes prior to axon formation (23 out of 27 cells) (Figure 1B, time point 6h30 and 8h54; Figure 1B′; Video S1). The transient basal protrusions contain dynamic microtubules (Figure S2A) and often produce filopodia that are directed radially toward the apical surface (Figure S2B). Basal protrusions from nearby differentiating cells can overlap (Figures S2C–S2C″).

Differentiating spinal neurons thus stereotypically adopt a transient T shape prior to apical detachment and axon formation (summarized in Figure 1B″). These observations reveal a new *in vivo* cellular behavior that precedes axogenesis and distinguishes the neuronal precursors in the process of differentiation from surrounding neural progenitors.

Stereotyped Axon Formation Follows Basal Protrusion Retraction

Studies of neuronal differentiation *in vitro* have revealed that axons derive by selection and specialization of one neurite from several pre-existing neurites (Dotti et al., 1988; Craig and Banker, 1994; Barnes, and Polleux, 2009). To investigate whether the axons of spinal neurons *in vivo* might derive from the transient long basal protrusions, we monitored axon initiation. Neurons were located at many different dorsoventral (D/V) levels of spinal cord and thus likely represent many different subtypes of spinal projection neuron. Our 3D reconstruction analyses revealed that axonal outgrowth almost always follows the full retraction of basal protrusions (27 out of 31 cells) (Figures 2A and 2B; Videos S1 and S2), and in contrast to *in vitro* observations, axons never differentiated from an existing cellular protrusion. The majority of subtypes of spinal neurons have an axon that runs ventrally and circumferentially from the cell body before either crossing the ventral floor plate or turning anteriorly or posteriorly to join the ipsilateral longitudinal axon tracts (Bernhardt et al., 1990). Our observations show that this ventral circumferential axon trajectory is initiated stereotypically at the outset of axon growth, directly from the cell body and is spatially independent of and perpendicular to the preceding transient basal protrusions (Figures 1B, 2A, and 2B; Videos S1 and S2). In only one case have we seen a neuron generate what appears to be a forked axon with two ventrally directed branches. In this case, one of these branches was quickly retracted leaving the usual morphology of a single ventral axon.

Our analysis does not include the primary sensory Rohon-Beard neurons, which develop three axons (two central longitudinal axons and a peripheral axon) and are likely to use a different program of axogenesis (Andersen and Halloran, 2012). Our data also contain only one definitive motoneuron because their very ventral location impedes imaging. However, the single motoneuron has short basal protrusions and was the only neuron that did not have a ventral trajectory to its initial axon growth; instead, it directed its axon laterally from the cell body toward the nearby somite boundary before exiting the cord to innervate the muscles (Figure 2C, observations summarized in diagram in Figure 2D).

Non-apical Progenitors in Spinal Cord Also Extend Basal Protrusions prior to Apical Detachment

In addition to the apical progenitors that generate most of the neurons of zebrafish CNS, a scarce population of basal progenitors that divide in non-apical locations is also present (Alexandre et al., 2010; McIntosh et al., 2017). We call these progenitors non-apical progenitors (or NAPs) and previously demonstrated that the majority of spinal NAPs express Vsx1 and share molecular and regulatory mechanisms with neurons (McIntosh et al., 2017). This prompted us to investigate whether spinal NAPs might also share the morphological program of differentiation with neurons. We were able to monitor 7 NAPs by confocal time-lapse microscopy all of which undergo the stereotypical T-shape transition characteristic of differentiating neurons prior to their basal mitosis (Figure 3A; Video S3). The NAP exemplified in Figure 3A has a basal cell body that transiently extends a pair of long basal protrusions that are filopodia rich while still attached to the apical surface (Figure 3A; Video S3). The basal protrusions on NAPs are often asymmetric in length (6 out of 7 cases). On some cells, basal protrusions do not fully retract before NAP mitoses (4 out of 7 cells) (Figures 3A and 3B; Video S3). In these cases, the retraction of basal protrusions is completed after mitosis (green arrow in Figures 3A and 3B) but still prior to axon formation in the two daughter neurons (blue arrow in Figures 3A and 3B; Video S3).

These observations show spinal neurons and NAPs share common stereotypical morphological behaviors and further confirm that spinal Vsx1 NAPs and differentiating neurons share cellular and molecular characteristics as suggested previously (McIntosh et al, 2017).

Differentiating Telencephalic Neurons Do Not Form Long Transient Basal Protrusions

The elongation of basal protrusions seems to be a consistent feature of differentiating neurons and NAPs in the zebrafish spinal cord. To investigate whether the T-shape transition is common to differentiating neurons in other regions of the zebrafish CNS, we analyzed neuronal differentiation in the dorsal telencephalon from 20 to 40 hpf. Using this approach, we find that differentiating neurons in the telencephalon do not extend transient basal protrusions prior to apical detachment and axogenesis (n = 16 out of 16 cells) (Figure 3C; Video S4). In these cells, axon formation derives from the basal end of the new neuron's

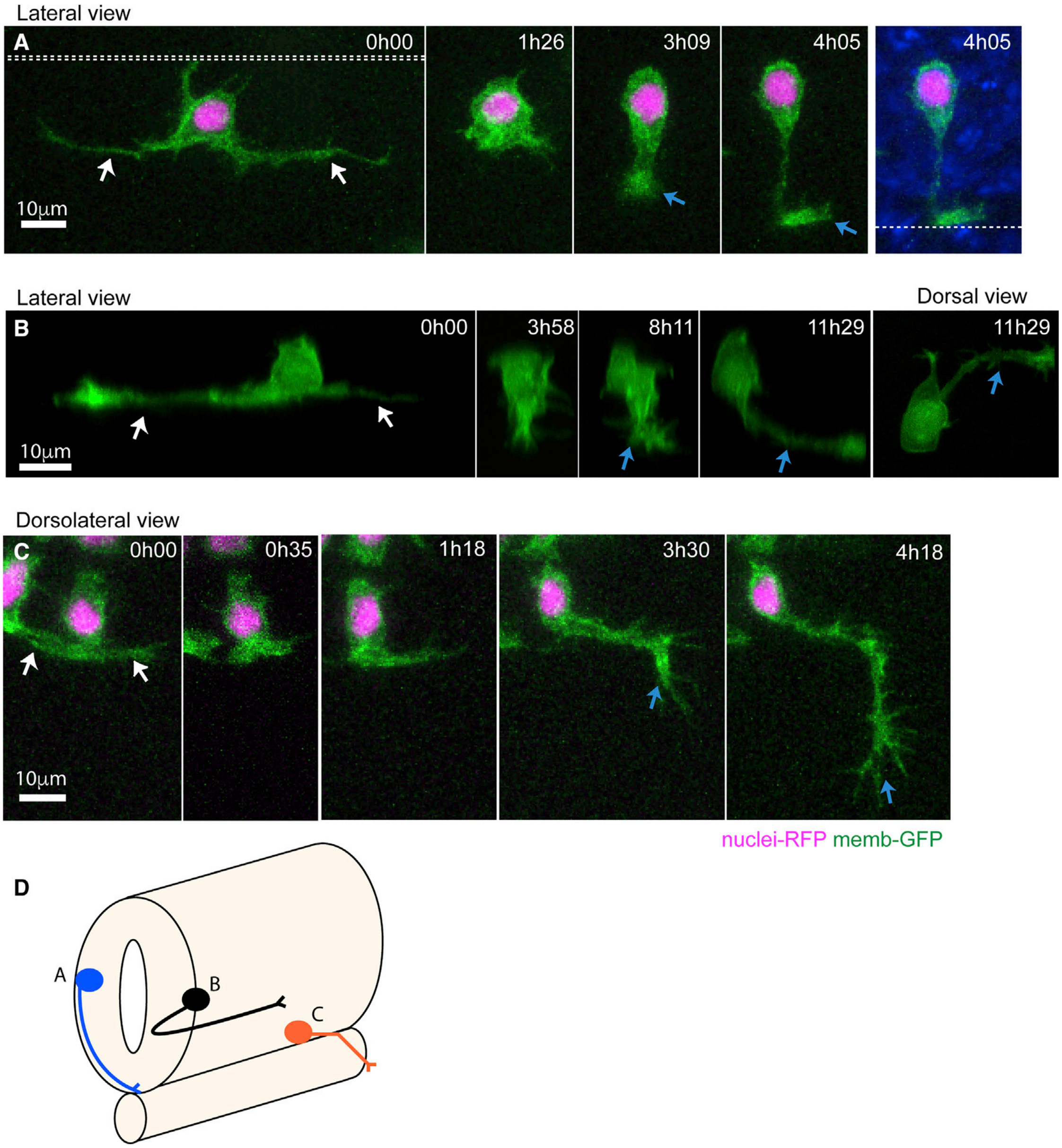

Figure 2. Stereotyped Axon Formation Follows Basal Protrusion Retraction

(A) Image sequence from a time lapse showing a neuron with long basal protrusions (white arrows) that are fully retracted before axon initiation (blue arrow at time 3h09). The axon grows circumferentially and crosses the ventral floor plate (blue arrow at time 4h05) (Video S2). Double dashed line shows the apical surfaces. Single dashed line is the ventral surface of the spinal cord.

(B) Image sequence from a time lapse shows a neuron with long basal protrusions (white arrows) that are fully retracted before axon initiation (blue arrow at time 8h11). The axon is initiated from the ventral surface of the neuron and then grows longitudinally and ipsilaterally along the spinal cord (blue arrow at time 11h29).

(C) Image sequence from a time lapse of a motor neuron with short basal protrusions (white arrows) that are retracted by time point 0h35. The exact point of axon extension is not clear, but the axon (blue arrow) changes direction to leave ventral spinal cord and grow into muscle at time 3h30.

(D) Summary diagram of neuron morphologies shown in (A)–(C). Neurons were labeled with membrane-GFP (green) and H2B-RFP to show nuclei in A and C. All images are projected images from confocal z stacks.

radial process and usually immediately follows the detachment of the neuron from the apical surface (Figure 3C; Video S4).

These observations demonstrate that the programs of axogenesis and apical release are regionally distinct, suggesting a region-specific role for the T-shape transition in spinal differentiation.

Neurons Rarely Differentiate Close Together in Time and Space

To quantify the spatiotemporal dynamics of spinal neuron differentiation, we used *in vivo* confocal microscopy to determine the spatiotemporal pattern of differentiation of Vsx1:GFP-expressing neurons in the zebrafish spinal cord. Vsx1:GFP neurons are

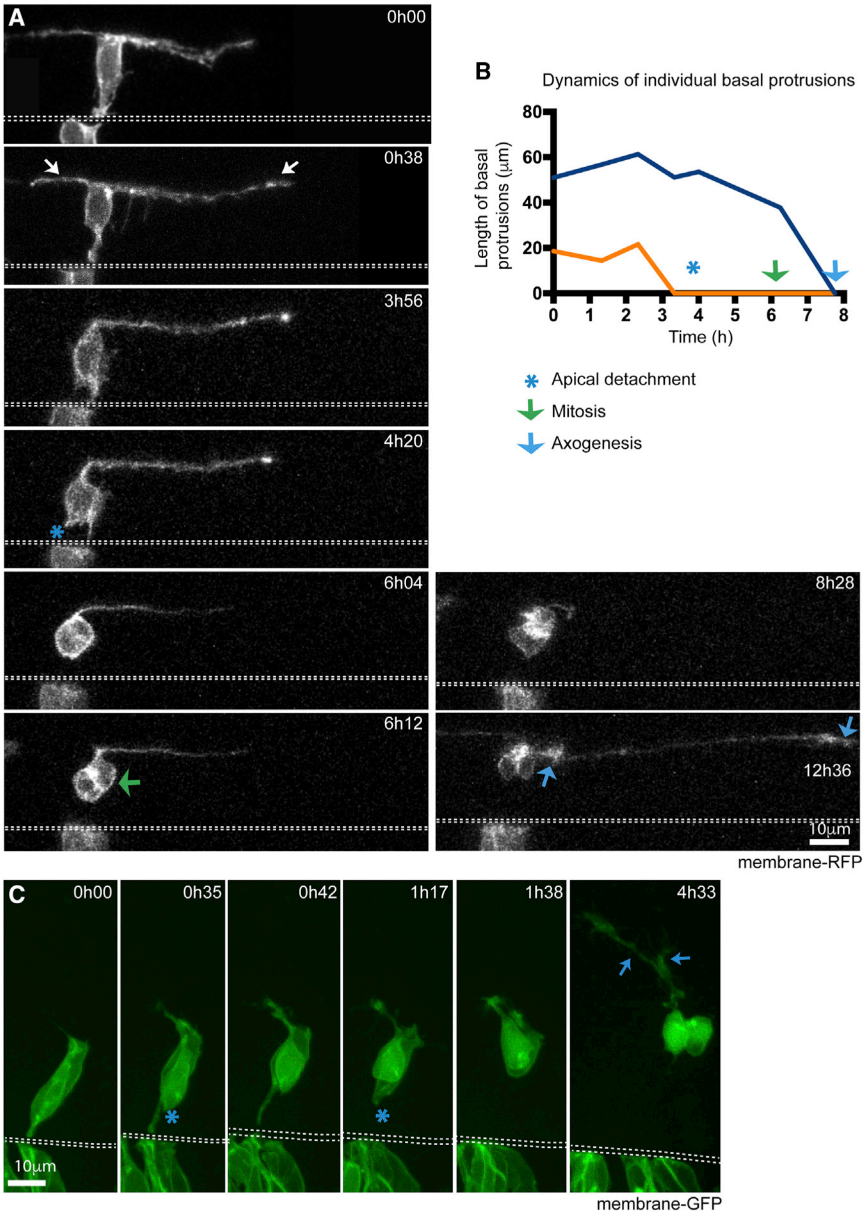

Figure 3. Spinal Non-apical Progenitors but Not Newborn Telencephalic Neurons Extend Basal Protrusions prior to Apical Detachment

(A) Image sequence showing a non-apical progenitor (NAP) with elongated basal protrusions (white arrows). The NAP retracts the apical attachment (blue asterisk in time point 4h20) before basal protrusions fully retract. Following apical detachment, the cell body rounds up away from apical surface of the neuroepithelium and undergoes mitosis (green arrow at time point 6h12). The NAP is neurogenically committed and produces two neuron daughters, each initiating axon growth at different time points (blue arrows indicate two growth cones at time 12h36) (Video S3). The apical surface is outlined by white dashed line. View is dorsal. All images are projected images from confocal z stacks.

(B) Graph showing the changes in length over time of the two basal protrusions from the NAP shown in A. Time points of when apical detachment, mitosis, and first axon elongation take place are indicated.

(C) Image sequence from time lapse showing a pair of differentiating telencephalic neurons. Long basal protrusions are not observed. Short basal protrusions from time point 0h35 on are the initial growth of axons. The neurons detach from the apical surface at 0h35 and 1h17 (blue asterisks). Extending axons are visible at 4h33 (blue arrows) (Video S4). Dashed lines show apical surfaces. Images are projections of confocal z stacks. View is dorsal.

born in pairs from the terminal division of *vsx1*-expressing NAPs (Kimura et al., 2008; McIntosh et al., 2017). GFP is detected in their progenitor immediately before terminal division and maintained in their daughters (Figure 4A). The appearance of adjacent GFP-expressing daughters thus offers a distinct and easily recognized time point to record as the start of differentiation of those neurons (Figure 4A). Using this criterion, we recorded the position and time of the start of differentiation of every pair of Vsx1 positive neurons in a 250- to 400-μm length of spinal cord at the level of somites 9–14 and between 19 and 27 hpf. We did this for both left and right sides in 17 embryos, thus recording 449 Vsx1 differentiation events in space and time within 34 equivalent stretches of spinal cord (Figure 4B; Data S1).

These data confirm that Vsx1 neurons differentiate in a long-distance spacing pattern with later born neurons differentiating in the gaps between already existing neurons (Figure 4C; Video S5). Time-lapse movies show no evidence that Vsx1 neurons or their progenitors migrate into this space; rather, these cells maintain stable positions. This pattern of sequential differentiation in the gaps continues for the next 6 h, at which time a near continuous line of Vsx1 neurons has been generated (Figures 4B and 4C; Video S5; Data S1).

To quantify this spatiotemporal pattern of differentiation, we looked at the timing of Vsx1 differentiation events that happened less than 20 μm apart. Neuroepithelial cells are typically 10.5 ± 4.1 μm (mean ± SD, n = 95 cells) wide at their basal pole, so this correlates to less than two cell diameters. Of the 449 Vsx1:GFP differentiation events, in only 7 cases (1.6%) were the differentiation events closer in time and space than 20 μm and 60 min apart (Figure 4D). The majority (68.3%) of events that occurred within 20 μm occurred between 2 and 3.5 h apart. Additionally, most consecutive Vsx1 differentiation events (i.e., those that occur closest in time) occur at a distance of 50–60 μm (Figures 4E and 4F).

These data suggest the presence of a mechanism that regulates the spatiotemporal differentiation of Vsx1 neurons in order to sequentially transform a long-distance spacing pattern into a continuous column of neurons.

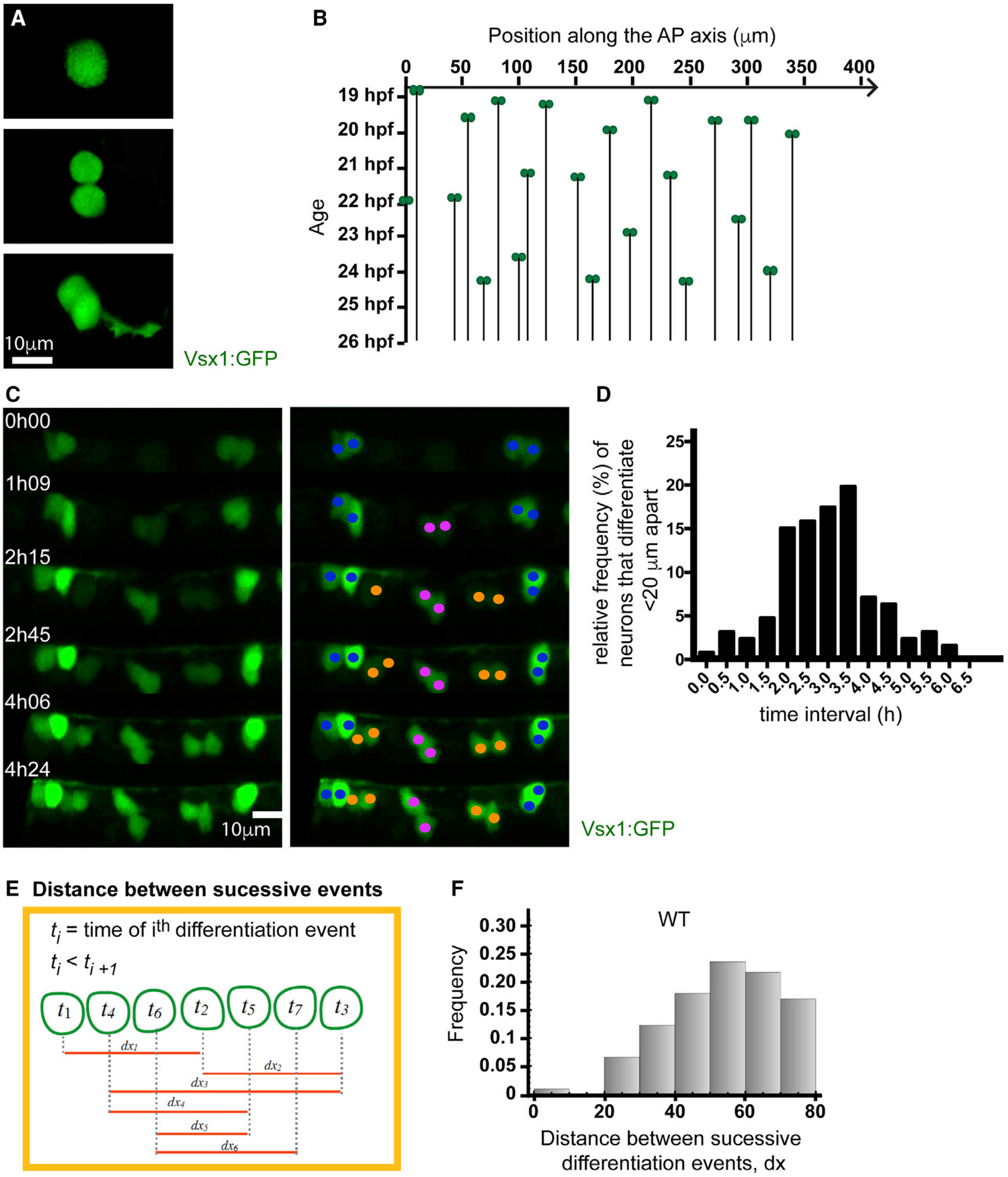

Figure 4. Neurons Rarely Differentiate Close Together in Time and Space

(A) Vsx1:GFP expression in a single cell before, during, and after a NAP division. Following mitosis, GFP expression is maintained, and axogenesis can be followed in both daughter neurons.

(B) Spatiotemporal pattern of Vsx1:GFP neuronal precursor differentiation from 19 to 27 hpf. The location of Vsx1:GFP NAPs at the time of mitosis are represented as pairs of green circles and plotted in space (x axis) and time (y axis). The black lines descending through time from the pair of green circles represent the position held by the daughter cells after mitosis.

(C) Image sequence from a time lapse showing the differentiation of Vsx1:GFP neurons in one section of spinal cord through time. The left panel shows Vsx1:GFP neurons differentiating over time. In the right panel, cells have been color coded to denote sister pairs. All images are projections from small confocal z stacks. See also Video S5.

(D) Frequency distribution showing the difference in time between Vsx1:GFP mitoses that occur less than 20 μm apart.

(E) Diagram illustrating the method used to calculate the distance between successive Vsx1:GFP differentiation events from a time-lapse movie. *t* indicates the time of differentiation and *dx* the distance between successive differentiation events.

(F) Histogram showing the distribution of the distance between successive Vsx1:GFP differentiation events in wild-type embryos.

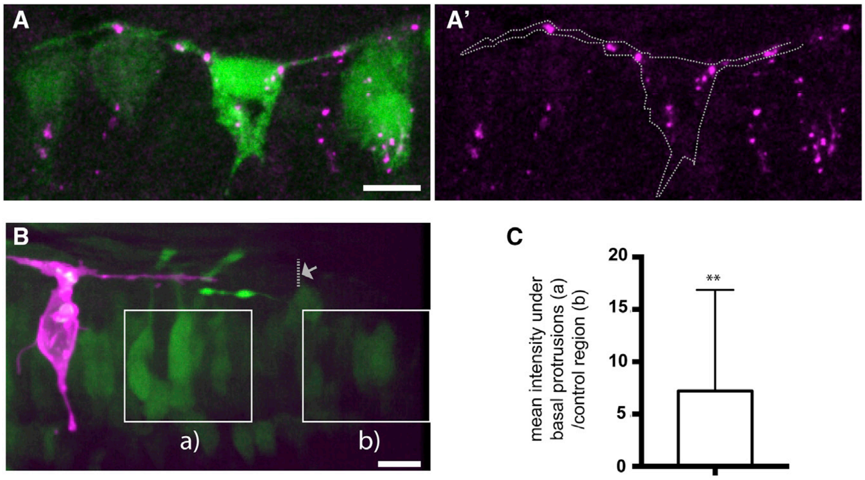

Figure 5. Transient Basal Protrusion Express Delta Protein, and Notch Activity Is Upregulated in Their Vicinity

(A and A′) DeltaD immunoreactivity (magenta) shows the localization of DeltaD aggregates in the basal protrusions and cell body of a T-shaped cell. The T-shaped cell expresses cytoplasmic GFP (green) under the DeltaD promotor.

(B) A T-shaped cell labeled with membrane-mKate (magenta) extends basal protrusions in a Tg(Tp1:VenusPEST) (green) embryo. The maximal extension of one basal protrusion is labeled with an arrow and dotted line. Squares indicate the two areas used for analysis of Tp1:VenusPEST expression.

(C) Graph showing the relative mean Tp1:VenusPEST fluorescence intensity under the basal protrusions compared to a control region outside the basal protrusions (unpaired one-tailed t test, p-value = 0.016, n = 13 basal protrusions (8 cells), the average (a intensity/ b intensity) is significantly greater than 1, mean ± SD = 7.2 ± 9.7).

Transient Basal Protrusions Express DeltaD and Notch Activity Is Upregulated in Their Vicinity

Our previous section analyzed Vsx1 neurons to show that neuronal differentiation in the embryonic zebrafish spinal cord occurs with an initial sparse pattern followed by sequential infilling (Figure 4). Similar patterns of differentiation are also apparent in previous studies of other neuronal subtypes (Gribble et al., 2009; Hutchinson and Eisen, 2006, Hutchinson et al. 2007; Kimura et al., 2008; England et al., 2011). This data suggests a mechanism may exist to transiently inhibit neuronal differentiation over a distance of several cell diameters from each newly differentiating cell and that this mechanism is sequentially released to allow differentiation in the initially inhibited space. We hypothesize that the transient basal protrusions on newly differentiating neurons and NAPs could mediate lateral inhibition at a distance in time and space. Since Delta-Notch signaling has been suggested to mediate lateral inhibition at a distance to regulate sparse pattern formation in other systems (reviewed in Pröls et al., 2016), we tested whether the transient basal protrusions on differentiating neurons could potentially mediate transient Delta-Notch signaling in our system.

Using an antibody against the DeltaD protein and a DeltaD transgenic reporter line Tg(*DeltaD:GAL4c;UAS:GFP*) (Scheer et al., 2001), we were able to determine that the *DeltaD* transgene highlights cells with typical T-shape morphology and that DeltaD protein is specifically enriched in the basal protrusions and cell body of these cells (Figures 5A and 5A′). Furthermore, if the basal protrusions participate in long-range lateral inhibition we expect them to activate Notch signaling pathway in the surrounding cells contacted by the basal protrusions. Importantly, this should occur in cells out of range of contact from the neuronal cell body. To test whether this is the case, we randomly labeled differentiating neurons in the Notch reporter line Tg(TP1:VenusPEST) (Ninov et al., 2012) and monitored the dynamics of Notch activation in nearby cells. We measured the relative mean intensity values of VenusPEST expression in a neuroepithelial region contacted by the labeled basal protrusion (but not the neuronal cell body) and compared it to a control region that had not been contacted by an identified protrusion (Figure 5B). We assessed VenusPEST expression 2 h after basal protrusions reached their maximum length. We found the amount of VenusPEST expression is significantly increased in regions spatially related to the identified protrusions when compared to the control region (Figure 5C). These observations are therefore consistent with the hypothesis that basal protrusions activate Notch signaling in order to delay neuronal differentiation in cells at a distance from the differentiating neuronal body.

Since basal protrusions extend bidirectionally along the same D/V level as the differentiating cell body, these protrusions will be perfectly placed to preferentially interact with neural progenitors located at the same D/V level (i.e., progenitors likely to generate neurons of the same subtype) and promote the neuronal spacing pattern observed in the zebrafish spinal cord. This suggests that the relative positions of neurons of different subtypes could be independent of each other. To test this, we measured the relative positions between different neuronal subtypes (*evx1*, *eng1b,* and Vsx1:GFP; Figures S3A–S3H). This analysis revealed that positions of *evx1* and *eng1b* neurons had no consistent alignment with Vsx1:GFP-expressing neurons (Figures S3F–S3H), suggesting that there is no pre-pattern for the relative position of different neuronal subtypes along the anteroposterior axis, and that regulation of differentiation of a particular neuronal subtype is independent of interactions with neurons of other subtypes.

Together, these results are consistent with the existence of a long-distance lateral inhibition mechanism that operates between differentiating neurons of the same subtype and their progenitors at the same D/V level. The expression of DeltaD in transient basal protrusions and the increase in Notch activation in cells spatially related to these basal protrusions suggests the basal protrusions could control both the spatial and temporal pattern of differentiation through long distance but transient Notch-Delta lateral inhibition.

Laminin Depletion Reduces Both Basal Protrusion Length and Spacing between Successively Differentiating Neurons

To further test whether basal protrusions could regulate the spatiotemporal pattern of Vsx1 neuron differentiation, we

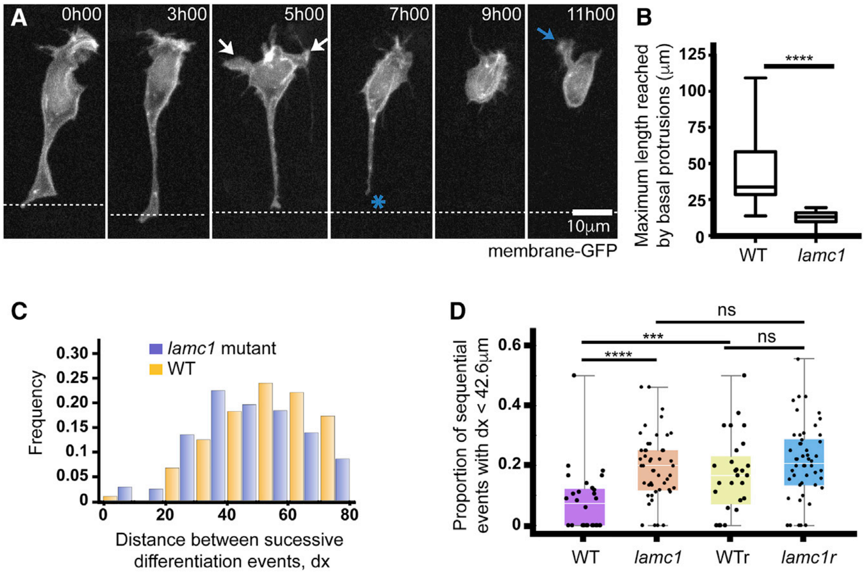

Figure 6. Laminin Depletion Reduces Basal Protrusion Length and Spacing between Successively Differentiating Neurons

(A) Time-lapse sequence showing a differentiating neuron in a Laminin-depleted spinal cord (see Video S6). It has only short basal protrusions (white arrows in time point 5h00). The short basal protrusions are retracted before detachment from apical surface (blue asterisk at 7h00) and axon initiation (blue arrow at 11h00). Cell is labeled with membrane-GFP. View is dorsal.

(B) Box-and-whisker plot showing the maximum length reached by basal protrusions in wild-type (mean ± SD, 42.6 ± 20.2 μm, n = 24 cells) and *lamc1*-mutant embryos (mean ± SD, 12.3 ± 4.7 μm, n = 39 cells). The line inside the box represents the median and whiskers represent minimum and maximum values. Data analyzed using unpaired one-tailed Mann-Whitney test (p-value < 0.0001).

(C) Histogram showing the distribution of the distance between successive Vsx1:GFP differentiation events in wild-type (orange) and *lamc1*-mutant embryos (purple) (mean ± SD, 54.00 ± 1.52 μm in wild-type and 45.3 ± 0.99 μm in *lamc1*, one-tailed t test p-value = 3.16 10^{-6}).

(D) Graph showing the proportion of successive Vsx1:GFP differentiation events that occur within a 42.6-μm interval (the average size of wild-type basal protrusions) in wild-type embryos, *lamc1* embryos, randomized wild-type distributions and randomized *lamc1* distributions. The Kolmogorov-Smirnov test was used to compare wild-type and *lamc1* distributions (p-value = 0.000066); wild-type and randomized wild-type distributions (p = 0.000224); and, *lamc1* and randomized *lamc1* distributions (p-value = 0.213).

modified basal protrusion length and quantified the pattern of neuronal differentiation *in vivo*. Since the transient basal protrusions grow at the basal surface of the neuroepithelium, we predicted that extracellular matrix proteins in the basement membrane could be required for their growth. To test this, we monitored neuronal differentiation in *lamc1* mutants that have no detectable Laminin at the basal surface of the neuroepithelium at the developmental stages we are studying. Neurons differentiating in *lamc1* mutant spinal cords develop significantly shorter basal protrusions (mean ± SD, 12.3 ± 4.7 μm, n = 39) than neurons in wild-type embryos (mean ± SD: 42.6 ± 20.2 μm, n = 24 cells, unpaired one-tailed t test p-value < 0.0001) (Figures 6A and 6B; Video S6), consistent with a role for Laminin in basal protrusion extension.

To determine whether the reduced length of basal protrusions in Laminin-depleted embryos could affect the spatiotemporal pattern of neuron differentiation, we performed time-lapse microscopy and compared the pattern of differentiation of Vsx1:GFP neuron pairs in *lamc1* mutants (Data S1) (n = 721 differentiation events in 50 stretches of spinal cord in 25 embryos) and wild type. We found that successive differentiation events occur closer together in *lamc1* mutants than in wild type (Figure 6C, mean ± SD, 54.00 ± 1.52 μm in wild type and 45.3 ± 0.99 μm in *lamc1*, one-tailed t test p-value = 3.16 × 10^{-6}), with the highest frequency of these events occurring 30–40 μm apart in the mutant compared to 50–60 μm apart in the wild type (Figures 4F and 6C).

Since wild-type basal protrusions extend 42.6 μm on average (and can potentially influence differentiation in this range), we then determined the proportion of sequential differentiation events that occurred within 42.6 μm of each other in the wild type and *lamc1* background. This verified that differentiation events are twice as likely to occur within this range in the *lamc1* mutant (0.19 ± 0.11) than in the wild-type embryos (0.080 ± 0.11) (Kolmogorov-Smirnov test, p-value = 0.0000666) (Figure 6D). We further compared the wild-type and *lamc1* differentiation data to randomly generated differentiation events and found that the proportion of sequential events that occurred within 42.6 μm in the wild type, but not the *lamc1* data, is significantly different from random (Kolmogorov-Smirnov test, p-value = 0.000224 and p-value = 0.213) (Figure 6D).

To discard the possibility that a decrease in neuronal spacing in *lamc1* mutants is due to an overall increase in neuronal differentiation we quantified the rate of neurogenesis. We determined the ratio of neurons to progenitors (N/P) at early stages of embryonic development and found no difference between wild type and mutant (Figures S4A and S4B). In addition, we analyzed the overall organization of the spinal cord in *lamc1* mutants and showed that patterns of polarity proteins, the locations of progenitor divisions and the location of neuronal differentiation are normal (Figures S4A–S4C). These experiments suggest that gross neuroepithelial organization and rates of differentiation are normal in *lamc1* mutant embryos at early stages of embryonic development.

Overall, these results are consistent with the hypothesis that basal protrusions transiently extend the range of influence of lateral inhibition and longer basal protrusions can regulate differentiation over a longer distance.

Theoretical Predictions Support the Role of Basal Protrusions in Patterning Differentiation through Delta-Notch-Mediated Lateral Inhibition

To determine whether the pattern of neuronal differentiation can be explained by Delta-Notch-mediated lateral inhibition delivered via transient basal protrusions, we developed a physical description of lateral inhibition coupled to the observed

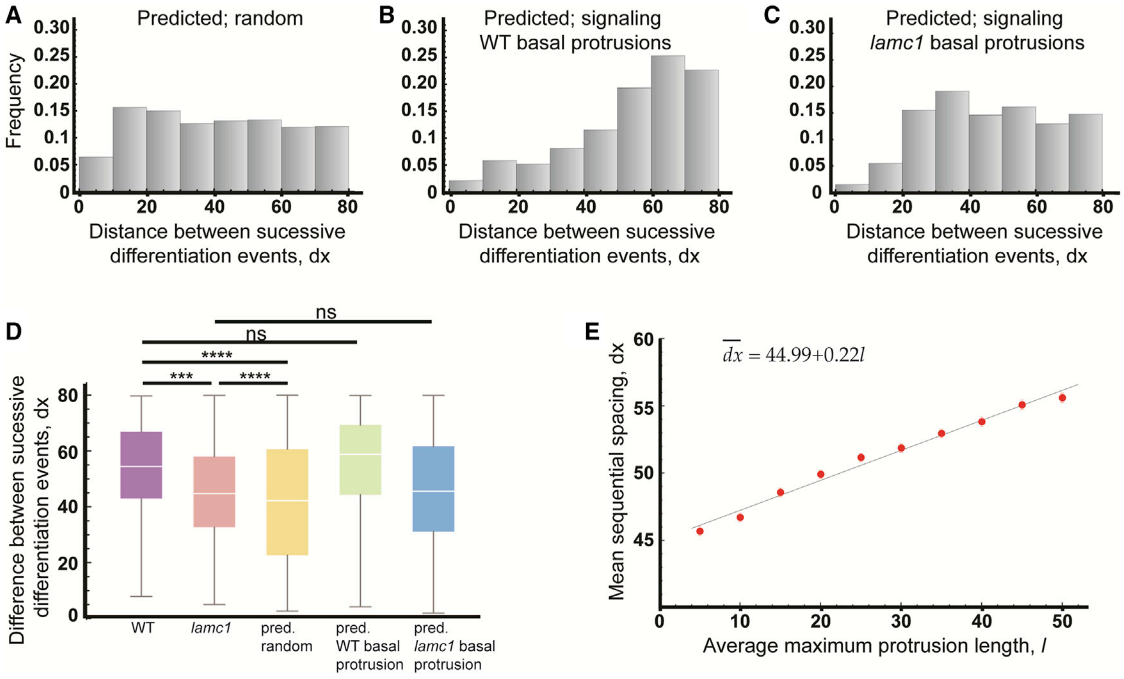

Figure 7. Theoretical Predictions Support the Role of Basal Protrusions in Patterning Differentiation Through Delta-Notch-Mediated Lateral Inhibition

(A–C) Histograms of the distributions of the distance between successive differentiation events predicted by theoretical model assuming a random distribution of differentiation events (A) (mean ± SD, 40.90 ± 21.55 μm), assuming lateral inhibition signaling occurs through basal protrusions of wild-type length (B) (mean ± SD, 54.53 ± 18.92 μm), or, assuming lateral inhibition signaling occurs through basal protrusions of *lamc1* length (C) (mean ± SD, 46.32 ± 18.68 μm).

(D) Box-and-whisker plots of the distance between successive differentiation events under various *in vivo* conditions and model predictions. The Kolmogorov-Smirnov test was used to compare wild-type and *lamc1* distribution (p-value = 0.000167), wild-type and predicted random distribution (p-value < E^{-12}), *lamc1* mutant and predicted random distribution (p-value = 9.6E^{-7}), wild-type and predicted distribution when basal protrusions of wild-type length convey lateral inhibition (p-value = 0.121), and *lamc1* mutant and predicted distributions when shorter and slower (*lamc1* length and dynamics) basal protrusions convey lateral inhibition (p-value = 0.181).

(E) Predicted relationship between the average maximum length of basal protrusions and the mean distance between sequential differentiation events.

protrusions dynamics. The dynamics of Delta-Notch signalling have been modelled extensively (Binshtok and Sprinzak, 2018). Here we built on Cohen et al. (2010) and Collier et al. (1996) and describe the process of lateral inhibition by,

$$\frac{dN}{dt} = R_N \frac{D_{in}^k}{a + D_{in}^k} - \mu N \quad \text{(Equation 1)}$$

$$\frac{dD}{dt} = R_D \frac{1}{1 + bN^h} - \rho N \quad \text{(Equation 2)}$$

$$D_{in} = \alpha \sum_{soma} D + \beta \sum_{protrusions} D. \quad \text{(Equation 3)}$$

These equations describe the dynamic process of gene activation and inhibition between signaling proteins in contacting cells. N and D refer to the amount of active Notch and Delta within cells, and D_{in} is the total signal received by a cell from all cells in contact with it. We assume that cells only mediate signaling through their protrusions and set $\alpha = 0$ and $\beta = 1$. Nonzero values of α are considered in the STAR Methods. We further assume that the probability of neuronal differentiation correlates with a cell's level of Delta expression (Hunter et al., 2016) and that neuronal differentiation commences with basal protrusion extension. The temporal and spatial dynamics of basal protrusions follow the experimentally observed dynamics. See STAR Methods for further details of the theoretical setup.

We first performed simulations to predict the distribution of *dx* assuming differentiation events occur randomly along the spinal cord (Figure 7A). If differentiation events occur at random, the distance between successive events should also be random. With random differentiation, the predicted distribution of *dx* (mean ± SD, 40.90 ± 21.55 μm) differs significantly from both the wild-type experimental distribution (mean ± SD, 54.53 ± 18.92 μm; Kolmogorov-Smirnov test, p-value < E^{-10}) (compare Figure 7A to Figure 4F), and the *lamc1* mutant distribution (mean ± SD, 46.32 ± 18.68 μm; Kolmogorov-Smirnov test, p-value = 9.6E^{-7}) (compare Figure 7A to Figure 6C), confirming that the spatiotemporal patterns of differentiation *in vivo* are unlikely to be randomly generated.

We then performed simulations assuming that the protrusion dynamics follow those of the wild-type fish. The predicted distribution between successive differentiation events (*dx*) in this case agrees with our experimental measurements (compare Figure 4F and Figure 7B; Kolmogorov-Smirnov test, p-value = 0.121). We repeated the analysis but now assuming that the length and dynamics of protrusions follow those of the Laminin-deficient *lamc1* mutant. Now the predicted distribution is in agreement

with the distribution of *dx* in the *lamc1* mutant found *in vivo* (compare Figure 6C to Figure 7C; Kolmogorov-Smirnov test, p value = 0.181). Furthermore, the *lamc1* mutant distributions are significantly different to simulations with wild-type length protrusions (compare Figure 6C to Figure 7B; Kolmogorov-Smirnov test p-value < E^{-10}). These results together suggest that the spatiotemporal dynamics of differentiation in wild-type and the *lamc1* mutant can both be explained by protrusion mediated lateral inhibition (Figure 7D). The differences in the distribution of *dx* between the wild-type and *lamc1* mutant can be explained by differences in basal protrusions length.

To understand how changes in basal protrusion length and dynamics impact on the spatiotemporal pattern of differentiation, we performed simulations while continuously varying the protrusion length. We found that the average distance between sequential events ($\overline{dx}$) follows a linear relationship with the protrusion length (Figure 7E). However, a given change in the protrusion length, *dl*, only confers a change in the mean spacing $\overline{dx}$, which is about 22% of *dl* (Figure 7E). This can be understood as follows. The protrusions specify a transient region where neurogenesis is inhibited. Although this generates a minimal spacing between sequential events, the events do not have to occur right at the boundary, and this alters the mean of the distribution (as seen in the noise around the peaks in Figures 4F, 6C, 7B, and 7C). This effect becomes stronger as the protrusions become smaller, which explains why large changes in the protrusion length in the *lamc1* mutant do not produce equally drastic shifts in the average value of *dx* (Figures 4F and 6C, see Quantification and Statistical Analysis in the STAR Methods and Figure S5). The relative impact of the protrusions on the spacing between sequential events in our region of interest declines for smaller and slower protrusions. These considerations together explain why large changes in the protrusion length in the *lamc1* mutant do not produce equally drastic shifts in the average value of dx (Figure 6C; see Figure S5 for detailed mathematical derivation and explanation).

To explore how the position and timing of differentiation are related, we also computed the spatial and temporal relationship between differentiation events (see STAR Methods). These analyses both *in vivo* and using our theoretical model showed that there is a negative correlation between the distance between two cells and the time at which they differentiate so that cells that are closer in space tend to differentiate further apart in time (Figures S6 and S7). *In vivo*, the wild-type and *lamc1*-mutant data both followed this trend; however, the range over which this correlation was present in *lamc1* mutants was reduced, consistent with the reduced basal arm length in *lamc1* mutants (Figures S6C and S6F). These spatiotemporal correlations also appear in our theoretical model when long or short basal protrusions mediate lateral inhibition (but not when differentiation occurs randomly), further supporting the role of basal protrusions in patterning neuronal differentiation (Figure S7).

Finally, we have performed simulations that assess differentiation patterns when lateral inhibition takes place only at soma-to-soma contacts and a combination of soma and basal protrusion contacts or only via basal protrusion contacts (STAR Methods). We found that including soma-to-soma lateral inhibition prior to protrusion extension cannot recapitulate our *in vivo* observations (Figure S8). This suggests that soma-to-soma contacts play a minimal role in the mechanism that determines the pattern of differentiation between spinal neurons.

DISCUSSION

Using live imaging in zebrafish, we have uncovered a cellular behavior for vertebrate neurons that regulates the spatiotemporal dynamics of neuronal differentiation along the spinal cord. Differentiating neurons and NAPs transiently develop two long basal protrusions prior to apical detachment and axogenesis. These basal protrusions express Delta at high levels and activate Notch signaling at a distance from the cell body. The dynamics of basal protrusion extension and retraction are consistent with a role in delivering Delta-Notch-mediated lateral inhibition at a distance to regulate the position and time of spinal neuron differentiation. Additionally, previous work has shown that Delta expression is required for the sparse spatial pattern of zebrafish spinal neurons (Okigawa et al., 2014). We show that experimental manipulation of basal protrusions *in vivo* and in a mathematical model of cells with and without signaling basal protrusions also support the role of basal protrusions in mediating lateral inhibition at a distance to regulate both the position and the time of spinal neuron differentiation. Protrusion-mediated lateral inhibition has been proposed to control sparse differentiation patterns in the fly peripheral nervous system (De Joussineau et al., 2003; Cohen et al., 2010). Our work demonstrates that a similar cell-protrusion-mediated mechanism operates in the spinal cord of a vertebrate.

The extension and retraction of basal protrusions on spinal neurons is highly stereotyped and is the earliest morphological feature of neuronal differentiation once the nucleus of the newly born neuron has reached the basal surface of the neural tube. Therefore, influencing the differentiative behavior of surrounding cells is prioritized over other essential neuronal behaviors such as axon outgrowth. Basal protrusions are robust microtubule based processes and always appear in pairs—one directed strictly anteriorly along the spinal cord and one directed strictly posteriorly. In contrast to the random protrusive activity observed on vertebrate neurons differentiating *in vitro* (Dotti et al., 1988), protrusive activity on spinal neurons differentiating *in vivo* is highly directed and predictable. We hypothesize that this directed longitudinal growth of basal protrusions is an effective way to preferentially contact and influence the behavior of neural progenitors at the same D/V level in the spinal cord. Progenitors from the same D/V level will likely generate neurons of the same subtype, and this directed basal growth maximizes the chance of influencing differentiation of similar neuronal subtypes. We find that NAPs (Vsx1-expressing progenitors) also undergo this predictable basal protrusive activity prior to their terminal division close to the basal surface of the spinal cord. They will therefore also be able to influence differentiation of similar NAPs. Thus, this morphological transition is another similarity between neurons and NAPs during their paths to differentiation (McIntosh et al., 2017).

Our analyses suggest neuronal basal protrusions deliver Delta-mediated lateral inhibition at a distance, a similar role to that proposed for the basal protrusions of SOPs on the fly notum and wing disk (De Joussineau et al., 2003; Cohen et al., 2010; Hunter et al., 2019). Although basal protrusions on SOPs and

spinal neurons share some similarities, there are some major differences between the two systems. SOPs radiate thin actin-based filopodia in all directions along the basal surface of the epithelium, while zebrafish neurons develop two substantial microtubule based protrusions that grow in predictable orientations. The basal protrusions of zebrafish neurons often also have filopodia on their surface, which may increase the interactions between differentiating cells and their near neighbors. Cell bodies of zebrafish neurons also have filopodia on their surface, although these are much shorter than the basal protrusions. Contrary to dynamic basal filopodia on SOPs, basal protrusions on spinal neurons remain relatively stable and extended for several hours. Importantly however, spinal neuron protrusions are transient, and their retraction releases cells from long-distance lateral inhibition and allows other neurons to differentiate in the previously inhibited space. This suggests that spinal basal protrusions regulate both the time and space of neuronal differentiation.

Protrusive activity that could influence surrounding cell behaviors has previously been suggested in the rodent cortex. There, basal intermediate progenitors (BIPs) in the rat and mouse subventricular zone have a large number of multidirectional membrane extensions that have alternatively been suggested to sense local factors prior to mitosis (Noctor et al., 2004) or to mediate Delta-Notch signaling between BIPs and apical radial glia cells, which maintains the proliferative progenitor population (Nelson et al., 2013). Although the protrusions on rodent progenitors do not appear to have a stereotypic orientation and their relation to the spatial and temporal progression of neurogenesis in the cortex has not been assessed, it remains possible that they serve similar functions to the basal protrusions of spinal neurons and progenitors. Our observations in the zebrafish telencephalon show that newborn neurons in this region behave quite differently to spinal neurons. Early telencephalic neurons do not elaborate long basal protrusions prior to axogenesis, and there is no obvious spatiotemporal pattern of differentiation in this region. Thus, programmes of cell morphogenesis and neuronal differentiation are region specific.

Many of the neurons in the spinal cord arise from asymmetrically fated divisions (Alexandre et al., 2010; Das and Storey, 2012; Saade et al., 2013; Kressmann et al., 2015) where daughter cell fate is also regulated by Delta-Notch interactions. In asymmetric divisions, Delta-Notch signaling is likely to be mediated exclusively between the sister cells of each division (Dong et al., 2012; Kressmann et al., 2015). Our modeling suggests that lateral inhibition between immediate neighbors cannot explain the long-distance spacing pattern of neuronal differentiation; nonetheless, this local mechanism that operates during progenitor divisions must be integrated with the long-distance mechanism delivered through basal protrusions. We have not investigated how these two processes might work together, but we favor the possibility that lateral inhibition through long basal protrusions delays neuron (and NAP) differentiation after their birth rather than regulating the time of their birth or particular fate. Our own unpublished data show that neurons born at the same time begin to express the neuronal transgene HuC:GFP within a very wide time window (4–12 h after their birth); thus, neurons can progress through their differentiation pathways at very different rates. Prospective neurons can initially maintain high levels of Notch activity, and reduction in Notch activation accelerates their differentiation (Baek et al., 2018), raising the possibility that the transient lateral inhibition mediated by basal protrusions controls the time of differentiation but does not change cell fate.

To test the potential for basal protrusions to mediate the spatial pattern of differentiation *in vivo*, we examined spinal neuron differentiation in Laminin-depleted spinal cords. We found that basal protrusion growth is significantly reduced in the absence of Laminin, and this correlates with a predicted reduction in the distance between differentiation events. Laminin depletion did not completely abolish basal protrusions from spinal neurons, and we show that the short protrusions that remain can explain the altered spatiotemporal dynamics of differentiation in the mutant. Although we cannot eliminate the possibility that Laminin depletion alters the spatial pattern of differentiation through mechanisms other than reduced basal protrusion length, this experimental approach is consistent with our major hypothesis. The overall architecture and cellular organization of the Laminin-depleted spinal cord is grossly normal, and we propose that a Laminin-rich extracellular matrix may be required for basal protrusion growth, perhaps in a similar way to Laminin's proposed role in axonal growth at the basal surface of neuroepithelium (Randlett et al., 2011).

Theoretical modeling that captures the protrusion dynamics in our *in vivo* system supports the hypothesis that basal protrusions mediate the spatiotemporal pattern of differentiation. We show that the spacing between successively born neurons is linear with protrusion length. Furthermore, our theoretical model recapitulates the spatiotemporal patterns *in vivo* in both wild-type and Laminin-depleted measurements. Interestingly, the inclusion of lateral inhibition via soma-to-soma signaling in our model introduces discrepancies between model output and *in vivo* data (Figure S8), suggesting that soma-to-soma signaling may be particularly weak during these events *in vivo*.

The biological function of regulating neuronal differentiation in a spatiotemporal manner is unclear. However, we speculate that it may be advantageous for neuronal circuit formation if the initial connections are made between a minimal number of spatially distributed neurons. Later, differentiating neurons can then be added to a functioning circuit to consolidate or modify the circuit function. This could be particularly important in zebrafish and amphibian embryos, as they develop externally and need to quickly build a functional motor circuit for survival.

STAR★METHODS

Detailed methods are provided in the online version of this paper and include the following:

- KEY RESOURCES TABLE
- CONTACT FOR REAGENT AND RESOURCE SHARING
- EXPERIMENTAL MODEL AND SUBJECT DETAILS
- METHOD DETAILS
    - *In Vivo* Experimental Details
    - Theoretical and Computational Details
- QUANTIFICATION AND STATISTICAL ANALYSIS
    - Analysis of *In Vivo* Data
    - Computational Analysis of Simulated and *In Vivo* Data

SUPPLEMENTAL INFORMATION

Supplemental Information can be found online at https://doi.org/10.1016/j.devcel.2019.05.035.

ACKNOWLEDGMENTS

We thank Buzz Baum for discussions and comments on the manuscript, Christian Sousa-Reid and Julian Lewis for the DeltaD antibody, Kate Lewis for the plasmids to produce the antisense probes for the *in situ* hybridization, Andy Symonds for the pCS2-mKate2-CAAX construct, Bill Harris for giving us the tg(Vsx1:GFP) line, Robert Knight and Sami Sultan for providing the tg(TP1:VenusPEST) embryos, Wellcome Trust Sanger Zebrafish Mutation Project for the *lamc1* mutant, and Leopold Laurer and Laura Ward for Figures S4B and S4C. Z.H. thanks Nicolas Levernier for discussions. We also thank the fish facilities at UCL and KCL and the imaging facility at UCL GOS UCL. This research was funded by an EPSRC fellowship (EP/L50488/) and HFSP Long Term Fellowship to Z.H., an MRC studentship to R. McIntosh, a Wellcome Clinical Research Career Development Fellowship (211112/Z/18/Z) to G.L.G., a Wellcome Investigator award to J.C. (102895/Z/13/Z), Royal Society fellowship to P.A. (DH100213), and NIHR Great Ormond Street Hospital Biomedical Research Centre. The views expressed are those of the authors and not necessarily those of the NHS, the NIHR, or the Department of Health.

AUTHOR CONTRIBUTIONS

P.A. and J.C. conceived the project, supervised the work, and wrote the manuscript. P.A. characterized the individual neuronal behaviors and prepared figures and movies. P.A. and R.E.M. characterized the neuronal behaviors and Vsx1 distribution in *lamc1* mutants. R.M. characterized the Vsx1 distribution in wild-type embryos. P.A. and R.E.M. obtained the Delta and Tg(TP1:VenusPEST) experimental data. G.G., P.A., and Z.H. developed the method to quantify Tg(TP1:VenusPEST). Z.H. developed the mathematical model, wrote all computational code, performed simulations, did most of the statistical analysis, and contributed to writing the manuscript. P.A. and Z.H. processed and analyzed all the Vsx1 experimental data. All authors commented on the manuscript.

DECLARATION OF INTERESTS

The authors declare no competing interests.

Received: August 21, 2018
Revised: February 26, 2019
Accepted: May 20, 2019
Published: June 17, 2019

REFERENCES

Alexandre, P., Reugels, A.M., Barker, D., Blanc, E., and Clarke, J.D.W. (2010). Neurons derive from the more apical daughter in asymmetric divisions in the zebrafish neural tube. Nat. Neurosci. *13*, 673–679.

Andersen, E.F., and Halloran, M.C. (2012). Centrosome movements in vivo correlate with specific neurite formation downstream of LIM homeodomain transcription factor activity. Development *139*, 3590–3599.

Appel, B., Givan, L.A., and Eisen, J.S. (2001). Delta-Notch signaling and lateral inhibition in zebrafish spinal cord development. BMC Dev. Biol. *1*, 13.

Baek, C., Freem, L., Goïame, R., Sang, H., Morin, X., and Tozer, S. (2018). Mib1 prevents Notch Cis-inhibition to defer differentiation and preserve neuroepithelial integrity during neural delamination. PLoS Biol. *16*.

Barnes, A.P., and Polleux, F. (2009). Establishment of axon-dendrite polarity in developing neurons. Annu. Rev. Neurosci. *32*, 347–381.

Batista, M.F., Jacobstein, J., and Lewis, K.E. (2008). Zebrafish V2 cells develop into excitatory CiD and Notch signalling dependent inhibitory VeLD interneurons. Dev. Biol. *322*, 263–275.

Bernhardt, R.R., Chitnis, A.B., Lindamer, L., and Kuwada, J.Y. (1990). Identification of spinal neurons in the embryonic and larval zebrafish. J. Comp. Neurol. *302*, 603–616.

Binshtok, U., and Sprinzak, D. (2018). Modeling the notch response. Adv. Exp. Med. Biol. *1066*, 79–98.

Buszczak, M., Inaba, M., and Yamashita, Y.M. (2016). Signaling by cellular protrusions: keeping the conversation private. Trends Cell Biol. *26*, 526–534.

Chitnis, A., Henrique, D., Lewis, J., Ish-Horowicz, D., and Kintner, C. (1995). Primary neurogenesis in Xenopus embryos regulated by a homologue of the Drosophila neurogenic gene Delta. Nature *375*, 761–766.

Cohen, M., Georgiou, M., Stevenson, N.L., Miodownik, M., and Baum, B. (2010). Dynamic filopodia transmit intermittent Delta-Notch signaling to drive pattern refinement during lateral inhibition. Dev. Cell *19*, 78–89.

Collier, J.R., Monk, N.A., Maini, P.K., and Lewis, J.H. (1996). Pattern formation by lateral inhibition with feedback: a mathematical model of delta-notch intercellular signalling. J. Theor. Biol. *183*, 429–446.

Craig, A.M., and Banker, G. (1994). Neuronal polarity. Annu. Rev. Neurosci. *17*, 267–310.

Dale, N., Roberts, A., Ottersen, O.P., and Storm-Mathisen, J. (1987). The development of a population of spinal cord neurons and their axonal projections revealed by GABA immunocytochemistry in frog embryos. Proc. R. Soc. Lond. B Biol. Sci. *232*, 205–215.

Das, R.M., and Storey, K.G. (2012). Mitotic spindle orientation can direct cell fate and bias Notch activity in chick neural tube. EMBO Rep. *13*, 448–454.

Das, R.M., and Storey, K.G. (2014). Apical abscission alters cell polarity and dismantles the primary cilium during neurogenesis. Science *343*, 200–204.

De Joussineau, C., Soulé, J., Martin, M., Anguille, C., Montcourrier, P., and Alexandre, D. (2003). Delta-promoted filopodia mediate long-range lateral inhibition in Drosophila. Nature *426*, 555–559.

Dong, Z., Yang, N., Yeo, S.-Y., Chitnis, A., and Guo, S. (2012). Intralineage directional Notch signaling regulates self-renewal and differentiation of asymmetrically dividing radial glia. Neuron *74*, 65–78.

Dotti, C.G., Sullivan, C.A., and Banker, G.A. (1988). The establishment of polarity by hippocampal neurons in culture. J. Neurosci. *8*, 1454–1468.

England, S., Batista, M.F., Mich, J.K., Chen, J.K., and Lewis, K.E. (2011). Roles of Hedgehog pathway components and retinoic acid signalling in specifying zebrafish ventral spinal cord neurons. Development *138*, 5121–5134.

Eom, D.S., Bain, E.J., Patterson, L.B., Grout, M.E., and Parichy, D.M. (2015). Long-distance communication by specialized cellular projections during pigment pattern development and evolution. eLife *4*.

Gribble, S.L., Kim, H.S., Bonner, J., Wang, X., and Dorsky, R.I. (2009). Tcf3 inhibits spinal cord neurogenesis by regulating sox4a expression. Development *136*, 781–789.

Hadjivasiliou, Z., Hunter, G.L., and Baum, B. (2016). A new mechanism for spatial pattern formation via lateral and protrusion-mediated lateral signalling. J. R. Soc. Interface *13*.

Henrique, D., Hirsinger, E., Adam, J., Le Roux, I., Pourquié, O., Ish-Horowicz, D., and Lewis, J. (1997). Maintenance of neuroepithelial progenitor cells by Delta-Notch signalling in the embryonic chick retina. Curr. Biol. *7*, 661–670.

Higashijima, S.-I., Mandel, G., and Fetcho, J.R. (2004b). Distribution of prospective glutamatergic, glycinergic, and GABAergic neurons in embryonic and larval zebrafish. J. Comp. Neurol. *480*, 1–18.

Higashijima, S.-i, Masino, M.A., Mandel, G., and Fetcho, J.R. (2004a). Engrailed-1 expression marks a primitive class of inhibitory spinal interneuron. J. Neurosci. *24*, 5827–5839.

Hunter, G.L., Hadjivasiliou, Z., Bonin, H., He, L., Perrimon, N., Charras, G., and Baum, B. (2016). Coordinated control of Notch/Delta signalling and cell cycle progression drives lateral inhibition-mediated tissue patterning. Development *143*, 2305–2310.

Hunter, G.L., He, L., Perrimon, N., Charras, G., Giniger, E., and Baum, B. (2019). A role for actomyosin contractility in Notch signaling. BMC Biol. *17*, 12.

Hutchinson, S.A., Cheesman, S.E., Hale, L.A., Boone, J.Q., and Eisen, J.S. (2007). Nkx6 proteins specify one zebrafish primary motoneuron subtype by regulating late islet1 expression. Development *134*, 1671–1677.

Hutchinson, S.A., and Eisen, J.S. (2006). Islet1 and Islet2 have equivalent abilities to promote motoneuron formation and to specify motoneuron subtype identity. Development *133*, 2137–2147.

Kettleborough, R.N.W., Busch-Nentwich, E.M., Harvey, S.A., Dooley, C.M., de Bruijn, E., van Eeden, F., Sealy, I., White, R.J., Herd, C., Nijman, I.J., et al. (2013). A systematic genome-wide analysis of zebrafish protein-coding gene function. Nature *496*, 494–497.

Kimura, Y., Okamura, Y., and Higashijima, S. (2006). alx, a zebrafish homolog of Chx10, marks ipsilateral descending excitatory interneurons that participate in the regulation of spinal locomotor circuits. J. Neurosci. *26*, 5684–5697.

Kimura, Y., Satou, C., and Higashijima, S.-I. (2008). V2a and V2b neurons are generated by the final divisions of pair-producing progenitors in the zebrafish spinal cord. Development *135*, 3001–3005.

Kressmann, S., Campos, C., Castanon, I., Fürthauer, M., and González-Gaitán, M. (2015). Directional Notch trafficking in Sara endosomes during asymmetric cell division in the spinal cord. Nat. Cell Biol. *17*, 333–339.

Kwan, K.M., Fujimoto, E., Grabher, C., Mangum, B.D., Hardy, M.E., Campbell, D.S., Parant, J.M., Yost, H.J., Kanki, J.P., and Chien, C.B. (2007). The Tol2kit: a multisite gateway-based construction kit for Tol2 transposon transgenesis constructs. Dev. Dyn. *236*, 3088–3099.

Lai, E.C. (2004). Notch signaling: control of cell communication and cell fate. Development *131*, 965–973.

McIntosh, R., Norris, J., Clarke, J.D., and Alexandre, P. (2017). Spatial distribution and characterization of non-apical progenitors in the zebrafish embryo central nervous system. Open Biol. *7*.

Megason, S.G., and Fraser, S.E. (2003). Digitizing life at the level of the cell: high-performance laser-scanning microscopy and image analysis for in toto imaging of development. Mech. Dev. *120*, 1407–1420.

Nelson, B.R., Hodge, R.D., Bedogni, F., and Hevner, R.F. (2013). Dynamic interactions between intermediate neurogenic progenitors and radial glia in embryonic mouse neocortex: potential role in Dll1-Notch signaling. J. Neurosci. *33*, 9122–9139.

Ninov, N., Borius, M., and Stainier, D.Y. (2012). Different levels of Notch signaling regulate quiescence, renewal and differentiation in pancreatic endocrine progenitors. Development *139*, 1557–1567.

Noctor, S.C., Martínez-Cerdeño, V., Ivic, L., and Kriegstein, A.R. (2004). Cortical neurons arise in symmetric and asymmetric division zones and migrate through specific phases. Nat. Neurosci. *7*, 136–144.

Norden, C., Young, S., Link, B.A., and Harris, W.A. (2009). Actomyosin is the main driver of interkinetic nuclear migration in the retina. Cell *138*, 1195–1208.

Okigawa, S., Mizoguchi, T., Okano, M., Tanaka, H., Isoda, M., Jiang, Y.J., Suster, M., Higashijima, S.-I., Kawakami, K., and Itoh, M. (2014). Different combinations of Notch ligands and receptors regulate V2 interneuron progenitor proliferation and V2a/V2b cell fate determination. Dev. Biol. *391*, 196–206.

Passini, M.A., Levine, E.M., Canger, A.K., Raymond, P.A., and Schechter, N. (1997). Vsx-1 and Vsx-2: differential expression of two paired-like homeobox genes during zebrafish and goldfish retinogenesis. J. Comp. Neurol. *388*, 495–505.

Pröls, F., Sagar, and Scaal, M. (2016). Signaling filopodia in vertebrate embryonic development. Cell. Mol. Life Sci. *73*, 961–974.

Randlett, O., Poggi, L., Zolessi, F.R., and Harris, W.A. (2011). The oriented emergence of axons from retinal ganglion cells is directed by laminin contact in vivo. Neuron *70*, 266–280.

Roberts, A., Dale, N., Ottersen, O.P., and Storm-Mathisen, J. (1987). The early development of neurons with GABA immunoreactivity in the CNS of Xenopus laevis embryos. J. Comp. Neurol. *261*, 435–449.

Saade, M., Gutiérrez-Vallejo, I., Le Dréau, G., Rabadán, M.A., Miguez, D.G., Buceta, J., and Martí, E. (2013). Sonic hedgehog signaling switches the mode of division in the developing nervous system. Cell Rep. *4*, 492–503.

Sanders, T.A., Llagostera, E., and Barna, M. (2013). Specialized filopodia direct long-range transport of SHH during vertebrate tissue patterning. Nature *497*, 628–632.

Scheer, N., Groth, A., Hans, S., and Campos-Ortega, J.A. (2001). An instructive function for Notch in promoting gliogenesis in the zebrafish retina. Development *128*, 1099–1107.

Schindelin, J., Arganda-Carreras, I., Frise, E., Kaynig, V., Longair, M., Pietzsch, T., Preibisch, S., Rueden, C., Saalfeld, S., Schmid, B., et al. (2012). Fiji: an open-source platform for biological-image analysis. Nat. Methods *9*, 676–682.

Sprinzak, D., Lakhanpal, A., LeBon, L., Garcia-Ojalvo, J., and Elowitz, M.B. (2011). Mutual inactivation of Notch receptors and ligands facilitates developmental patterning. PLoS Comput. Biol. *7*.

Sprinzak, D., Lakhanpal, A., Lebon, L., Santat, L.A., Fontes, M.E., Anderson, G.A., Garcia-Ojalvo, J., and Elowitz, M.B. (2010). Cis-interactions between Notch and Delta generate mutually exclusive signalling states. Nature *465*, 86–90.

Stanganello, E., Hagemann, A.I.H., Mattes, B., Sinner, C., Meyen, D., Weber, S., Schug, A., Raz, E., and Scholpp, S. (2015). Filopodia-based Wnt transport during vertebrate tissue patterning. Nat. Commun. *6*, 5846.

Thaëron, C., Avaron, F., Casane, D., Borday, V., Thisse, B., Thisse, C., Boulekbache, H., and Laurenti, P. (2000). Zebrafish evx1 is dynamically expressed during embryogenesis in subsets of interneurones, posterior gut and urogenital system. Mech. Dev. *99*, 167–172.

Thisse, C., and Thisse, B. (2000). High resolution whole-mount *in situ* hybridization. In The Zebrafish Book. A Guide for the Laboratory Use of Zebrafish (*Danio rerio*), Fourth edition, M. Westerfield, ed. (University of Oregon Press).

Westerfield, M. (2000). The Zebrafish Book. A Guide for the Laboratory Use of Zebrafish (Danio rerio), Fourth edition (Univ. of Oregon Press).

Wright, G.J., Giudicelli, F., Soza-Ried, C., Hanisch, A., Ariza-McNaughton, L., and Lewis, J. (2011). DeltaC and DeltaD interact as Notch ligands in the zebrafish segmentation clock. Development *138*, 2947–2956.

STAR★METHODS

KEY RESOURCES TABLE

REAGENT or RESOURCE	SOURCE	IDENTIFIER
Antibodies		
Mouse monoclonal anti-HuC/D (16A11)	Invitrogen	Cat#A-21271; lot1252835; RRID:AB_221448
Rabbit polyclonal anti-aPKC ζ (C-20)	Santa Cruz Biotechnology	Cat#SC-216; k0413
Mouse monoclonal anti-DeltaD (zdd2)	Cancer Research Technology	Cat#C7/2/14; lot255/06
Chicken polyclonal anti-GFP	Abcam	Cat#Ab13970; lotGR89472-7; RRID:AB_300798
Chemicals, Peptides, and Recombinant Proteins		
1-phenyl-3-(2-thiazolyl)-2-thiourea	Sigma-Aldrich	Cat#P4015
Sytox Green	ThermoFischer Scientific	Cat#S7020
Fast Red substrate	Roche	Cat#11758888001
MS-222	Sigma-Aldrich	Cat#E10521
Critical Commercial Assays		
SP6 mMessenger mMachine kit	Ambion	Cat#AM1340
Experimental Models: Organisms/Strains		
Zebrafish – Ekkwill	N/A	N/A (wildtype strain)
Zebrafish – AB/Tuebingen (wildtype strain)	N/A	N/A (wildtype strain)
Zebrafish – Tuepfel long fin	N/A	N/A (wildtype strain)
Zebrafish – Tg(*vsx1*:GFP)	Kimura et al., 2008	ZFIN ID: ZDB-FISH-150901-23998
Zebrafish – Tg(*deltaD:Gal4;UAS:GFP*)	Scheer et al., 2001	ZFIN ID: ZDB-FISH-150901-6106
Zebrafish – Tg(TP1:VenusPEST)	Ninov et al., 2012	ZFIN ID: ZDB-FISH-150901-8023
Zebrafish – *sleepy*; *lamc1*^{sa379} mutant	Kettleborough et al., 2013	ZFIN ID: ZDB-FISH-150901-23200
Zebrafish – Tg(*vsx1*:GFP);*lamc1*^{sa379}	This paper	N/A
Recombinant DNA		
Plasmid: pCS2-mCherry-CAAX (referred to as m-RFP)	Laboratory of Chi-Bin Chien; Kwan et al., 2007	N/A
Plasmid: pCS2-EGFP-CAAX (referred to as m-GFP)	Laboratory of Chi-Bin Chien; Kwan et al., 2007	N/A
Plasmid: pCS2-mKate2-CAAX (referred to as m-mKate2)	This paper	N/A
Plasmid: pCS2-H2B-RFP (referred to as n-RFP)	Laboratory of Steffen Schlopp; Megason and Fraser, 2003	N/A
Plasmid: pCS2-Eb3-GFP	Laboratory of William Harris; Norden et al., 2009	N/A
Software and Algorithms		
Volocity 3D Image Analysis Software	Perkin-Elmer	http://www.perkinelmer.com/pages/020/cellularimaging/products/volocity.xhtml; RRID:SCR_002668
Fiji	Schindelin et al., 2012	http://fiji.sc; RRID:SCR_002285
Wolfram Mathematica	Wolfram	https://www.wolfram.com/mathematica/; RRID:SCR_014448
Prism 7	GraphPad	https://www.graphpad.com/scientific-software/prism/; RRID:SCR_005375

CONTACT FOR REAGENT AND RESOURCE SHARING

Further requests for resources and reagents should be directed to and will be fulfilled by the Lead Contact, Paula Alexandre (p.alexandre@ucl.ac.uk). Details on theory and computational models can be obtained from Zena Hadjivasiliou (Zena.Hadjivasiliou@unige.ch).

EXPERIMENTAL MODEL AND SUBJECT DETAILS

All animal procedures were performed according to the UK Animal (Scientific Procedures) Act 1986 and carried out under Home Office Project Licence number PPL P70880F4C, which was subject to local AWERB Committee review and Home Office approval. The following zebrafish lines were used: Ekkwill, AB/Tuebingen, Tuepfel long fin, Tg(*vsx1*:GFP) (Kimura et al., 2008), Tg(*deltaD:Gal4*;*UAS:GFP*) (Scheer et al., 2001), Tg(TP1:VenusPEST) (Ninov et al., 2012), and *lamc1*^{sa379} mutant (*sleepy*; Kettleborough et al., 2013). Tg(*vsx1*:GFP) and *lamc1*^{sa379} lines were crossed to establish a Tg(*vsx1*:GFP);*lamc1*^{sa379} line. Adults were maintained under standard conditions as previously described (Westerfield, 2000), in a 14/10 hour light/dark cycle.

Embryos were obtained by natural spawning and raised in water or E2 medium at 28.5°C. If necessary, they were transferred to 0.003% 1-phenyl-3-(2-thiazolyl)-2-thiourea (Sigma-Aldrich) at 24 hpf to inhibit pigmentation.

Injections were performed at 16-64-cell stage. Embryos positive for mRNA expression, transgenic GFP expression and/or *lamc1*$^{sa379-/-}$ phenotype were selected for imaging. Live imaging was performed at 18-42 hpf. *In situ* hybridisation was performed at 22 hpf and immunohistochemistry at 22-28 hpf. Sex is not yet determined at these stages in zebrafish so was not taken into account.

METHOD DETAILS

In Vivo Experimental Details

Immunohistochemistry

Whole-mount immunohistochemistry was performed on wild type and *lamc1*$^{sa379-/-}$ embryos to assess neurogenesis and epithelial cell polarity, and on Tg(*deltaD:Gal4;UAS:GFP*) embryos to assess Delta protein expression. Embryos were fixed for 2 hours at room temperature in 4% PFA at 22-28 hpf. Primary antibodies used were against HuC/D (mouse anti-HuC/D, Invitrogen, diluted 1:200), aPKC (rabbit anti-aPKC, Santa Cruz Biotechnology, diluted 1:500), DeltaD (mouse anti-DeltaD, Cancer Research Technology, diluted 1:50) and GFP (chicken anti-GFP, Abcam, diluted 1:1000). Embryos were incubated with primary antibody for 2 to 3 days at 4°C in PBS Triton 0.5%, 2% BSA, 10% goat serum (detailed protocol described in Wright et al., 2011). Embryos were incubated in secondary antibodies overnight at 4°C in the same blocking solution. Sytox Green (ThermoFischer Scientific, diluted 1:2,000) was added with secondary antibody to label nuclei and show mitotic divisions.

In Situ *Hybridisation*

Embryos fixed for 2 hours at room temperature in 4% PFA at 22hpf were processed for whole-mount *in situ* hybridisation according to the protocol described in (Thisse and Thisse, 2000). For synthesis of antisense mRNA DIG-labelled probes DNA plasmids containing a cDNA fragment of *eng1b* (Batista et al., 2008), *evx1* (Thaëron et al., 2000), and *vsx1* (Passini et al., 1997) were linearised and the cDNA fragment was reverse transcribed using the RNA polymerases T3, T7, T3, respectively. Probes were detected in wild type embryos using Fast Red (Roche) substrate. Some embryos were incubated in Sytox Green (ThermoFischer Scientific, diluted 1:2,000) to label nuclei. To compare the relative distribution of neuronal subtypes, we performed *in situ* hybridisation for *eng1b* or *evx1* in Tg(*vsx1*:GFP) transgenic embryos followed by the detection of GFP expression by immunohistochemistry (chicken anti-GFP, Abcam, diluted 1:1000).

mRNA Injection

Plasmids containing cDNAs coding for the following fusion proteins were linearised and the mRNA synthesised using SP6 mMessenger mMachine kit (Ambion): membrane tagged RFP (mCherry-CAAX; referred to as m-RFP)(Kwan et al., 2007), m-GFP (EGFP-CAAX)(Kwan et al., 2007), m-mKate2, (mKate-CAAX)(this paper), nuclear tagged RFP (H2B-RFP; referred to as n-RFP)(Megason and Fraser, 2003), and Eb3-GFP(Norden et al., 2009). mRNA was injected into a single cell of wild type, *lamc1*^{sa379} mutant or Tg(TP1:VenusPEST) embryos at 16-64-cell stage to cause mosaic labelling and the embryos allowed to grow until imaging.

Confocal Imaging

Prior to imaging, live embryos were anaesthetised in MS-222 (Sigma-Aldrich). Fixed and live embryos were mounted in 1.5% low-melting point agarose (Sigma-Aldrich) in a petri dish with the dorsal spinal cord or dorsal telencephalon facing up. Fixed embryos were kept in PBS1x during imaging, while live embryos were kept at 28.5C in E2 medium containing MS-222 (Sigma-Aldrich) and 0.003% 1-phenyl-3-(2-thiazolyl)-2-thiourea (Sigma-Aldrich).

Live imaging of individual cells was performed to observe neuronal differentiation and Notch activation. mRNA-injected wild type, *lamc1*$^{sa379-/-}$ or Tg(TP1:VenusPEST) embryos were imaged on a spinning-disk confocal microscope using an UltraVIEW VoX system (Perkin-Elmer) built on a Nikon Ti-E microscope, with a 40x water-immersion objective with numerical aperture (NA) of 1.0. Z-stacks were acquired at 0.5-1 μm. A series of z-stacks were obtained every 3 to 8 minutes for between 3 and 20 hours from 16 hpf.

Live imaging was performed on Tg(*vsx1*:GFP) and Tg(*vsx1*:GFP);*lamc1*$^{sa379-/-}$ embryos to assess spatiotemporal dynamics of neuronal differentiation on a SP5 confocal (Leica) microscope with a 20x water-immersion objective with an NA of 0.95. Z-stacks were acquired at 1 μm every 5 to 8 minutes for 8-10 hours.

Fixed whole-mount tissue from *in situ* hybridisation and immunohistochemistry was imaged on a SP5 confocal (Leica) microscope (described above) or on a LSM880 laser scanning confocal (Zeiss) microscope equipped with a 20x water-immersion objective with an NA of 0.95.

Image Processing and Analysis

Individual basal protrusions were measured from the cell body to the periphery of the basal protrusion. The maximum overall length reached by basal protrusions includes the cell body width. This analysis was performed in 3D at single and multiple timepoints using

Volocity software (PerkinElmer). Images and movies shown in the manuscript result from a small projection of confocal z-stacks created using Fiji (Schindelin et al., 2012). Extra cells were occasionally removed from the field of view or pseudocoloured using Fiji to show examples of individual cells clearly.

To compare the intensity of Tg(TP1:VenusPEST) in the vicinity and away from the influence of the basal protrusions, we produced small z-projections, corrected drift and subtracted the background using Fiji. We used Fiji to measure the mean intensity values two hours after the basal protrusions reached their maximum length and analysed the area that had been in contact with the basal protrusions for at least 1h but was away from the neuronal cell body. For each case we calculated the ratio between the mean intensity under basal protrusions and control region (away from the basal protrusions).

Analysis of spatiotemporal dynamics of neuronal differentiation was performed at the level of somites 9 to 14 and between 19 and 27 hpf. The first appearance of adjacent GFP-expressing daughters following terminal division was considered to be the time of differentiation. Using Volocity, distances between temporally successive differentiation events were determined by measuring the distance (dx) between the last and the next neuronal pair born within a 80 μm (Figures 4F, 6C, and 7) and 42.6 μm (Figure 6D) space interval. The distance between neurons in fixed tissue was also measured using Volocity.

Theoretical and Computational Details

Lateral Inhibition Driven Differentiation

We used a mathematical model to simulate Notch-Delta mediated lateral inhibition. The model, as defined by Equations 1, 2, and 3 in the main text, describes the dynamics gene activation and inhibition via cell-cell signalling. D_{in} in Equation 3 is the total amount of incoming Delta summed over soma-to-soma and basal protrusion mediated contacts. The parameters α and β represent the relative amount of Delta at the soma-to-soma and in the basal protrusions respectively or the strength of the signal at the two locations. In the analysis presented in the main text we assumed that $\alpha = 0$ so that only basal protrusions mediate Notch signalling. We also relaxed this assumption (see Quantification and statistical analysis section "*Signalling at soma-to-soma*" and Figure S5) to investigate whether Notch signalling at soma-to-soma contacts could also be important. R_N and R_D are the baseline production rates for Notch and Delta molecules, a and k are parameters that determine how strongly incoming Delta induces Notch signalling, whereas b and h determine the strength of inhibition of Delta from Notch levels within the same cell. Finally, μ and ρ are the degradation rates of Notch and Delta, respectively.

We applied the model to a 1D array of cells of variable size following the measured size distribution. We developed a theoretical description of lateral inhibition and cell differentiation in a one dimensional tissue (i.e. a row of cells). We construct the row of cells by sampling cell diameters from a normal distribution with mean 11.10 μm and s.d. 4.51 μm, the experimentally measured values in the neuroepithelium. This captures the diversity seen in the cell width of differentiating neurons, dividing cells and neuroepithelial cells. We used our setup to simulate differentiation events in the row of cells under different conditions as described below and in the main text. Signalling dynamics in individual cells could then be fully defined by the coupled system of differential Equations 1, 2, and 3. Cells could make contact at the soma cell membranes and / or via basal cellular protrusions (Methods Image 1).

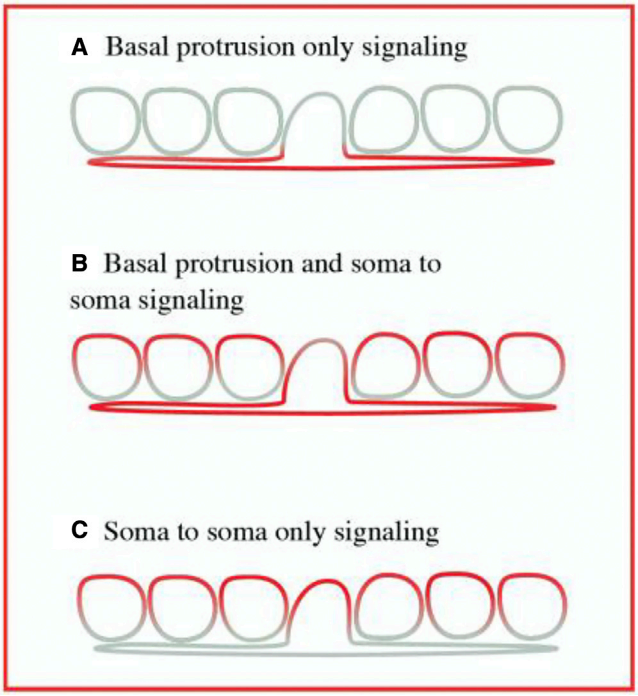

Methods Image 1: Signalling models considered in theoretical setup. Red indicates the presence and grey the absence of signalling. A: only basal protrusions can contribute to lateral signalling, B: basal protrusions and soma-to-soma contacts participate in lateral signalling, C: only soma-to-soma contacts contribute to lateral signalling.

Protrusion Dynamics

We modelled basal protrusion dynamics by allowing cells to extend protrusions if their Notch expression falls below a threshold (Hunter et al., 2016). Differentiating cells send but do not receive a signal (Sprinzak et al., 2010, 2011). Protrusions were extended at a constant rate and stopped growing when they reached length $> l_{max}$ where l_{max} was sampled from a normal distribution with mean 42.6 μm and s.d. 20.2 μm, following the *in vivo* measurements for maximum basal protrusion length. Once maximum length was reached the protrusions retracted at a rate 1.7 times faster than the extension rate (following *in vivo* dynamics). For the *lamc1* mutants we modified the distribution of l_{max} to follow the mutant distribution with mean 12.3 μm and s.d. 4.7 μm and implemented extension and retraction rates that were 1.4 times slower than the wild-type and retraction rates 2.5 times slower than the wild-type, following the rates measured experimentally. A cell was assumed to have differentiated when both its right and left protrusion were fully retracted. Differentiated cells no longer participated in signalling and we ran simulations until all virtual cells underwent differentiation.

We assume that cells begin extending their protrusions with a probability that depends on the levels of their Notch expression so that differentiation becomes more likely as Notch levels fall below a threshold. We implement this following previous work (Hunter et al., 2016) by computing the probability of entering differentiation using a Hill function,

$$P_{diff} = p\frac{N_{th}^q}{N_{th}^q + N^q} \quad \text{(Equation 4)}$$

for each cell, where N is the Notch expression of that cell and the parameters N_{th} and q determine a Notch threshold and the window around this threshold that lead to differentiation. The prefactor p is the upper limit of the likelihood of differentiation per time step in the simulation. Differentiated cells no longer participate in lateral inhibition. In addition, protrusions are high in Delta but are assumed to carry a negligible number of free notch receptors (e.g. due to cis-inhibition) and so they only send but do not receive a signal (Sprinzak et al., 2010, 2011).

The values of all model parameters for all figures presented in the main and supplemental text are provided on Methods Table 1.

Variable	Definition	
N	Notch levels	
D	Delta levels	
D_{in}	Total incoming Delta	
P_d	Arm extension and neuronal differentiation probability	
**Parameter (fixed)**	**Definition**	**Value**
R_N	Baseline Notch production rate	1.0
R_D	Baseline Delta production rate	1.0
a	Dissociation constant in Notch production	0.01
k	Hill coefficient in Notch production	2
b	Dissociation coefficient in inhibition of Delta by Notch	100
h	Hill coefficient in inhibition of Delta by Notch	3.0
μ	Notch degradation rate	1.0
ρ	Delta degradation rate	1.0
p	Maximal differentiation probability per unit time	0.2
N_{th}	Notch threshold for differentiation	10^{-4}
q	Hill coefficient for differentiation probability	1
**Parameter (varied)**	**Definition**	
α	Delta signaling amount/strength at soma-to-soma contacts	0 (Figure 8 , 0.1 (Figure S9))
β	Delta signaling amount/strength at basal protrusions	0 (Figure 8A), 1 (Figure 8 B-F; Figure S11D-F, Figure S14)
T_{ext}	Duration of basal protrusion extension period	0.05 (Figure 8B; Figure S11D-F; Figure S12A), 0.07 (Figure 8C; Figure S11G-I)

Methods Table 1: Definition of parameters in mathematical model. The table also indicates the values used for all figures that use simulated data in the main text and Supplemental Information.

Randomly Differentiating Tissue

We simulated a randomly differentiating tissue by initiating a row of cells as described above and then allowing cells to differentiate at random. In a row of n cells this corresponds to sampling from {1, 2, ..., n} without replacement and assuming that the ith sampled number is equivalent to the ith differentiation event. This allowed us to generate an ordered sequence of differentiation events and

then compute the distance between cells (corresponding to the index Numbers 1 to n) that were sampled successively (Methods Image 2). In this way we were able to predict the expected distance between successive events in a randomly differentiating tissue (Figure 7A).

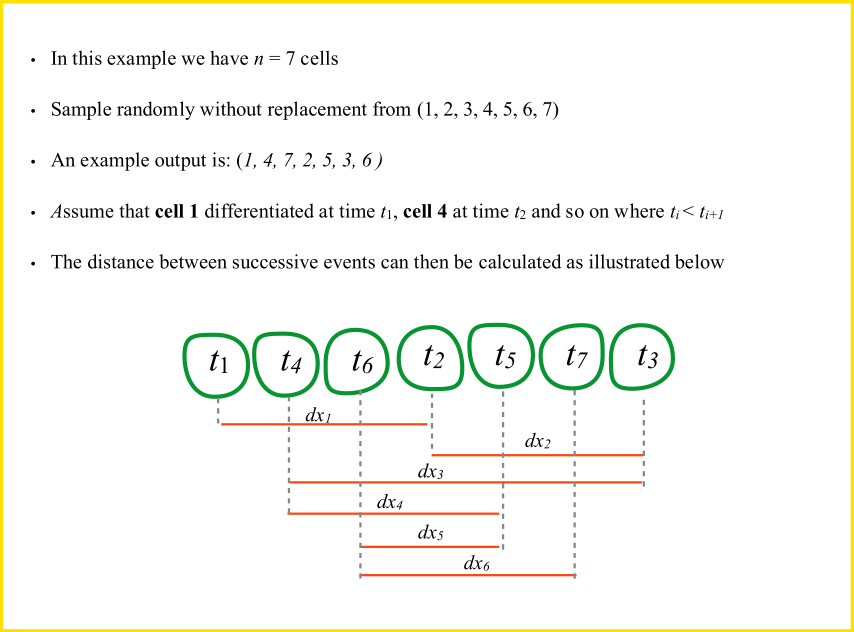

Methods Image 2: Algorithm for the generation of a randomly differentiating spinal cord. The positioning and size of cells were set according to experimental measurements.

Numerical Details

We initiate all simulations by randomly assigning each cell Notch and Delta levels sampled from $N(R_N, 0.01RN)$ and $N(R_D, 0.01RD)$ respectively where $N(\mu,\sigma)$ denotes the Normal distribution with mean μ and s.d. σ for values of R_N and R_D given on Methods Table 1. Following this the Notch and Delta levels of each cell evolve according to Equations 1, 2, and 3 which we solved numerically using the Euler method (Euler step set to 0.01). Furthermore, a Gaussian noise term was applied to initiate protein concentrations and to the concentrations at each time step in the simulation.

At each step in the simulation each individual cell has a probability of initiating protrusion extension that is computed using Equation 4. Cells that begin extending protrusions spend T_{ext} a.u. of time extending their protrusions and T_{ext} /1.7 a.u. of time retracting their protrusions, reflecting the relative amount of time cells were experimentally observed spending in the protrusion extension and retraction stages respectively. Once full protrusion retraction is achieved a cell is assumed to have differentiated to a neuron and no longer participates in the process of lateral signalling. The simulation parameter T_{ext} was set to 0.05 units of time for all wild-type simulations and 0.05*(mean length of experimental lamc1 mutant /experimental wilt-type basal protrusions)* dT units of time in short protrusion simulations where $dT = 1.4$ reflecting that lamc1 protrusions extended 1.4 times slower than wild-type basal protrusions. The retraction time in mutant protrusions in the simulations was set to 1.1 times their extension time, again reflecting experimental measurements. We further discuss the role of T_{ext} in the Quantification and Statistical Analysis section. A detailed outline of the algorithm we used throughout our analysis is shown on Methods Image 3. The numerical simulations produced a differentiation time for each individual cell together with its position. We used this information to compute (dx) as described in the main text (Figure 4C).

```
Establish a line of n cells with apical radial sizes A, based on measured values (mean = 11.10, sd = 4.51)
for cells i=1:n
        A(i) = Random Normal(mean,  s.d.) // diameter of ith cell

pos(1) = 0 // position of first cell
for cells i=2:n
        x(i) = x(i-1) + A(i-1) + A(i)  // all other cells positioned relative to previous cell and according to their size

Assign each cell levels of Notch and Delta
for cells i=1:n
        N(i) = Random Normal(1.0,  0.01)
        D(i) = Random Normal(1.0 ,  0.01)

At each time step update Notch, Delta and arm distribution
For each time step
        For all cells i=1:n
                // Compute incoming delta from cell membrane contacts from immediate neighbours
                D_in_CM (i) = D(i+1) + D(i-1)

                // Compute incoming delta from basal protrusion contacts
                For all other cells j = i:n; j != i
                        If (i>j && DIF(j)==0 && (jth cell's right arm's length) >0 && (jth cell's right arm's length) >=
                        pos(i)-pos(j)) //DIF(j) = 0 if jth cell has not differentiated and 1 otherwise
                        OR
                        If (i<j && DIF(j)==0 && (jth cell's left arm's length) > 0 && (jth cell's left arm's length) >=
                pos(j)-pos(i))
                        Then
                        D_in_BP(i) = D_in_arms(i) + D(j)

                D_in_total(i) = alpha* D_in_CM(i) + beta* D_in_BP(i)

                // Update Notch levels
                Notch(i) = Notch(i) + (– μ.Notch(i) + R_N.D_in(i)k /(a+D_in_total(i)k ))*dt +
                RandomNormal(0,Notch(i)*rand_error) // random_error = 0.01 and dt = 0.05
                // Update Delta levels
                Delta(i) =  Delta(i) + (– ρ.Notch(i) + R_D 1 /(1 + b*Notch(i)h ))*dt
                + RandomNormal(0,Delta(n)*rand_error)

                // Update basal protrusions if time > 1.0 a.u.
                        If cell i has no basal protrusions, initiate extension with probability equal to:
                                value = 1.0 – p*N(i)^q/( N(i)^q + N_th(i)^q)

                        If basal protrusions extending, extent further by eurler_step*T_ext:
                                BP_left_L(i) = BP_left_L(i) + eurler_step*T_ext*Normal(F_mean, F_SE )
                                BP_right_L(i) = BP_right_L(i) + eurler_step*T_ext*Normal(F_mean, F_SE)
                                //F_mean = 42.3, F_SE = 3.09

                        If a basal protrusion has exceeded Normal(F_mean, F_SE ) initiate retraction.

                        If basal protrusion retracting, retract further by eurler_step*T_ext*1.7
                                BP_left_L(i) = BP_left_L(i) - eurler_step*T_ext*1.7* Normal(F_mean, F_mean*0.1 )

                                BP_right_L(i) = BP_right_L(i) - eurler_step*T_ext*1.7* Normal(F_mean,
                                F_mean*0.1 )

                        When retraction complete, cell differentiates and exits signalling

Return differentiation time and position for each cell
```

Methods Image 3: Pseudo code. Outline of the algorithm used to generate simulated data.

Simulation code was written on c/c++. Simulated and experimental data were analysed using scripts written on Wolfram Mathematica (Inc.).

Comparison of Simulated Data to Experiments

We performed simulations on a row of 50 cells (mean cell diameter = 11.10 μm). The simulation was run until all cells differentiated. We repeated simulations for a given set of parameters 100 times to generate data for each experiment. We recorded the time and position of all events, computed the distance between sequential differentiation events and analysed all sequential events that occurred within 80 μm of one another. The spatial impact of the protrusions is only present at a length-scale that is relevant to the protrusion length. When our system is viewed at much larger length scales the spatiotemporal patterns we report become irrelevant. We therefore restricted our analysis to cells that differentiated within 80 μm of one another, approximately two times the average protrusion length. The simulated data was then used to obtain the distribution of the distance between successively differentiating cells *dx*. We performed this analysis for randomly differentiating tissues and for tissues where differentiation was influenced by different models of lateral inhibition (basal protrusions only, soma-to-soma only, basal protrusions and soma-to-soma; Methods Image 1). The simulated data shown in the main text assume only basal protrusions mediate signalling. The role of soma-to-soma signalling is explored in a latter section.

QUANTIFICATION AND STATISTICAL ANALYSIS

Analysis of *In Vivo* Data

To compare the maximum average length of cellular protrusions in neuronal and non-neuronal cells we used unpaired, one-tailed Mann-Whitney test. To compare the average maximum distance reached by basal protrusion in the wild type and *lamc1* mutant we applied the unpaired, one-tailed Mann-Whitney test. We used the Kolmogorov-Smirnov test to compare the differences in distribution of successive Vsx1 differentiation events between wild type, *lamc1* mutant and simulated data and unpaired one-tailed t-test to compare their means. We used the Kolmogorov-Smirnov test to compare the proportion of successive differentiation events occurring within 42.6 μm in wild type and *lamc1* mutant. The relative position of different neuronal subtypes was analysed using the Kruskal-Wallis with Dunn's multiple comparison test. Analysis of the intensity of Tg(TP1:VenusPEST) in the vicinity and away from the influence of the basal protrusions was performed using one-tailed paired t-test.

Embryos were included in the study if they showed mosaic mRNA expression, transgenic GFP expression and/or $lamc1^{sa379-/-}$ phenotype, depending on the experiment. Experiments were neither randomised nor blinded. All statistical values are displayed as mean ± SD. (Note that some box-and-whisker plots illustrate the median ± minimum and maximum values; this is stated in the figure legends)

Sample sizes, definitions of n, statistical values, statistical tests and p-values are provided in the figure legends in cases where statistical tests have been employed. One exception is analyses of vsx1 differentiation events, which all come from the same dataset and for which sample size is stated in the main text. Sample sizes, definitions of n and statistical values may be provided in the main text for observational data where no statistical test is necessary. Data distribution was assessed before using parametric or non-parametric statistical tests. Statistical significance was considered to be p-value < 0.05. Statistical tests were performed using Prism 7 or Wolfram Mathematica.

Computational Analysis of Simulated and *In Vivo* Data

Changes in dx with Protrusion Length (Figures 7 and S5)

In the main text we have shown that a specific change in the average maximum length reached by protrusions does not lead to the same change in the average distance between sequential events (Figure 7E). A change *dl* in the protrusion length is only expected to lead to a change of 0.22 *dl* in the average value of *dx*. This can be understood as follows. Consider a single differentiating cell which extends a protrusion of length l_{max} and inhibits any cell within a distance l_{max} from differentiating while the protrusion is present as shown in the diagram in Figure S5A.

In the limiting case where the protrusion extends instantaneously the following differentiation event will occur at a distance between $d + l_{max}$ and L from the differentiating cell with equal probability, where L is the maximum distance away from our cell of interest (Figure S5A). It follows that the next differentiation event is expected to occur (on average) at a distance of $d + L + l_{max}$ away from our cell of interest (the mean of a Uniform distribution 2 on the interval $(d + l_{max}, L)$). If we substitute $L = 80$ μm (the maximum dx value in our analysis) and $d = 10$ μm (the average cell diameter) we obtain, $dx = 45 + 0.5 l_{max}$. Assuming that the distribution of sequential differentiation events is stationary (i.e. time independent) it follows that,

$$\overline{dx} = 45 + 0.5\bar{l}_{max}$$

This means that in the limiting case where protrusions extend extremely fast a change in the average protrusion length equal to *dl* will lead to a change in *dx* equal to only 0.5 *dl*.

Now consider a second case where the protrusions extend extremely slowly so they do not effectively inhibit neighbouring cells from differentiating. In this case, neighbouring cells will differentiate anywhere between *d* and *L* away from the differentiating cell and the expected value for *dx* become independent of the protrusions so that,

$$\overline{dx} = 45$$

We expect a real tissue to lie in between these two limiting cases so that,

$$\overline{dx} = 45 + \phi \bar{l}_{max}$$

where φ is a constant between 0 and 0.5 and depends on the timescale of protrusion extension and lateral inhibition relative to the timescale of differentiation (shaded region in Figure S5B).

We can compute φ for our experimental data by substituting $dx = 54.0\mu m$ and $l_{max} = 42.6\ \mu m$ for the wild-type and $dx = 45.3\ \mu m$ and $l_{max} = 12.3\ \mu m$ for the lamc1 mutant. It follows that $\varphi_{WT} = 0.22$ and $\varphi_{lamc1} = 0.024$. The decrease in the slope in the mutant is consistent with a reduced speed in protrusion extension, as observed experimentally.

Pairwise Differences in Space and Time (Figures S6 and S7)

In order to further investigate the coupling between the distance between any two differentiated cells and their time of differentiation we computed the distance in space, Δx (not to be confused with dx which is the distance between sequential differentiation events), and differentiation time, Δt, between all pairs of cells in each experiment and different versions of the theoretical model setup. We then asked how the distributions of Δx and Δt depend on one another.

For experimental data, the distribution of the pairwise position differences Δx for all pairs follows an approximately uniform distribution on the measured interval (Figure S6A). When we restrict this distribution to cells that differentiate within one hour of each other, however, we observe a change in the distribution: very few cells differentiate within less than 30 μm of one another and the distribution of Δx becomes centred around 60μm (Figure S6B). Furthermore, Δx and Δt were negatively correlated (Spearmann's Rho = -0.26; Spearmann's Rank test p-value = $2.2\ 10^{-12}$). We also plotted the mean Δt for cells that differentiated within a specific space interval of one another (Figure S6C). The smaller the distance present up until an interval of 50-60 μm consistent with the average length of protrusions in the between two cells the larger the difference in their time of differentiation. This effect appears to be present up until an interval of 50-60 μm consistent with the average length of protrusions in the wild type.

We repeated the same analysis for the *lamc1* mutant data. We once again found a negative correlation between Δt and Δx (Spearmann's Rho = -0.16; Spearmann's rank test p-value = $4.6\ 10^{-10}$). Furthermore, the distribution of Δx shifts with very few cells differentiating right next to each other. However, the distribution of Δx conditional on $\Delta t < 1$hour is shifted to the left in the lamc1 data when compared to the wild-type experimental data (Figure S6B versus S6E). This is consistent with shorter basal protrusions governing the spatiotemporal dynamics in the *lamc1* mutant. We again plotted the mean Δt for cells that differentiated within a specific space interval of one another (Figure S6F). The smaller the distance between two cells the larger the difference in their time of differentiation. Unlike the wild type data (Figure S6C), this effect is only present up until an interval of 20-30 μm, consistent with a reduced range in lateral inhibition as reflected by the reduction in the protrusion length.

We then turned to pairwise differences for theoretical predictions. We asked whether these observations are consistent with a randomly differentiating tissue or a tissue where basal protrusions mediate lateral inhibition. In a randomly differentiating tissue (where basal protrusions extend but do not signal) we get no correlation between Δt and Δx (Spearmann's Rho = 0.00185; Spearmann's Rank test p-value = 0.502), and conditioning the distribution of Δx on Δt has no impact (Figures S7A–S7C).

On the other hand, simulations where differentiating cells extend signalling protrusions of wild-type length lead to negatively correlated Δt and Δx (Spearmann's Rho = -0.10; Spearmann's Rank test p-value < 10^{-20}) and the distribution of Δx shifts to the right when we condition on Δt much like the experimental data (Figures S6A and S6B versus Figures S7D and S7E). Furthermore, when we plotted the mean Δt for cells that differentiated within a specific space interval of one another we saw similar trends to those observed experimentally (Figure S7F versus Figure S6C).

The same simulations but with short signalling basal protrusions also led to negatively correlated Δt and Δx (Spearmann's Rho = -0.12; Spearmann's Rank test p-value < 10^{-20}), but with a weaker distribution shift for Δx when conditioning on Δt and a reduced range for lateral inhibition as in the experimental data (Figures S6D–S6F versus Figures S7G–S7I). Taken together, these results further support our hypothesis that the spatiotemporal dynamics of neuronal differentiation is contingent upon lateral inhibition mediated by the long and transient basal protrusions we see *in vivo*.

Signalling at Soma-to-Soma (Figures 7 and S8)

Notch signalling typically occurs at soma-to-soma contacts between cells that are direct neighbours of one another (Lai, 2004). We therefore asked if soma-to-soma contacts could also play a role in our system. To investigate this, we run simulations that incorporate lateral inhibition at some-to-soma contacts. This implies non-zero values for α in Equation 3.

When signalling that occurs at all cell contacts is included (i.e. protrusion to soma and soma-to-soma) the predicted distribution differs significantly from that observed experimentally (Figures S8A and S8C; Kolmogorov-Smirnov test, p-value < 10^{-6}). The predicted and observed distributions are even more different when we assume that lateral inhibition is only mediated at soma-to-soma membrane (and not basal protrusions) contacts (Figures S8B and S8C; Kolmogorov Smirnov test, p-value < 10^{-10}). In fact, in this latter case the predicted mean value for dx is below that of a randomly differentiating tissue. This is because signalling taking place only at somal membrane contacts leads to differentiation events that occur in a typical checker-like pattern where cells that are one or two cell diameters apart tend to differentiate at a similar time (Collier et al., 1996; Hadjivasiliou et al., 2016). This can be seen by the peaks at $dx = 30\ \mu m$ in our histograms (Figures S8A and S8B). The absence of such a peak in our experimental data (Figure 6C in the main text) suggests that soma-to-soma contacts play a minimal if any role in the mechanism that determines the pattern of differentiation between spinal neurons.

Sensitivity Analysis of Simulated Data

In this section we discuss the sensitivity of our simulated data and conclusions to variations in key parameters. We specifically explore the sensitivity of our conclusions to variations in parameters that determine the coupling between the basal protrusion growth dynamics and lateral inhibition. Parameters that determine feedback between Notch and Delta signalling have been explored in previous studies and we base our analysis the on these published works (Collier et al., 1996; Cohen et al., 2010).

The simulation of neuronal differentiation in a randomly differentiating tissue is fully independent of any parameters. Our predictions were obtained using a random sampling algorithm on tissues of similar size and structure to the experiments (see Section 1.4). Therefore, our conclusion that the spatiotemporal dynamics observed experimentally are unlikely to come from a randomly differentiating tissue (p-value $< 10^{-10}$ for wild-type data and $< 10^{-6}$ for lamc1 data) is independent of any model parameters.

To explore the dependency between protrusion and differentiation dynamics we varied three key parameters: the Hill exponent q (see Methods Image 4; Equation 4), the speed of the protrusion growth determined by the duration of the protrusions extension period T_{ext}, and the upper limit for the probability of differentiation per simulation step, p. For each variation we run simulations as described in the Methods section and compared the simulated distribution of the distance between sequential differentiation events between the simulations and wild-type experimental data as in the main text using the Kolmogorov-Smirnov test. Methods Figure 4 shows the distributions for different parameters. We found that the comparison between simulation and experiment remains not significant so long as protrusion dynamics and the cellular decision to differentiate are tuned together.

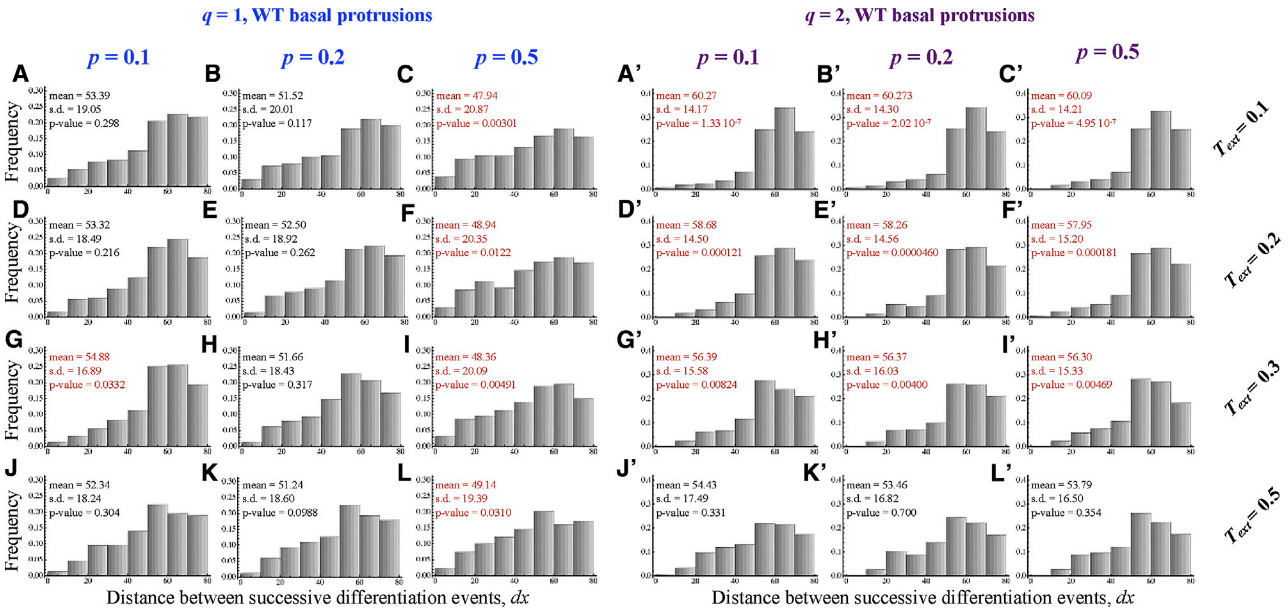

Methods Image 4: Signalling simulations for various values of p, q and T_{ext} when lateral inhibition is mediated only through basal protrusions. Histograms of the distances between successive differentiation events (*dx*) in the simulations. The mean and s.d. of *dx* are shown together with the p-value when the distribution was compared to the wild-type experimental data. Numbers in red indicate a significant deviation from the wild-type experiments. Simulations were repeated 100 times and simulation parameters other than the ones varied in this analysis are given in Table 1.

When the baseline probability of differentiation is very high (Methods Image 4C, F, I, L) or the extending basal protrusions are too fast (Methods Image 4A-C and A'-C') the simulated distribution diverges from the experiment. Very high probability to enter differentiation per time (higher p) leads to a reduction in the average dx. On the other hand, fast basal protrusions together with a high Hill coefficient lead to more narrow dx distributions with larger average dx. Furthermore, higher values for q also lead to more narrow dx distributions for the same values of p and T_{ext} (Methods Image 4A'-L').

However, key features of the simulated distribution remain robust to these variations. In particular, the peak near $dx = 60\mu m$ and the skewed distribution away from small values of *dx* are seen in all our simulations. Hence, this analysis suggests that the exact behaviour of the spatiotemporal dynamics depends on the coupling between the basal protrusion dynamics (actual speed of extension and retraction) and the initiation of cell differentiation as a response to levels of Notch signalling. The same should hold true in a real tissue: a weak dependency of differentiation on the basal protrusion dynamics and lateral inhibition would lead to weaker correlations in spatiotemporal dynamics of neuron differentiation.

We repeated the analysis now allowing lateral inhibition to take place both through basal protrusions and at membrane-membrane contacts. With this combination of signalling, nearly all parameter combinations we tested gave dx distributions that deviate from the

wild-type data (Methods Image 5). When we allowed very weak signalling at soma-to-soma contacts (α = 0.01) some of our simulations were not significantly different from wild-type simulations. Such small values of α lead to membrane-to-membrane signalling is so weak it has a very minor impact on dynamics. These results suggest that soma-to-soma contacts may only contribute very weakly to lateral inhibition prior to protrusion extension.

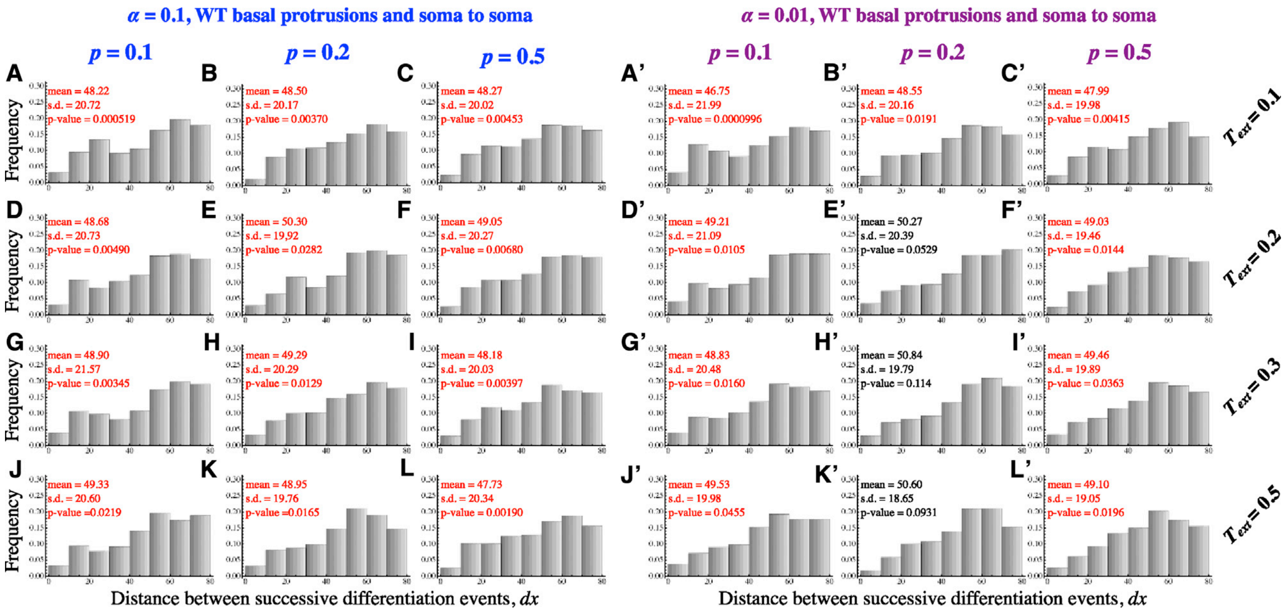

Methods Image 5: Signalling simulations for various values of *p*, *a* and T_{ext} when lateral inhibition is mediated only through basal protrusions and soma-to-soma contacts. Histograms of the distances between successive differentiation events (*dx*) in the simulations. The mean and s.d. of *dx* are shown together with the p-value when the distribution was compared to the wild-type data. Numbers in red indicate a significant deviation from the wild-type experiments. Simulations were repeated 100 times and simulation parameters other than the ones varied in this analysis are given in Methods Table 1.

When cells only signal at their soma contacts the basal protrusion dynamics do not matter. To explore whether soma-to-soma only signalling could lead to wild-type-like distributions we run simulations varying *p* and α. None of the simulated distributions were close to resembling the wild-type experimental data (Methods Image 6). As in the main text, we find a bias for *dx* between 20 μm to 30 μm, the typical distance between cells that are two to three membranes apart. Weaker soma signalling (reducing α to 0.01) led to distributions more similar to those of a randomly differentiating tissue. Therefore, our analysis suggests that the observed dynamics are unlikely to be due to lateral inhibition mediated at soma-to-soma contacts alone

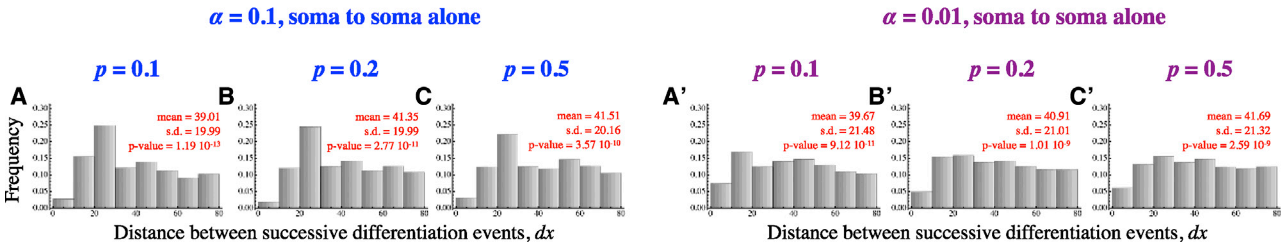

Methods Image 6: Soma-to-soma only signalling simulations for varying *p* and *a* when lateral inhibition takes place only at soma-to-soma contacts. Histograms of the distances between successive differentiation events (dx) in the simulations. The mean and s.d. of *dx* are shown together with the p-value when the distribution was compared to the wild-type experimental data. Numbers in red indicate a significant deviation from the wild-type experiments. Simulations were repeated 100 times and simulation parameters other than the ones varied in this analysis are given in Methods Table 1.

Developmental Cell, Volume *49*

Supplemental Information

Basal Protrusions Mediate Spatiotemporal Patterns of Spinal Neuron Differentiation

Zena Hadjivasiliou, Rachel E. Moore, Rebecca McIntosh, Gabriel L. Galea, Jonathan D.W. Clarke, and Paula Alexandre

Figure S1

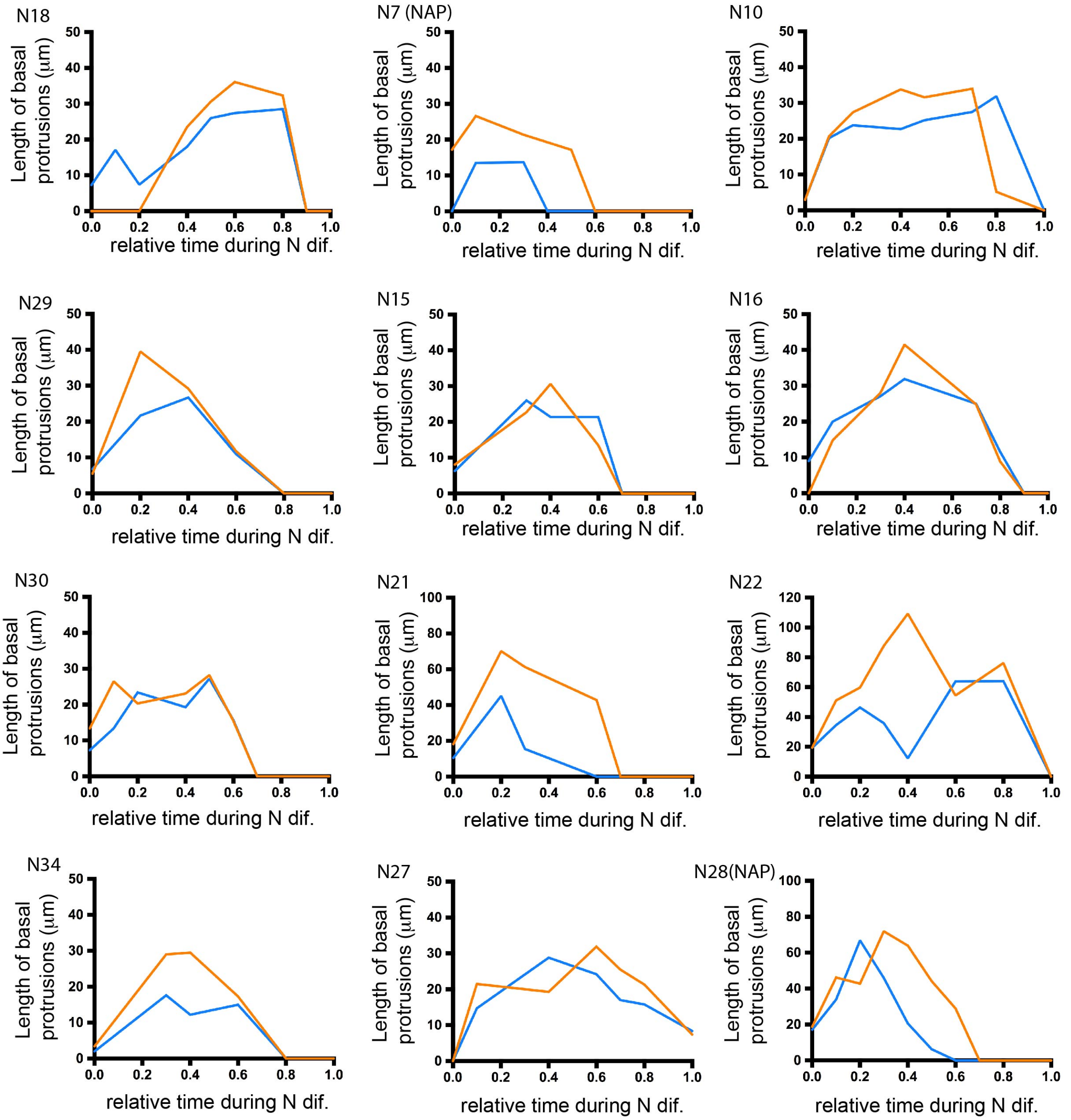

The dynamics of basal protrusions growth during neuronal differentiation (related to Figure 1).

Graphs showing the change in length over time of the left (orange) and right (blue) basal protrusions from individual non-apical progenitors (NAPs) and individual differentiating neurons. The time has been normalised from (0), the moment in which differentiating neurons begin elongation of basal protrusions, to (1), when neurons initiate axon formation.

Figure S2

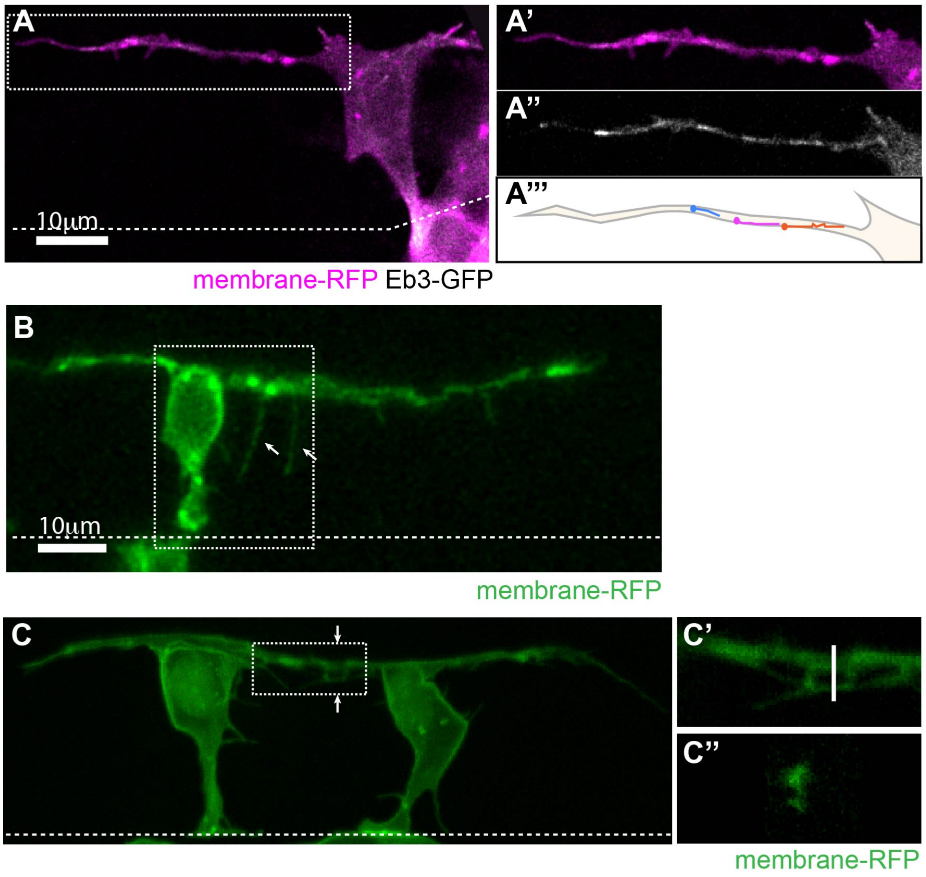

Basal protrusions contain dynamic microtubules, form filopodia and can overlap (related to Figure 1).

A) T-shaped differentiating neuron with a long basal protrusion. White box marks the area of higher magnification shown in A'-A'''). A') Membrane-RFP. A'') EB3-GFP (Movie 2). A''') Illustration of EB3-GFP comet trajectories during a 30 second time-lapse period.

B) T-shaped cell with filopodia (arrows) on basal protrusion (Movie 3).

C) Two neurons differentiating 18μm apart with overlapping basal arms. C') Higher magnification of white box in C). C'') Cross-section of the basal protrusions at the line shown in C').

All images are projected images from confocal z-stacks. Dashed line shows the apical surface.

Figure S3

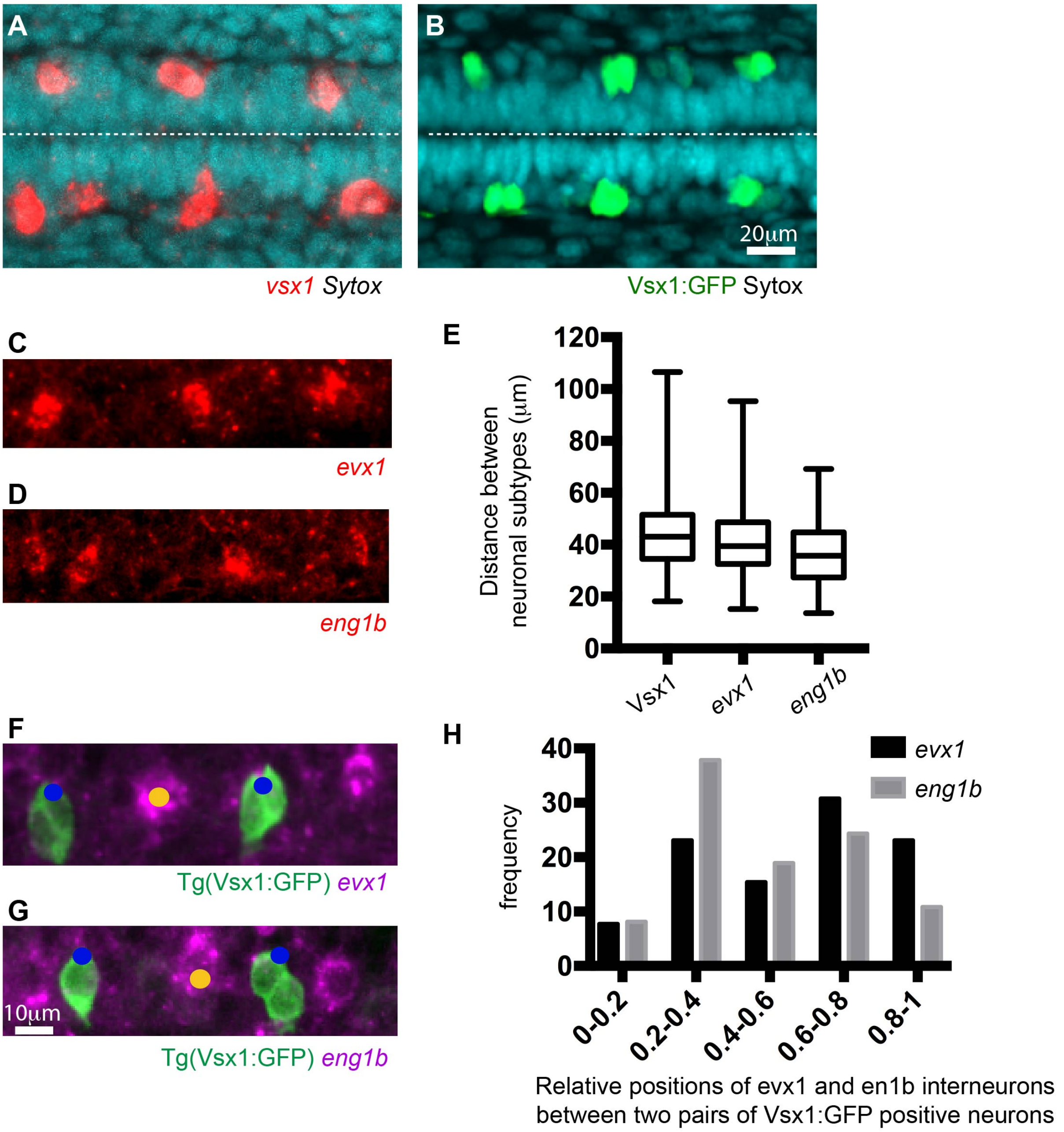

Neuronal spacing pattern in the zebrafish spinal cord (related to Figure 4).

A) and B) Dorsal views of Vsx1 expressing cells revealed by in situ hybridization (A) and Vsx1:GFP transgene expression (B) at 20 hfp. Dashed line shows the apical surface.

C) and D) Lateral views of evx1 (C) and eng1b (D) expressing spinal neurons revealed by in situ hybridization at 20hpf.

E) Box-and-whisker plot showing the distance between neurons of the same subtype in the most caudal spinal region at 20hpf (mean ± SD; Vsx1:GFP: 44.4 ± 14.1 μm,

n=18; evx1: 41 ± 15.4 μm, n=7; eng1b: 35.7 ± 13.3 μm, n=5 embryos). The line inside the box represents the median and whiskers represent minimum and maximum values. Data analysed using Kruskal-Wallis with Dunn's multiple comparison test (non-significant).

F and G) Dorsolateral views showing the relative positions of Vsx1:GFP and evx1 (F) or eng1b (G) expressing cells revealed by in situ hybridisation.

H) Frequency distribution chart shows the relative positions of evx1 and en1b expressing interneurons between two pairs of Vsx1:GFP expressing neurons. The distance between the two pairs of Vsx1:GFP neurons has been normalised from 0 (anterior) to 1 (posterior). All evx1 and eng1b expressing neurons that shared the same position with a Vsx1 neuron have been included in position (1).

Figure S4

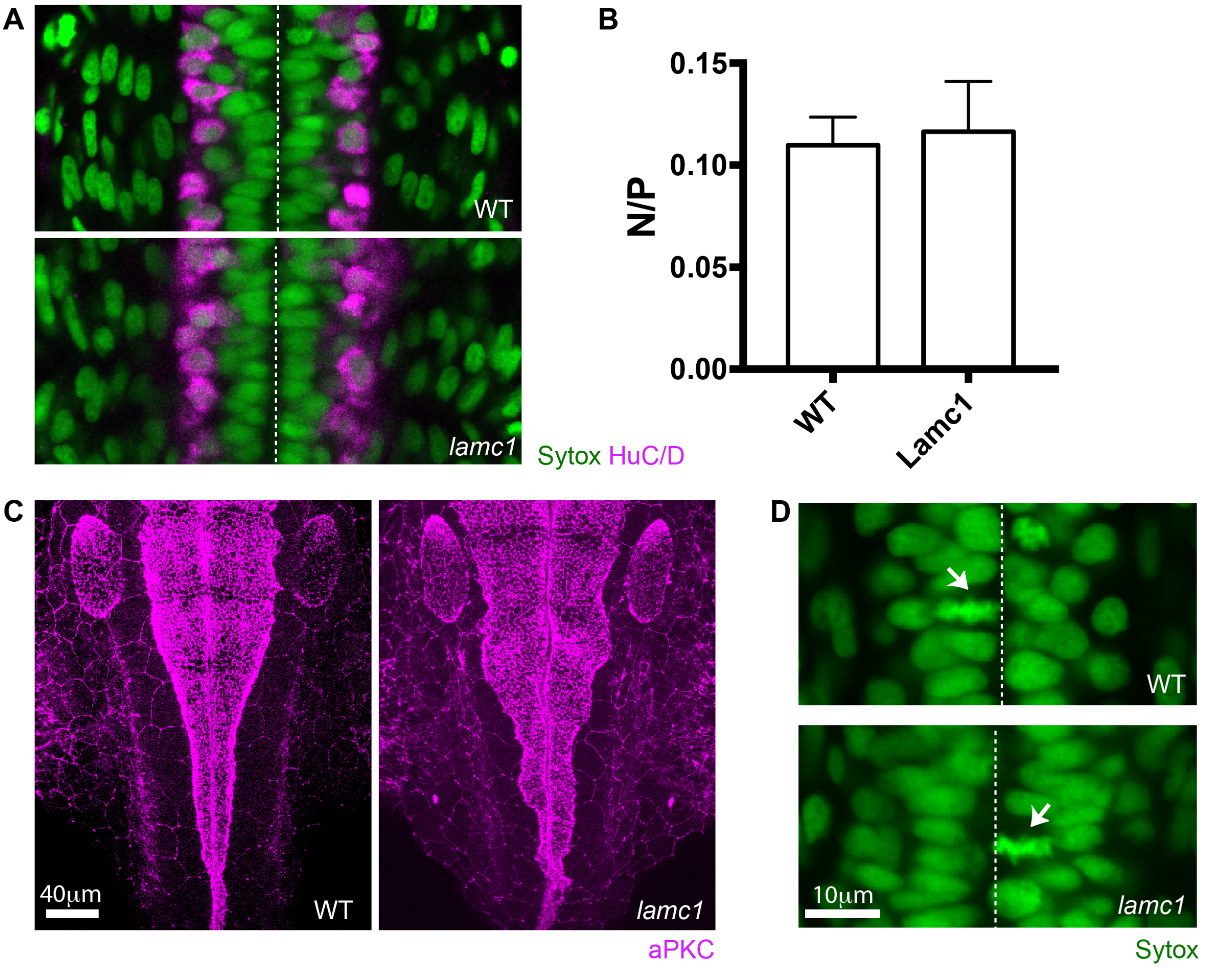

***Lamc1* mutant characterization (related to Figure 6).**

A) Dorsal view of spinal cord in wild type and *lamC1* mutant embryos. HuC/D immunoreactivity (magenta) reveals the position of spinal cord neurons at 24 hpf.

B) Graph showing the ratio of neurons to progenitors in wild type and *lamC1* mutant spinal cords at 24 hpf (mean ± SD, 0.11 ± 0.01 for wild type and 0.12 ± 0.02 for *lamc1* mutant, unpaired one-tailed t-test p=0.4428).

C) Dorsal view of wild type and *lamC1* mutant embryos at the hindbrain. aPKC immunoreactivity (magenta) shows the position of the apical surfaces at 28 hpf.

D) Dorsal view of spinal cord in wild type and *lamC1* mutant embryos showing apical mitoses (arrows).

Dashed line shows position of the apical surfaces in A) and D). Nuclei labelled by Sytox in A) and D).

Figure S5

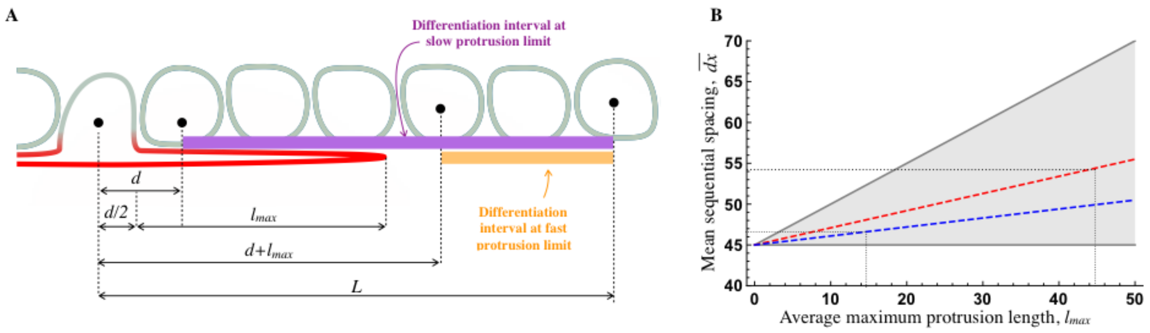

C

$\bar{dx} = \frac{L+d}{2} + 0.5\bar{l}_{max}$	Expected mean sequential spacing at fast protrusion limit.
$\bar{dx} = \frac{L+d}{2}$	Expected mean sequential spacing at slow protrusion limit.
$\bar{dx} = \frac{L+d}{2} + \phi\bar{l}_{max}, \quad 0 < \phi < 0.5$	Expected mean sequential spacing for protrusions observed experimentally.

The average distance between differentiation events depends linearly on the average maximum length reached by protrusions (related to Figure 7 and STAR methods section - Computational analysis of simulated and *in vivo* data).

A: The region where the next differentiation event is expected to occur following protrusion extension. Two limiting cases are illustrated: one where protrusion extension is slow and inefficient and so the subsequent differentiation event is expected to occur anywhere between distance *d* and *L* from our cell of interest. This leads to an expected mean for the sequential spacing, $\bar{dx}$, given by $\bar{dx} = \frac{L+d}{2}$. The second case illustrated represents instantaneous protrusion extension and immediate lateral inhibition. In this case the subsequent differentiation event is expected to occur anywhere between distance $d/2$ + l_{max} and L. This leads to an expected mean for the sequential spacing given by $\bar{dx} = \frac{L+d}{2} + 0.5\bar{l}_{max}$.

B: The expected relationship between the average distance between sequential events $\bar{dx}$ and the average maximum protrusion length l_{max} for the wild type (red)

and *lamc1* mutant (blue).

C: We expect the mean sequential spacing in a real tissue to relate to the protrusion maximum length by $\bar{dx} = \frac{L+d}{2} + \phi \bar{l}_{max}$ where 0 < ϕ < 0.5. The value of ϕ captures the speed and efficiency of protrusion extension and signaling. We computed ϕ in the wild type and *lamc1* mutant to be equal to ϕ_{WT} = 0.22 and ϕ_{lamc1} = 0.024. The decrease in the slope in the mutant is consistent with a reduced speed in protrusion extension, as observed experimentally. More details can be found under the Quantification and Statistical Analysis section in the STAR Methods.

Figure S6

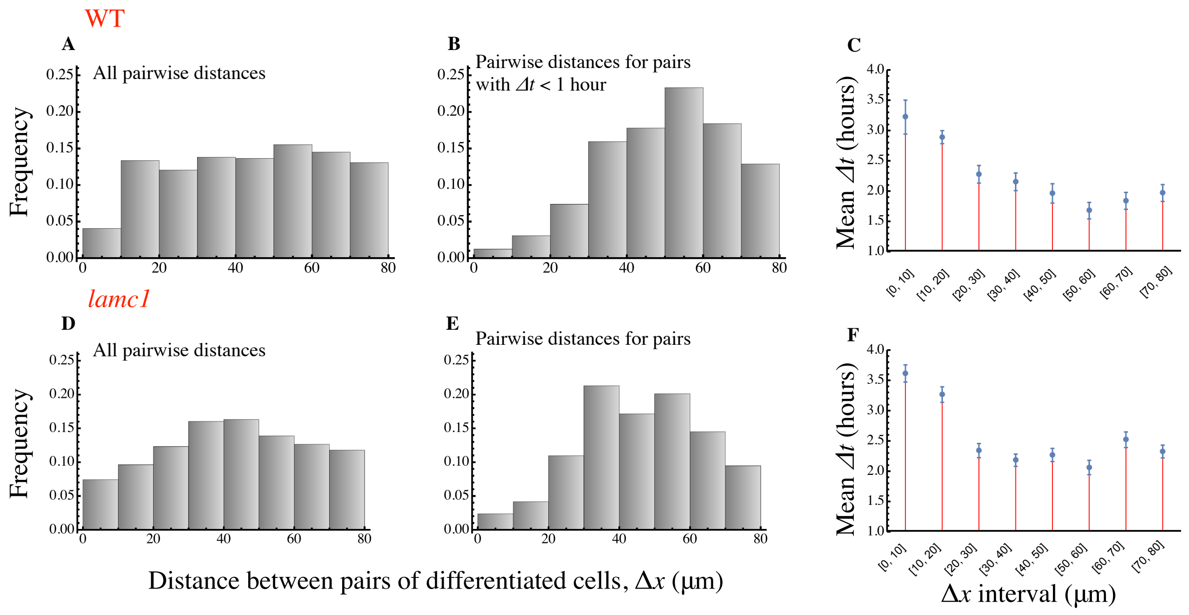

Neurons that are born closer in time tend to differentiate further apart in space (related to STAR methods section - Computational analysis of simulated and *in vivo* data).

Distribution of pairwise distances between all differentiating neurons (A for experimental wild type and D for *lamc1* mutant) and neurons that differentiated less than 1 hour apart from one another (B for experimental wild type and E for *lamc1* mutant).

C,F) show the mean pairwise time difference in the time of differentiation (Δt) for varying intervals of pairwise spatial differences Δx for the wild type (C) and *lamc1* mutant (F). All plots were produced using all measured pairs that satisfied $x < 80\mu m$.

Figure S7

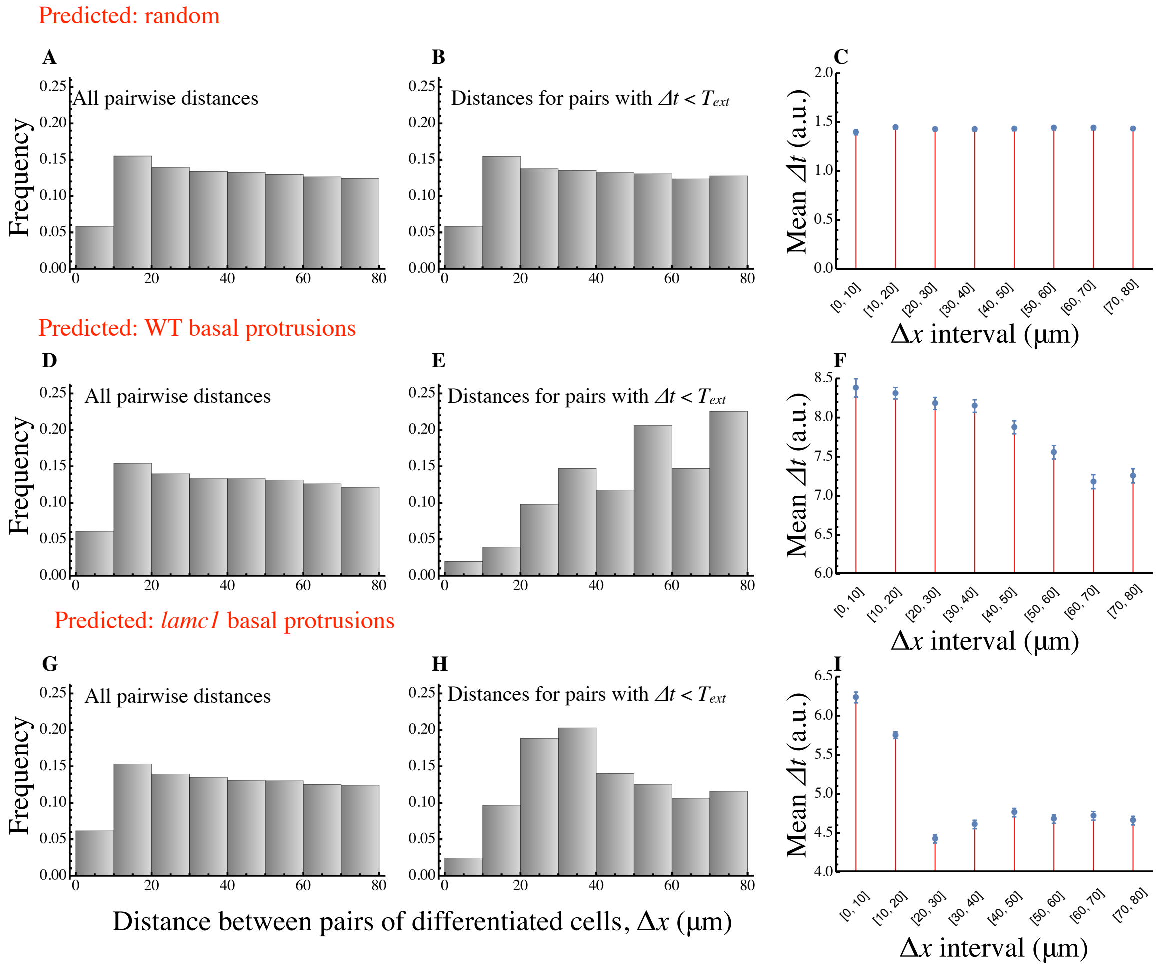

Theoretical model supports the role of basal protrusions in patterning spatiotemporal dynamics of neuron differentiation (related to STAR methods section - Computational analysis of simulated and *in vivo* data).

Distribution of pairwise distances between all differentiating neurons (A. for random differentiation, D. for wild type basal protrusions and G. for short basal protrusions) and neurons that differentiated less than Text time apart from one another in the simulations (B. for random differentiation, E. for wild type basal protrusions and H. for short basal protrusions).

C, F, I) show the mean pairwise time difference in the time of differentiation (Δt) for varying intervals of pairwise spatial differences Δx for a randomly differentiating tissue (C), a tissue where wild type basal protrusions mediate lateral inhibition (F) and a tissue where lamc1 like protrusions mediate lateral inhibition (I). Plots were produced using all simulated pairs that satisfied x < 80µm. Parameters used for the simulations are outlined in Table 1.

Figure S8

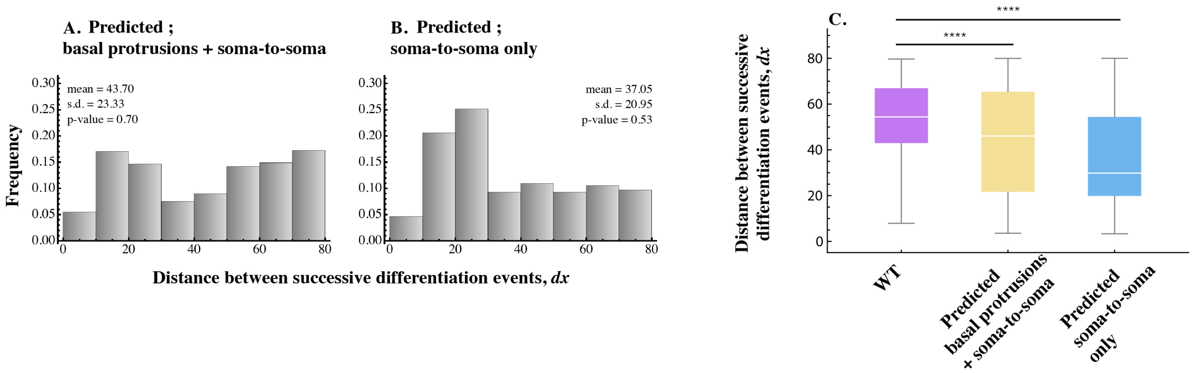

Soma-to-soma signaling does not explain experimentally observed distributions (related to STAR methods section - Computational analysis of simulated and *in vivo* data).

Predicted distributions of the distance between sequential events when wild type basal protrusions and soma-to-soma contacts mediate lateral inhibition (A) and when only soma-to-soma contacts mediate lateral inhibition (B). C) shows the box plots of the wild type data alongside the data shown in A) and B). The simulated distributions considering soma-to-soma signaling do not match experimental observations.

Kolmogorov-Smirnov test comparing wild-type and predicted distribution when protrusion and soma-to-soma signaling was assumed gave a p-value of 1.7 10^{-8}.

Kolmogorov-Smirnov test comparing wild-type and predicted distribution when only soma-to-soma signaling was assumed gave a p-value < 10^{-10}.

Supplemental data 1

Spatiotemporal pattern diagrams illustrating Vsx1:GFP neuronal differentiation events in time and space in the developing zebrafish spinal cord of wild type and *lamc1* mutant embryos.

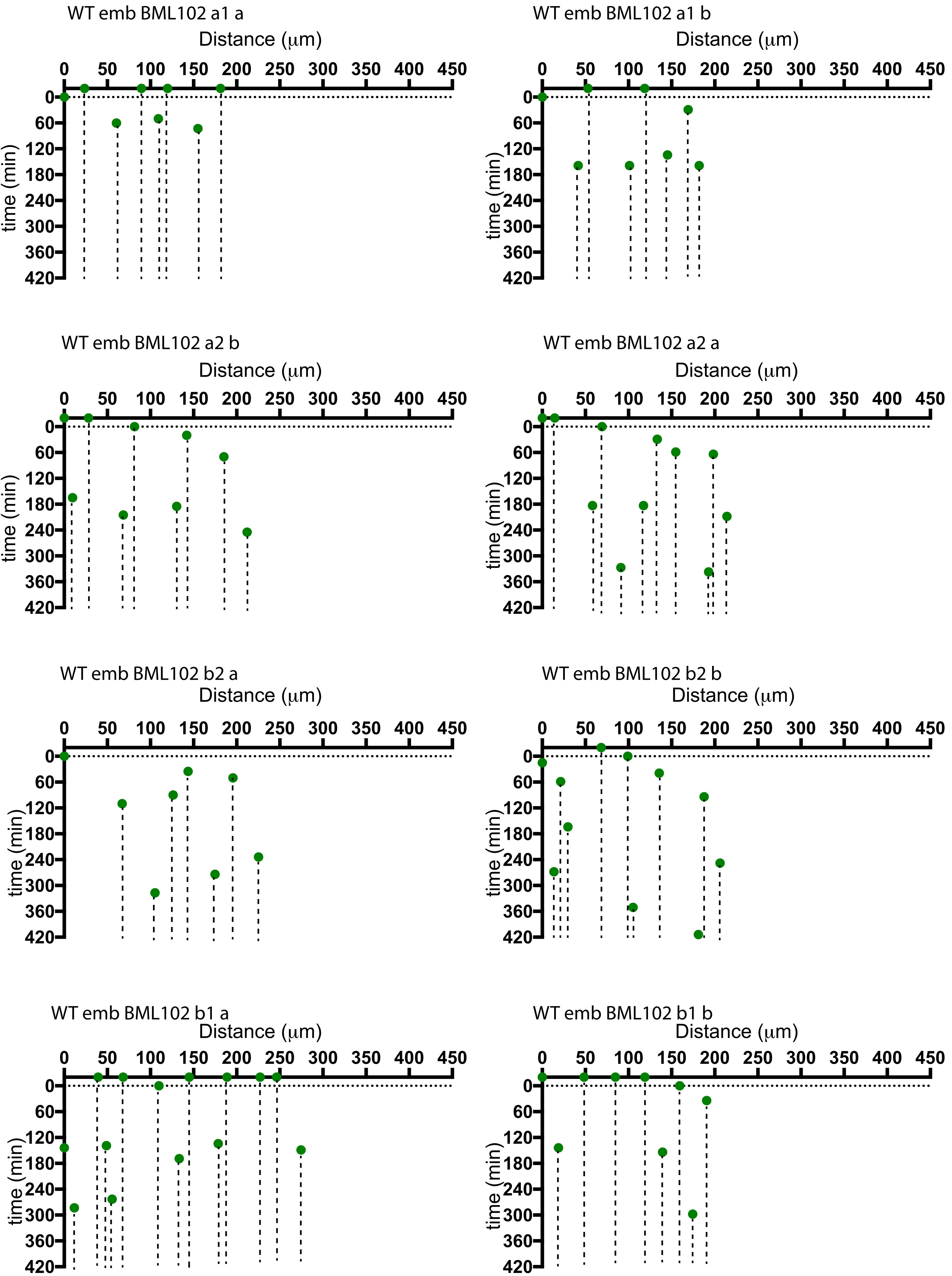
WT emb BML102 a1 a
WT emb BML102 a1 b
WT emb BML102 a2 b
WT emb BML102 a2 a
WT emb BML102 b2 a
WT emb BML102 b2 b
WT emb BML102 b1 a
WT emb BML102 b1 b
Distance (μm)
0 50 100 150 200 250 300 350 400 450
time (min)
0 60 120 180 240 300 360 420

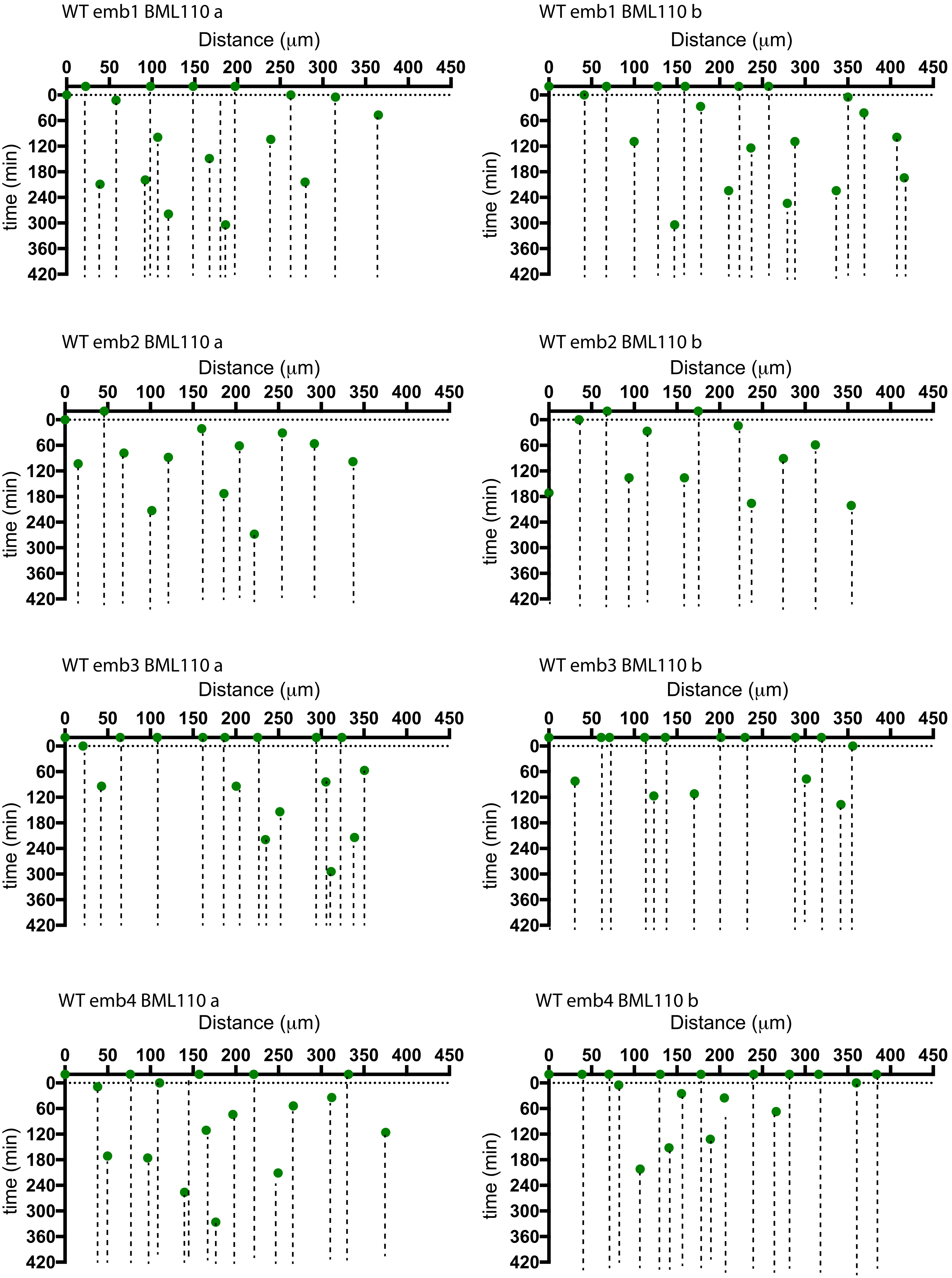

WT emb1 BML110 a
WT emb1 BML110 b
WT emb2 BML110 a
WT emb2 BML110 b
WT emb3 BML110 a
WT emb3 BML110 b
WT emb4 BML110 a
WT emb4 BML110 b
Distance (μm)
0 50 100 150 200 250 300 350 400 450
time (min)
0 60 120 180 240 300 360 420

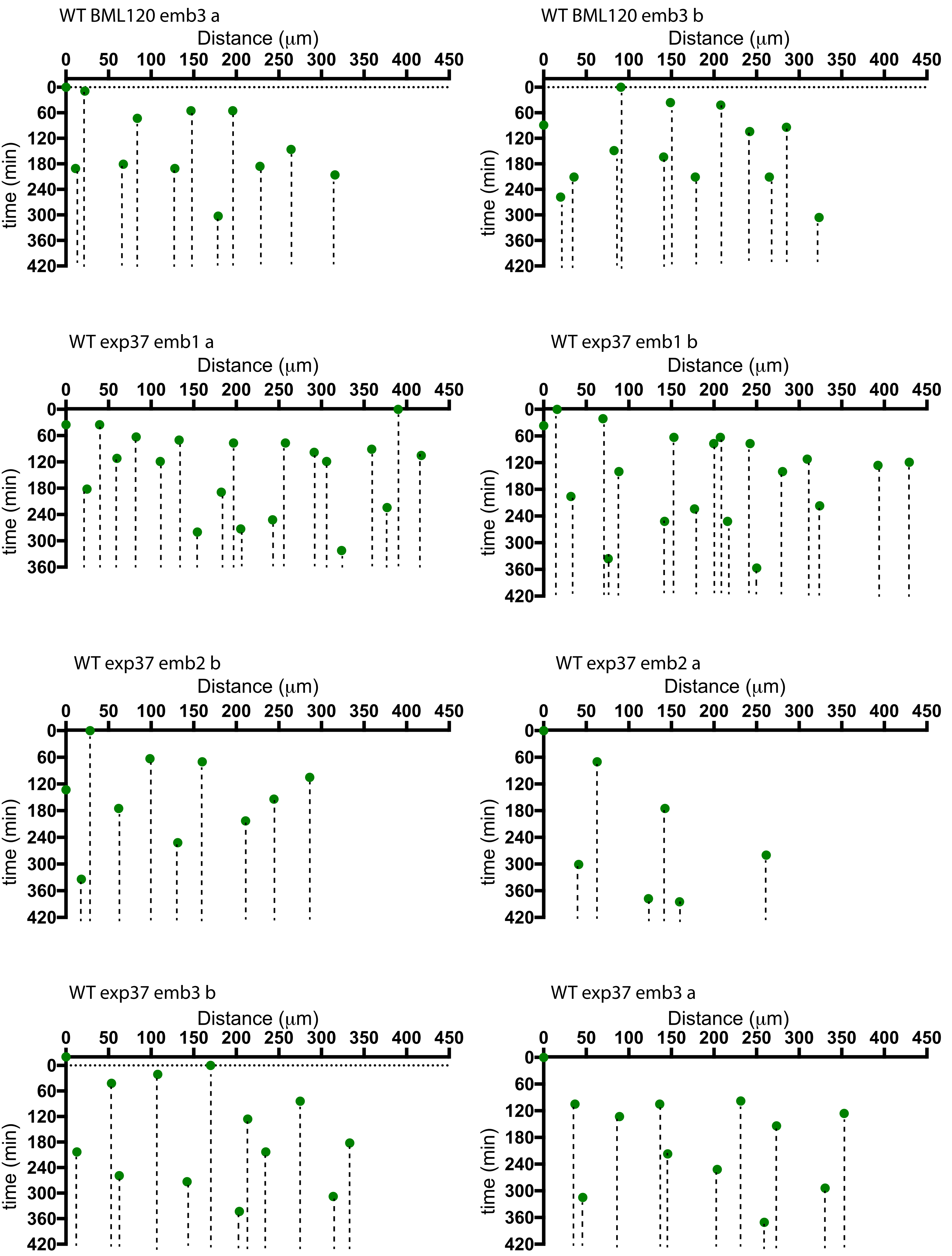
WT BML120 emb3 a
Distance (μm)
0 50 100 150 200 250 300 350 400 450
time (min)
0 60 120 180 240 300 360 420
WT BML120 emb3 b
Distance (μm)
0 50 100 150 200 250 300 350 400 450
time (min)
0 60 120 180 240 300 360 420
WT exp37 emb1 a
Distance (μm)
0 50 100 150 200 250 300 350 400 450
time (min)
0 60 120 180 240 300 360
WT exp37 emb1 b
Distance (μm)
0 50 100 150 200 250 300 350 400 450
time (min)
0 60 120 180 240 300 360 420
WT exp37 emb2 b
Distance (μm)
0 50 100 150 200 250 300 350 400 450
time (min)
0 60 120 180 240 300 360 420
WT exp37 emb2 a
Distance (μm)
0 50 100 150 200 250 300 350 400 450
time (min)
0 60 120 180 240 300 360 420
WT exp37 emb3 b
Distance (μm)
0 50 100 150 200 250 300 350 400 450
time (min)
0 60 120 180 240 300 360 420
WT exp37 emb3 a
Distance (μm)
0 50 100 150 200 250 300 350 400 450
time (min)
0 60 120 180 240 300 360 420

WT BML120 emb1 b

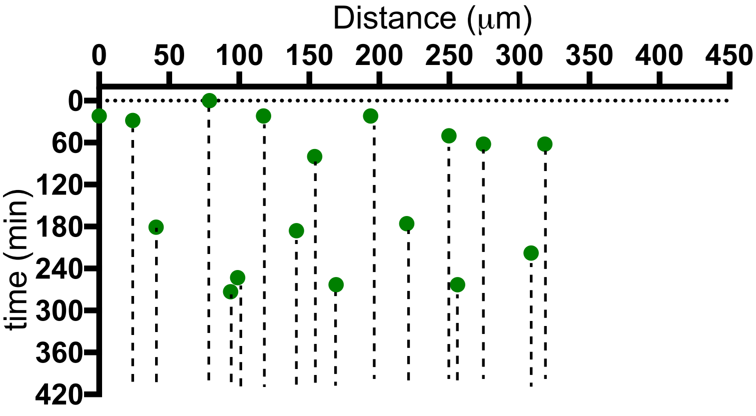

WT BML120 emb1 a

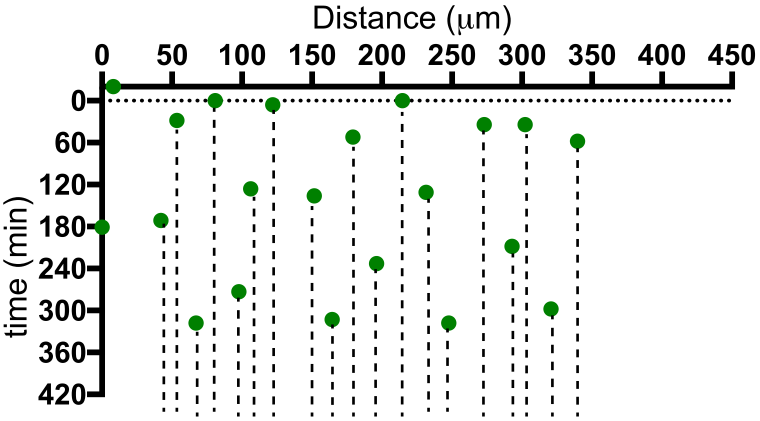

WT BML120 emb2 b

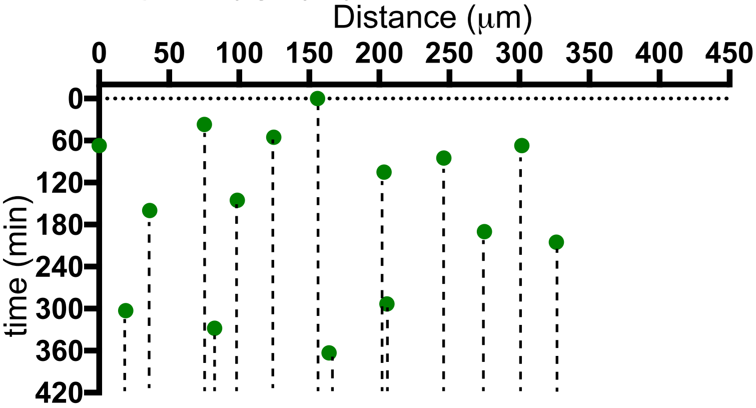

WT BML120 emb2 a

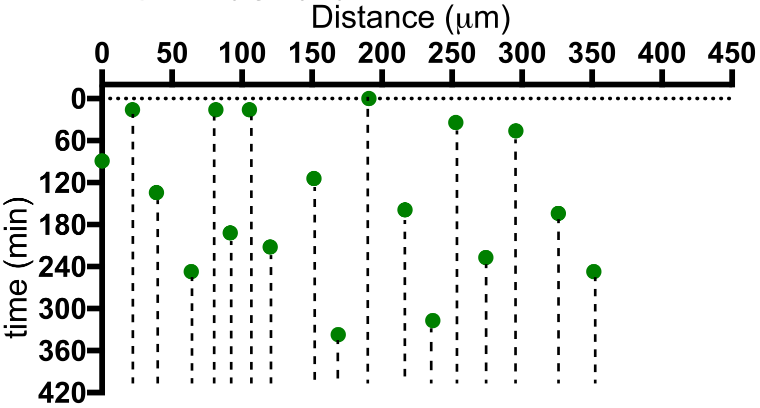

Lamc1 mut 5 R458 b

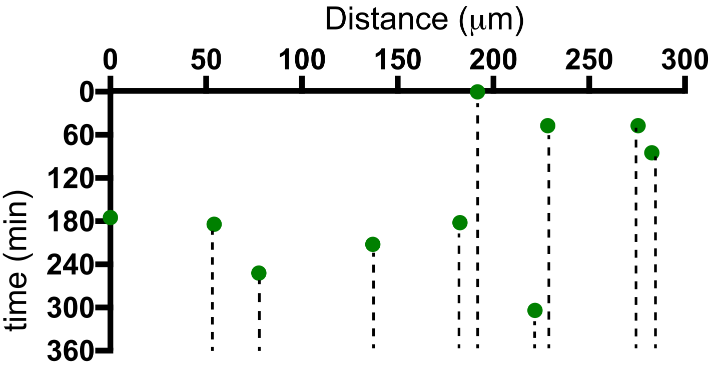

Lamc1 mut 5 R458 a

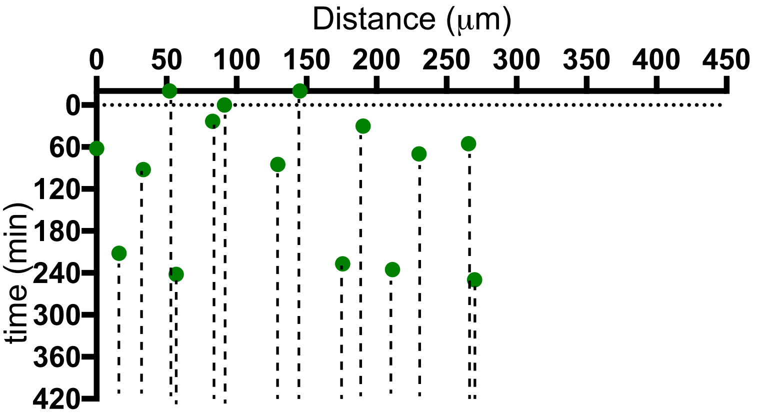

Lamc1 mut emb6 R458 b

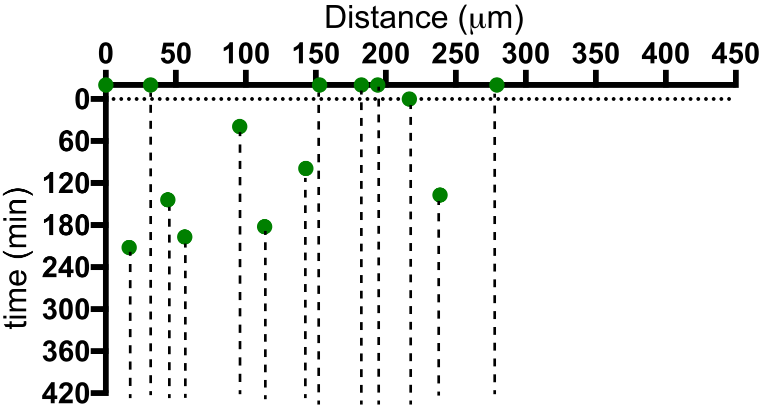

Lamc1 mut emb6 R458 a

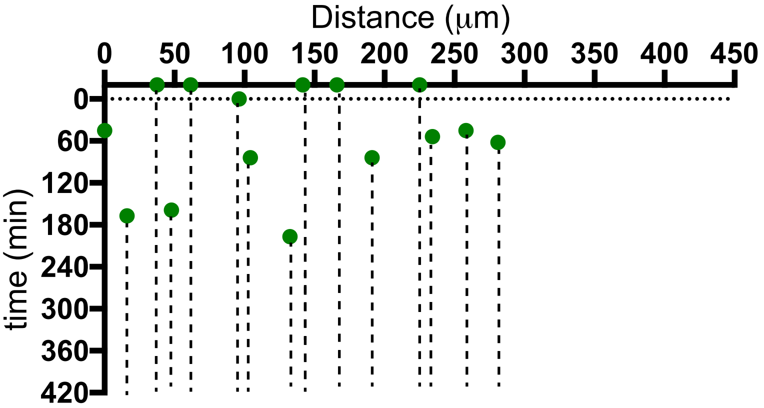

Lamc1 mut emb7 R458 b

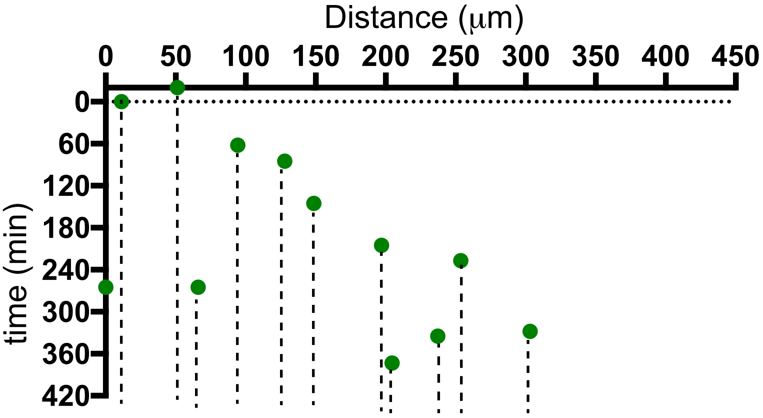

Lamc1 mut emb7 R458 a

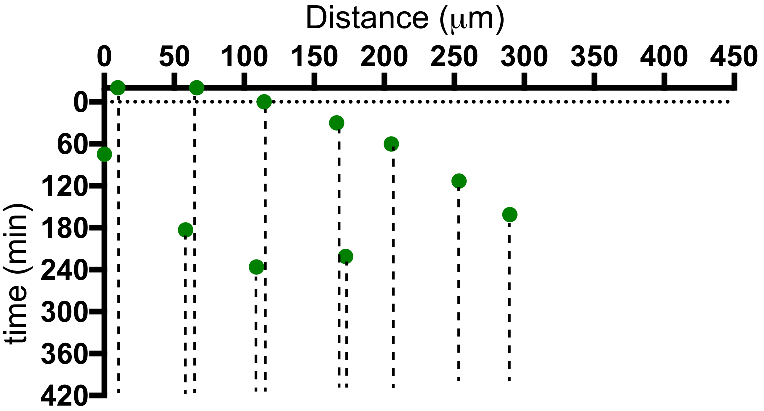

Lamc1 mut emb8 R458 b

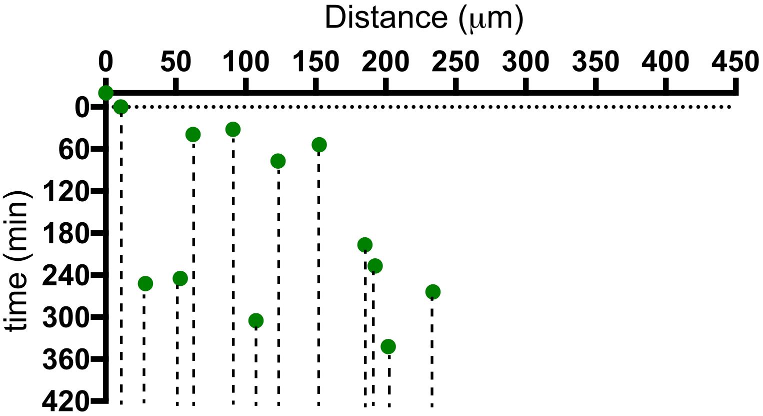

Lamc1 mut emb8 R458 a

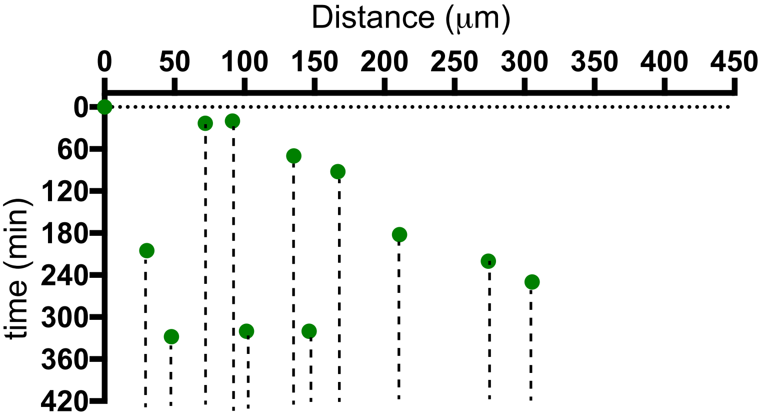

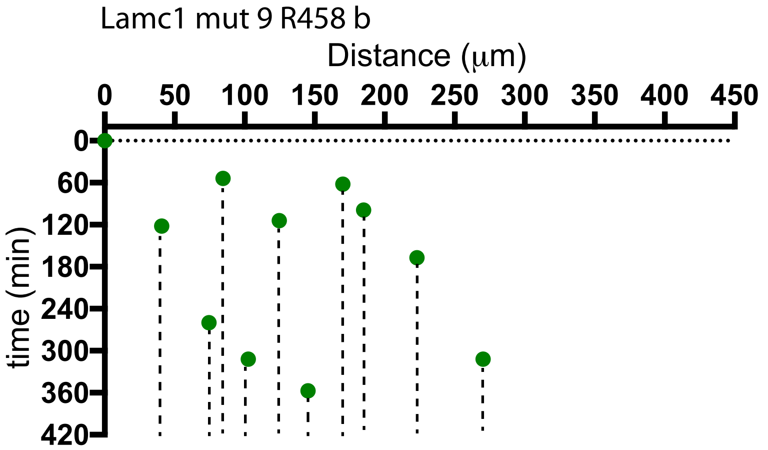
Lamc1 mut 9 R458 b
Distance (µm)
time (min)

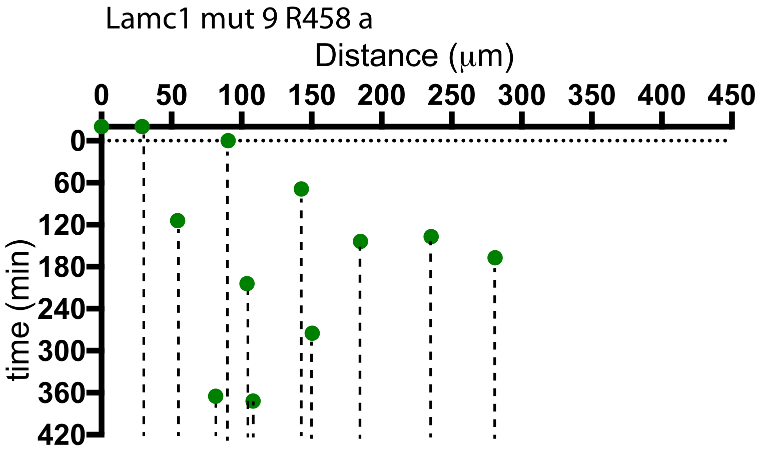
Lamc1 mut 9 R458 a
Distance (µm)
time (min)

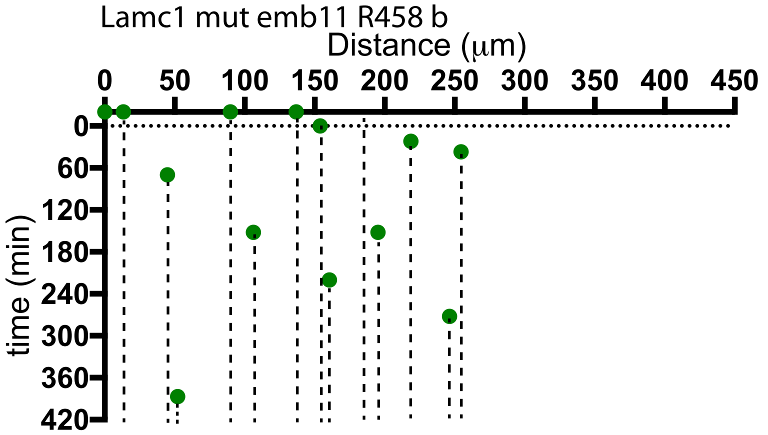
Lamc1 mut emb11 R458 b
Distance (µm)
time (min)

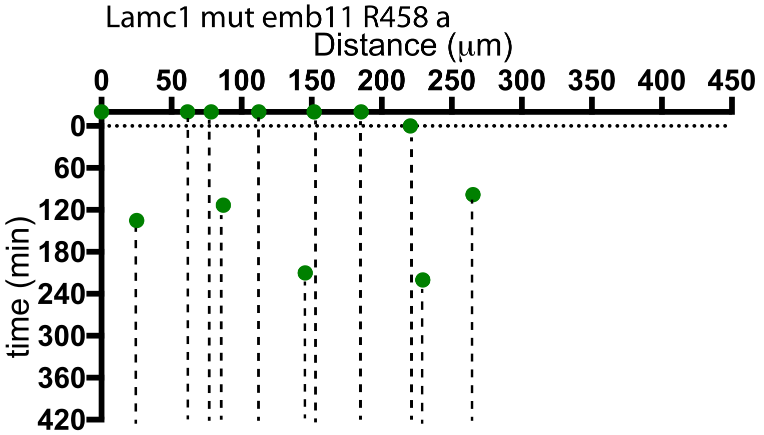
Lamc1 mut emb11 R458 a
Distance (µm)
time (min)

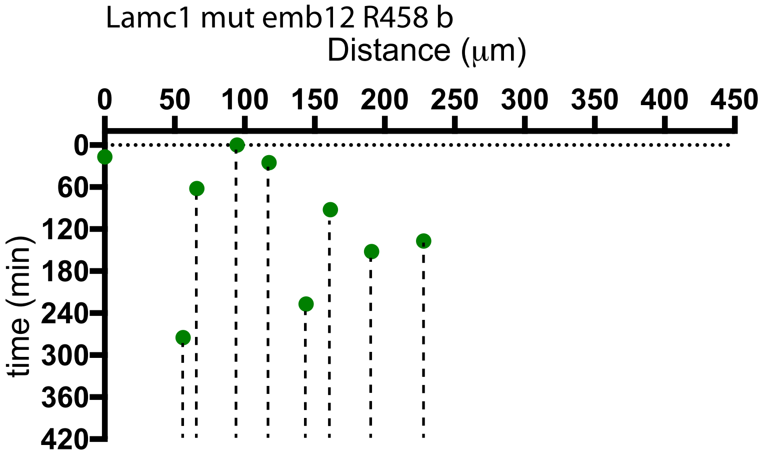
Lamc1 mut emb12 R458 b
Distance (µm)
time (min)

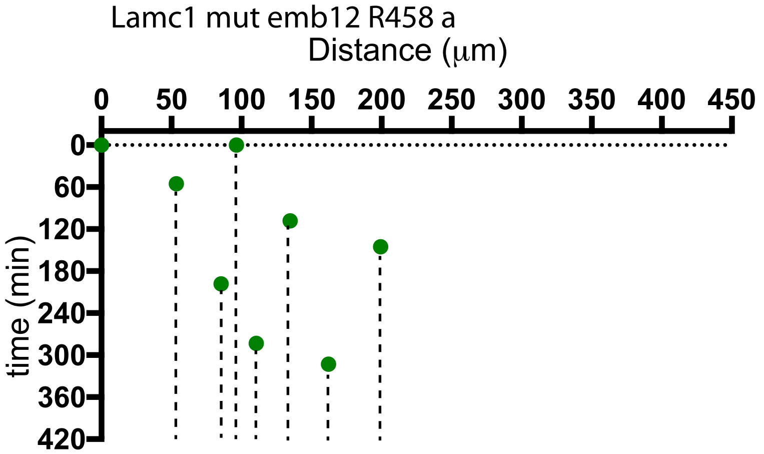
Lamc1 mut emb12 R458 a
Distance (µm)
time (min)

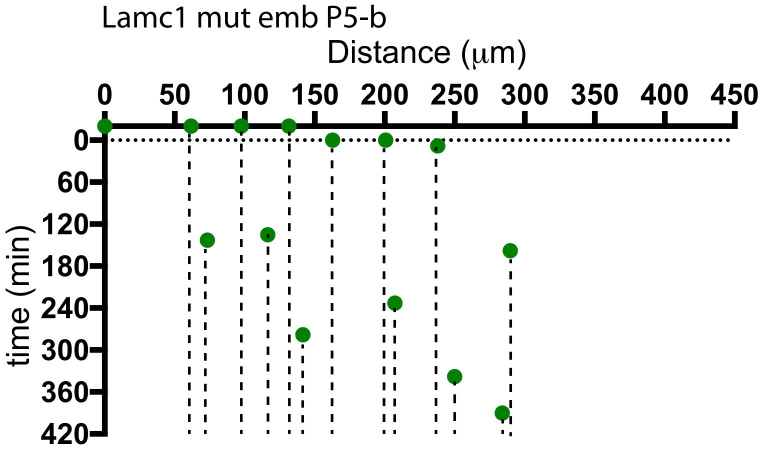
Lamc1 mut emb P5-b
Distance (µm)
time (min)

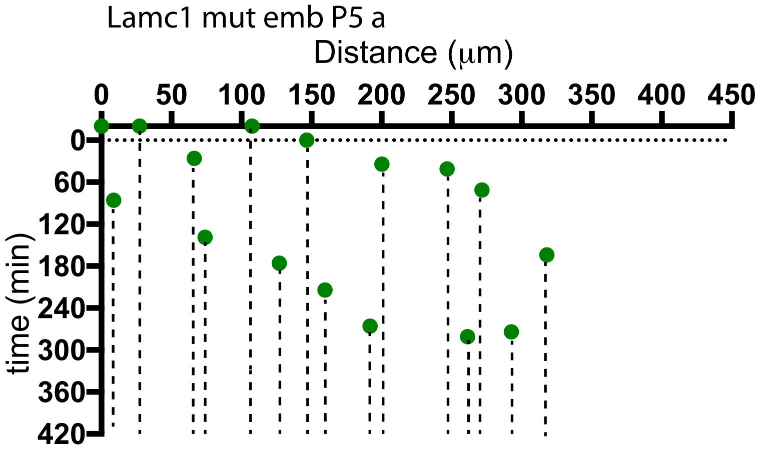
Lamc1 mut emb P5 a
Distance (µm)
time (min)

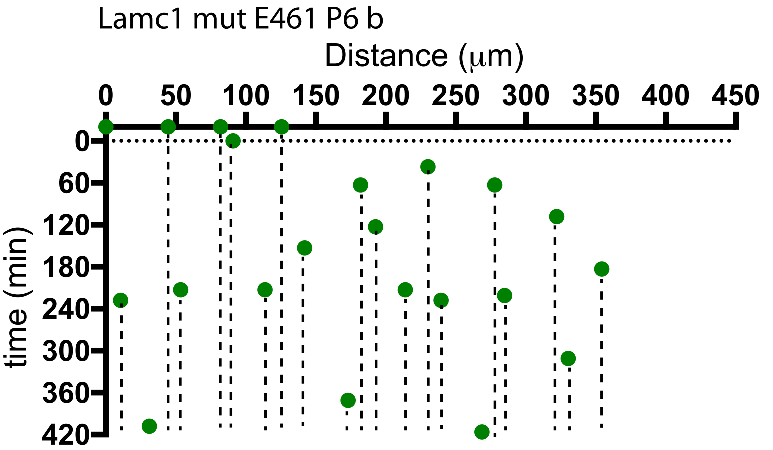
Lamc1 mut E461 P6 b
Distance (μm)
0 50 100 150 200 250 300 350 400 450
time (min)
0 60 120 180 240 300 360 420

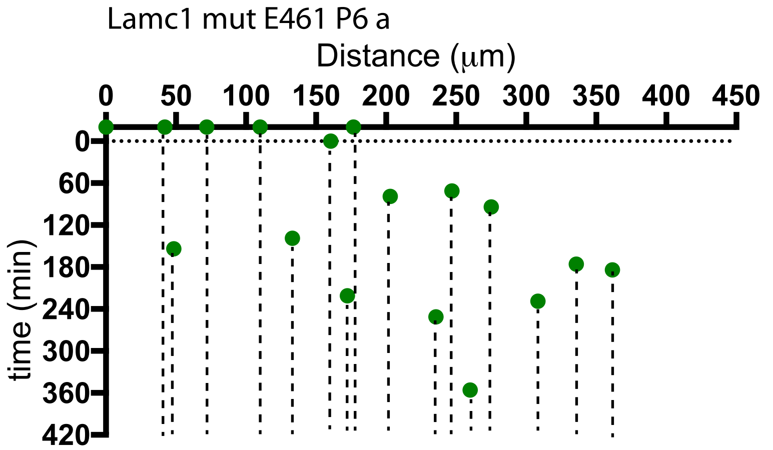
Lamc1 mut E461 P6 a
Distance (μm)
0 50 100 150 200 250 300 350 400 450
time (min)
0 60 120 180 240 300 360 420

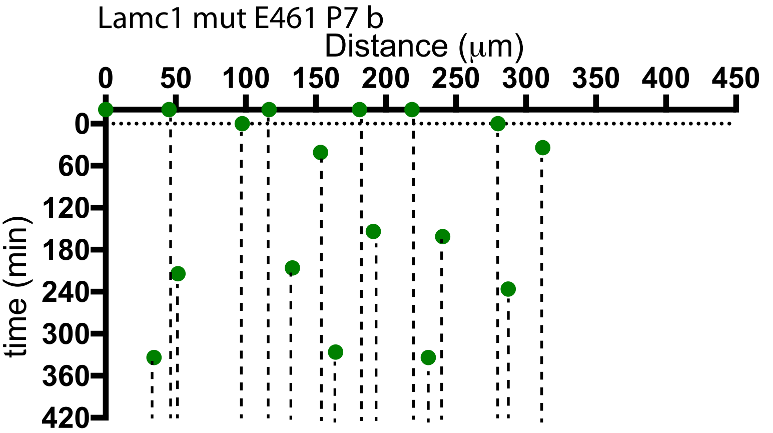
Lamc1 mut E461 P7 b
Distance (μm)
0 50 100 150 200 250 300 350 400 450
time (min)
0 60 120 180 240 300 360 420

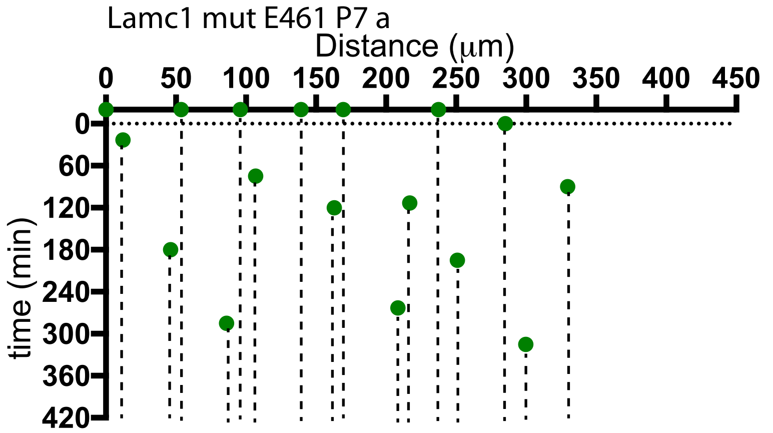
Lamc1 mut E461 P7 a
Distance (μm)
0 50 100 150 200 250 300 350 400 450
time (min)
0 60 120 180 240 300 360 420

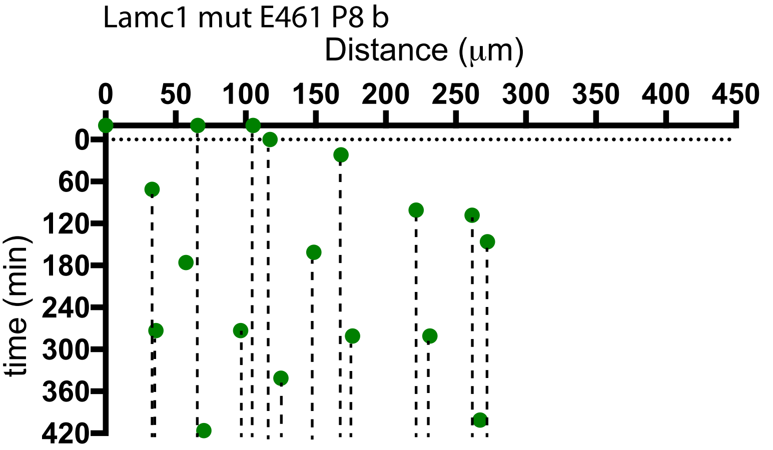
Lamc1 mut E461 P8 b
Distance (μm)
0 50 100 150 200 250 300 350 400 450
time (min)
0 60 120 180 240 300 360 420

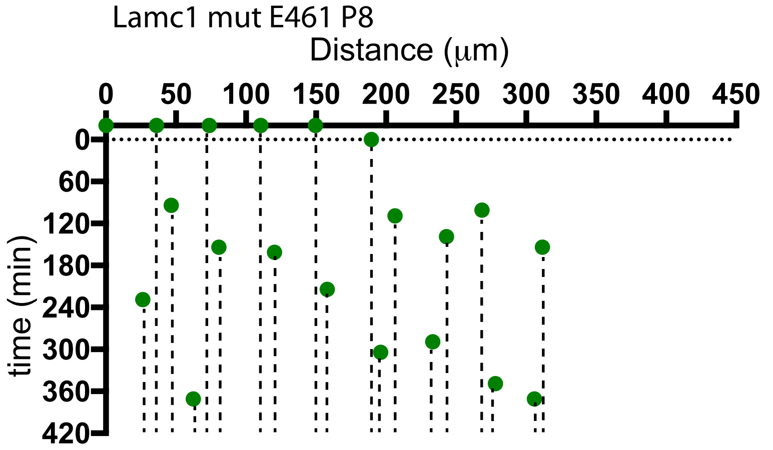
Lamc1 mut E461 P8
Distance (μm)
0 50 100 150 200 250 300 350 400 450
time (min)
0 60 120 180 240 300 360 420

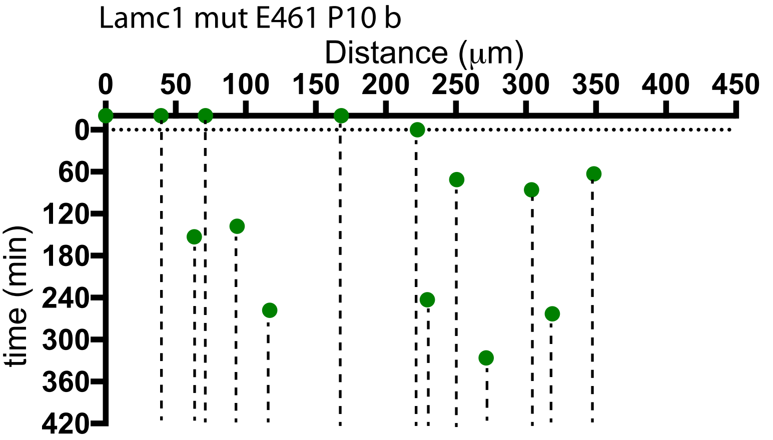
Lamc1 mut E461 P10 b
Distance (μm)
0 50 100 150 200 250 300 350 400 450
time (min)
0 60 120 180 240 300 360 420

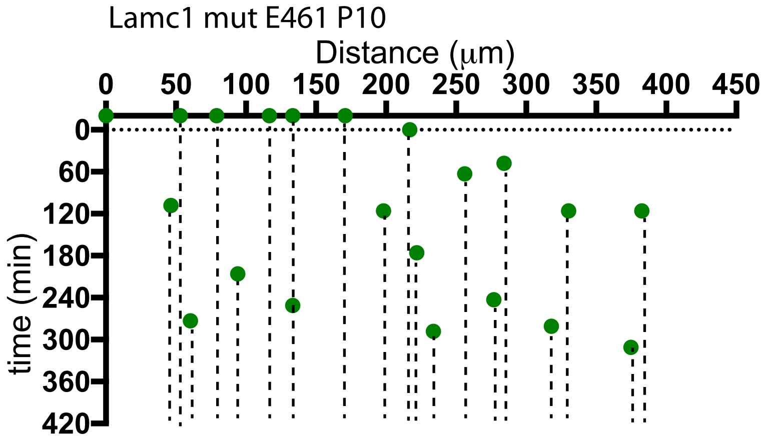
Lamc1 mut E461 P10
Distance (μm)
0 50 100 150 200 250 300 350 400 450
time (min)
0 60 120 180 240 300 360 420

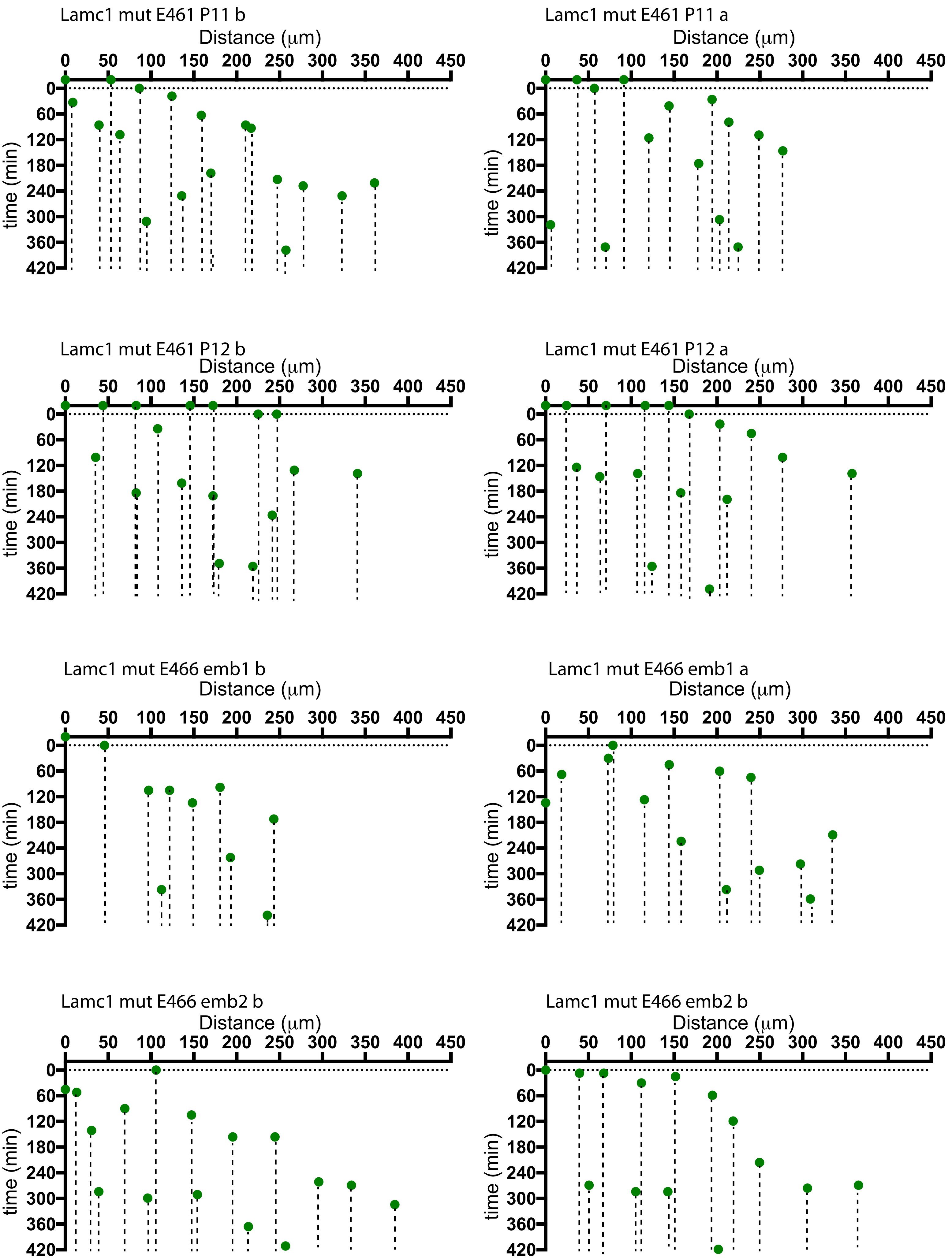

Lamc1 mut E461 P11 b
Lamc1 mut E461 P11 a
Lamc1 mut E461 P12 b
Lamc1 mut E461 P12 a
Lamc1 mut E466 emb1 b
Lamc1 mut E466 emb1 a
Lamc1 mut E466 emb2 b
Lamc1 mut E466 emb2 b
Distance (μm)
0 50 100 150 200 250 300 350 400 450
time (min)
0 60 120 180 240 300 360 420

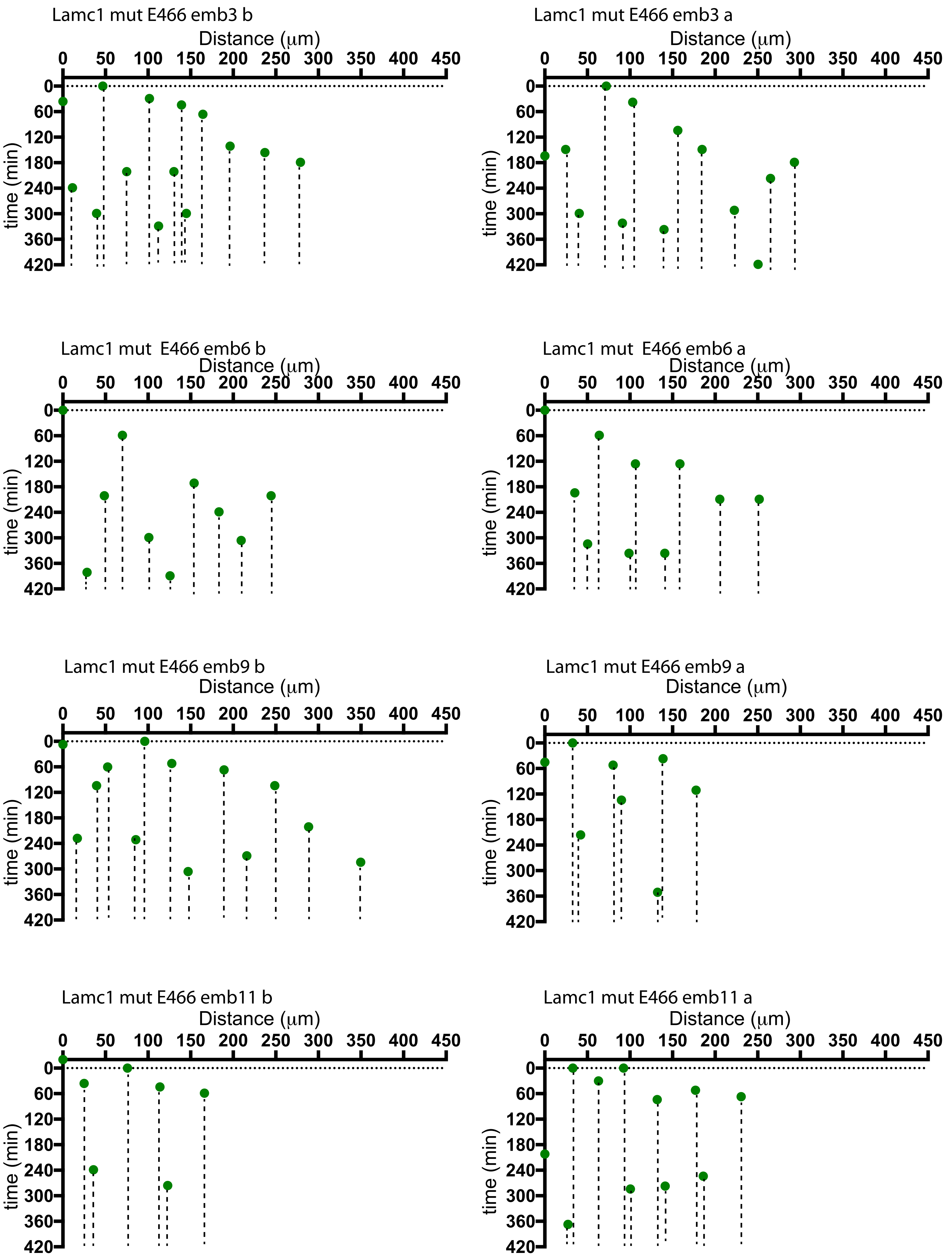
Lamc1 mut E466 emb3 b
Distance (μm)
0 50 100 150 200 250 300 350 400 450
time (min)
0 60 120 180 240 300 360 420
Lamc1 mut E466 emb3 a
Distance (μm)
0 50 100 150 200 250 300 350 400 450
time (min)
0 60 120 180 240 300 360 420
Lamc1 mut E466 emb6 b
Distance (μm)
0 50 100 150 200 250 300 350 400 450
time (min)
0 60 120 180 240 300 360 420
Lamc1 mut E466 emb6 a
Distance (μm)
0 50 100 150 200 250 300 350 400 450
time (min)
0 60 120 180 240 300 360 420
Lamc1 mut E466 emb9 b
Distance (μm)
0 50 100 150 200 250 300 350 400 450
time (min)
0 60 120 180 240 300 360 420
Lamc1 mut E466 emb9 a
Distance (μm)
0 50 100 150 200 250 300 350 400 450
time (min)
0 60 120 180 240 300 360 420
Lamc1 mut E466 emb11 b
Distance (μm)
0 50 100 150 200 250 300 350 400 450
time (min)
0 60 120 180 240 300 360 420
Lamc1 mut E466 emb11 a
Distance (μm)
0 50 100 150 200 250 300 350 400 450
time (min)
0 60 120 180 240 300 360 420

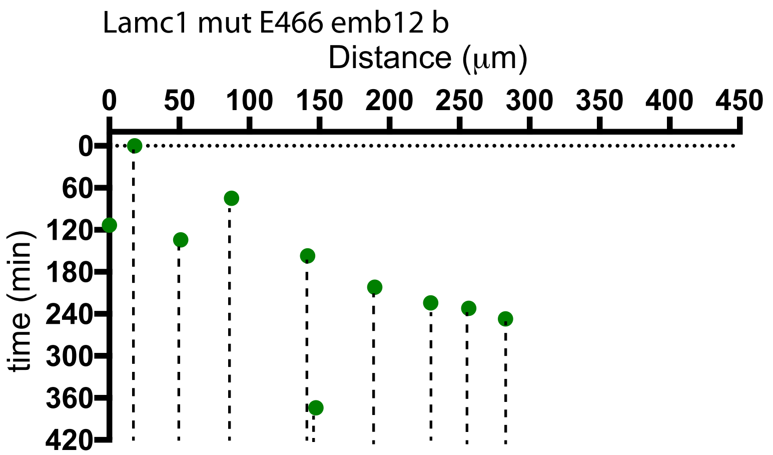
Lamc1 mut E466 emb12 b
Distance (μm)
0 50 100 150 200 250 300 350 400 450
time (min)
0 60 120 180 240 300 360 420

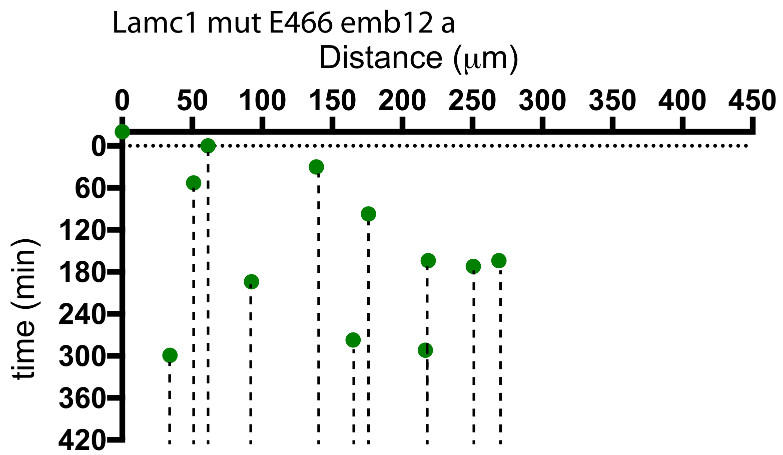
Lamc1 mut E466 emb12 a
Distance (μm)
0 50 100 150 200 250 300 350 400 450
time (min)
0 60 120 180 240 300 360 420
